# Life-history stage influences immune investment and oxidative stress in response to environmental heterogeneity in Antarctic fur seals

**DOI:** 10.1038/s42003-024-06499-6

**Published:** 2024-06-29

**Authors:** Rebecca Nagel, Katja Pohle, Lilla Jordán, Iva Tuponja, Claire Stainfield, Camille Toscani, Cameron Fox‑Clarke, David Costantini, Gábor Á. Czirják, Jaume Forcada, Joseph I. Hoffman

**Affiliations:** 1 Department of Evolutionary Population Genetics, Faculty of Biology, Bielefeld University, 33501 Bielefeld, Germany; 2 Department of Animal Behaviour, Bielefeld University, 33501 Bielefeld, Germany; 3 Department of Wildlife Diseases, Leibniz Institute for Zoo and Wildlife Research, 10315 Berlin, Germany; 4 British Antarctic Survey, High Cross, Madingley Road, Cambridge, CB3 OET UK; 5 Department of Ecological and Biological Sciences, University of Tuscia, 01100 Viterbo, Italy; 6 Center for Biotechnology, Faculty of Biology, Bielefeld University, 33615 Bielefeld, Germany; 7 Joint Institute for Individualisation in a Changing Environment, Bielefeld University and University of Münster, 33501 Bielefeld, Germany; 8 Present Address: School of Biology, University of St Andrews, St Andrews, KY16 9TH UK; 9 Present Address: Scotland’s Rural College, Craibstone Estate, Ferguson Building, Aberdeen, AB21 9YA UK

**Keywords:** Immunology, Ecology

## Abstract

Immune defenses are crucial for survival but costly to develop and maintain. Increased immune investment is therefore hypothesized to trade-off with other life-history traits. Here, we examined innate and adaptive immune responses to environmental heterogeneity in wild Antarctic fur seals. In a fully crossed, repeated measures design, we sampled 100 pups and their mothers from colonies of contrasting density during seasons of contrasting food availability. Biometric and cortisol data as well as blood for the analysis of 13 immune and oxidative status markers were collected at two key life-history stages. We show that immune responses of pups are more responsive than adults to variation in food availability, but not population density, and are modulated by cortisol and condition. Immune investment is associated with different oxidative status markers in pups and mothers. Our results suggest that early life stages show greater sensitivity to extrinsic and intrinsic effectors, and that immunity may be a strong target for natural selection even in low-pathogen environments such as Antarctica.

## Abstract (web summary)

An investigation into the immune profiles of Antarctic fur seals provides detailed insights into how organisms manage the allocation of and trade-offs among resources in a variable environment.

## Introduction

The immune system comprises multiple mechanisms to protect an individual against pathogens and parasites but can essentially be split into two components, the innate and adaptive systems. The cellular and humoral effectors of the innate system are the body’s first line of defense, initiating a broad and rapid cascade effect to clear foreign intruders^1,2^. The adaptive immune system, by contrast, is composed of a small number of cells and molecules generated de novo to target specific pathogens^1^. Although they differ mechanistically, both systems are essential for an effective immune response, so untangling how individuals manage the allocation of innate and adaptive immunity is important for understanding fitness variation and population dynamics.

A central assumption in eco-immunology is that a trade-off exists between investment in immune defense and other functions or activities, such as growth and reproduction^3^. This is particularly relevant for wild populations that experience variation in two of the most prominent environmental factors known to influence immune function – food availability and population density. When allocating finite energy resources, reduced food availability can constrain immune responses^4^, while the higher risk of pathogen transmission at higher population densities may induce density-dependent immune responses^5^.

When investigating life-history trade-offs, another layer of complexity is added when the performance of one activity has downstream consequences on another. For example, mounting an immune response, and in particular an inflammatory response, may lead to oxidative stress^6,7^, which in turn may constrain investment into growth and reproduction^8^. To understand how environmental factors impact traits closely linked with survival, it is therefore important to consider the complex network of mechanisms influencing life-history traits and their trade-offs.

**Fig. 1:**
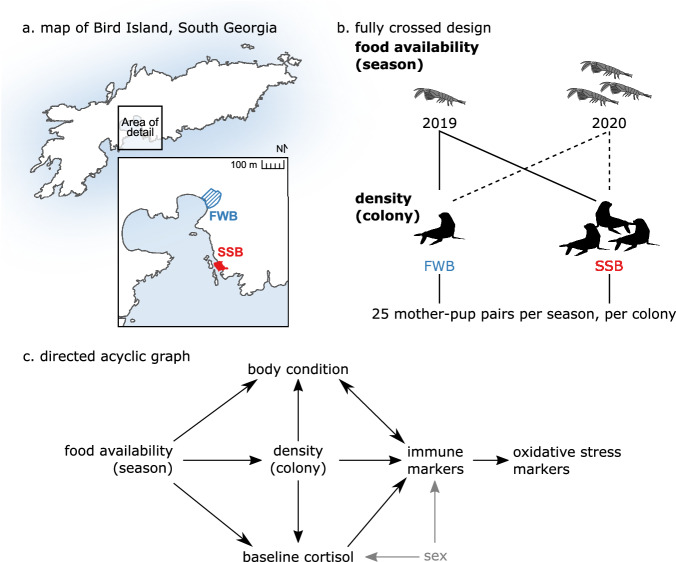
Summary of the study design. **a** Map of Bird Island, South Georgia. The area of detail shows the two study colonies, Freshwater Beach (FWB, blue stripes) and the Special Study Beach (SSB, red solid). **b** The study design was fully crossed. During two consecutive breeding seasons, we sampled 25 unique mother-pup pairs from both colonies, resulting in a final sample size of 100 mothers and 100 pups. All focal individuals were sampled twice, approximately 60 days apart, once at the beginning and again at the end of each season. In addition to immune and oxidative status markers, we gathered information on the sex, baseline cortisol level, and body condition of all individuals. **c** A directed acyclic graph (DAG) providing a visual representation of the hypothesized causal relationships among variables. Arrows denote possible direct causal effects. Sex was only included as a predictor variable in the models of the pups. Silhouette images are from http://phylopic.org and available for reuse under Creative Commons licenses.

Antarctic fur seals (*Arctocephalus gazella*) offer a unique opportunity to investigate these potential trade-offs in a wild mammalian population subjected to natural variation in food availability and population density. The availability of the fur seal’s staple diet, Antarctic krill, has been steadily declining over the past four decades due to local warming and the loss of sea ice, but food availability also fluctuates from year to year^9,10^. Furthermore, stable differences in population density exist among breeding colonies^11^. These characteristics are exemplified by our study population on the sub-Antarctic island of Bird Island, South Georgia. During two consecutive breeding seasons, the first of which had one of the lowest food availabilities ever recorded, we tracked 100 Antarctic fur seal pups and their mothers from two neighboring breeding colonies that experience comparable environmental conditions but which differ in population density (Fig. 1a, b)^12,13^. Focal pups and their mothers were sampled at two key life-history stages: shortly after birth and again approximately 60 days later, when molting commences. The molt is a particularly important life history stage, as molting is a costly process and signifies pups gaining nutritional and behavioral independence. At these two time points, we collected blood for the analysis of nine immune and four oxidative status markers (Table 1) as well as biometric and baseline salivary cortisol data.

**Table 1:** Brief overview of the immune and oxidative status markers measured in Antarctic fur seal pups and mothers

Immune marker	Effector type	Immune defense	Functions	Relative costs
BKA: *S. aureus*	Humoral	Constitutive innate	BKA is a measure of the overall function of the constitutive innate immune response; it quantifies the ability of plasma to fight bacteria (*S. aureus* gram-positive; *E. coli* gram-negative)	Low energetic and pathological costs
BKA: *E. coli*				
Hemagglutination	Humoral	Constitutive innate	Natural antibodies that neutralize pathogens	Low energetic and pathological costs
Hemolysis	Humoral	Constitutive innate	Complement titers causing cell wall rupture of foreign cells (e.g., lysis)	Low energetic and pathological costs
Lysozyme	Humoral	Constitutive innate	Catalyzes the lysis of gram-positive bacteria	Low energetic and pathological costs
Haptoglobin	Humoral	Induced innate	Inhibits oxidative activity, antibacterial and immunomodulatory activity	High energetic and pathological costs
Neopterin	Humoral	Innate & adaptive	Released by activated macrophages; indicative of pro-inflammatory immune responses	High energetic and pathological costs
IgG	Humoral	Adaptive	Immunological memory; bind and neutralize pathogens or mark them for phagocytosis	High energetic and low pathological costs
WBC: neutrophil	Cellular	Innate	Phagocytic leukocyte	High energetic and low pathological costs
WBC: basophil	Cellular	Innate	Autoimmunity	High energetic and pathological costs
WBC: eosinophil	Cellular	Innate & adaptive	Defense against parasites	High energetic and low pathological costs
WBC: lymphocyte	Cellular	Innate & adaptive	Natural killer cells & T-cells, B-cells; lysis of infected cells and recognition of pathogens; production of cytokines and toxic granules; secretion of antibodies	High energetic and low pathological costs
WBC: monocyte	Cellular	Innate & adaptive	Phagocytosis, antigen presentation, cytokine production	High energetic and pathological costs

Oxidative status marker			Functions	
OXY			Non-enzymatic antioxidant capacity	
dROM			Plasma oxidative damage	
GPx			Prevents oxidation due to hydrogen peroxide and organic hydroperoxides	
SOD			Prevents oxidation due to superoxide radicals	

Immune defense is divided into the innate and adaptive systems, broadly representing immediate, non-specific versus slower, acquired immunity. Constitutive mechanisms prevent infection, while induced defenses are those that act after infection. Please see ref. ^25^.

*BKA* bacterial killing assay, *Ig* immunoglobulin, *WBC* white blood cell, *OXY* antioxidant capacity, *dROMs* reactive oxygen metabolites, *GPx* glutathione peroxidase, *SOD* superoxide dismutase.

This unique ‘natural experiment’ allowed us to investigate relationships among environmental variation, trait variation, immunity, and oxidative stress. First, to provide a baseline, we characterized ontogenetic changes in the immune responses of pups in comparison to their mothers. We then tested the following hypotheses: (i) investment into immunity depends on life history stage. Specifically, we hypothesized that there would be more variation in the immune responses of pups, both because they have fewer resources available to buffer them from detrimental environmental conditions, and because they have not previously been exposed to parasites and pathogens, whereas their mothers have immune memory; (ii) immune responses are influenced by environmental variation, with investment into immunity being negatively impacted by food stress but positively impacted by population density due to the assumed higher prevalence of parasites and pathogens; (iii) immune investment trades off with other life-history traits. Specifically, we hypothesized that elevated levels of the stress hormone cortisol and poorer body condition suppress immune defenses; and (iv) immune investment leads to oxidative stress under the hypothesis that reactive oxygen species are generated during immune activation (e.g., oxidative burst) and that resources are limited and a trade-off exists between immune function and antioxidant defenses (Fig. 1c).

## Results

We investigated the direct and total effects of food availability (season), density (colony), body condition, baseline cortisol, and sex on nine immune markers, as well as the effect of these immune phenotypes on four markers of oxidative status in a wild population of Antarctic fur seals. Our final dataset comprised of 98 pups and 97 mothers randomly sampled from two neighboring breeding colonies of contrasting population density (Fig. 1a) over two consecutive breeding seasons of contrasting food availability (Fig. 1b). Repeated measures were successfully gathered from 67 pups and 80 mothers. The range, first and third quartiles, median, mean, and total number of missing values for all data can be found in Supplementary Tables S1 and S2. The raw data are plotted in Supplementary Figs. S1–S18. Full summaries of the model outputs, including point estimates of the posterior mean and 95% highest posterior density intervals (HPDIs), effective sample sizes, and *p*MCMC values for each predictor variable are provided in Supplementary Data Tables S1 (pups) and S2 (mothers).

### Repeatability

Immune marker levels showed modest repeatability from birth to molt (i.e., point estimates for *R* > 0.1) and repeatability was similar across all of the immune markers, suggesting that individuals differ in their immune profiles. The 95% posterior intervals were correspondingly large (Supplementary Table S4). In pups, the mean variance explained by repeated sampling of the same individual ranged from a high of 0.27 [95% HPDI: 0.12−0.42] for haptoglobin to a low of 0.14 [95% HPDI: 0.07−0.21] for hemolysis. In mothers, the range was 0.49 [95% HPDI: 0.26−0.72] for neopterin to 0.13 [95% HPDI: 0.07−0.20] for BKA (*S. aureus*). Repeatability estimates from the null model were comparable and can be found in Supplementary Table S5.

### Development of immunity and oxidative status over time

BKA (*E. coli*), hemagglutination, hemolysis, IgG, and dROM levels increased significantly in pups from birth to molt (respective Cohen’s *d* = −0.62, −2.11, −2.18, −2.06, and −1.59), while lysozyme and neopterin concentrations decreased (respective Cohen’s *d* = 0.64 and 1.19). In mothers, of the nine immune markers assayed, only the BKA (*S. aureus*) and the innate/adaptive WBC ratio changed significantly between the sampling time points, both showing a decrease over the approximately 60 days between giving birth and starting to wean their pup (Cohen’s *d* = 1.14 and 0.77). SOD concentrations increased moderately between the sampling time points (Cohen’s *d* = 0.53) (Supplementary Table S6, Supplementary Fig. S1).

At birth, differences between pup and mother immune and oxidative stress concentrations were moderate to large (Cohen’s *d* > |0.5|) for all of the markers except lysozyme and OXY. This difference between the two group means was notably reduced by the time pups started to molt and gain nutritional independence. BKA (*S. aureus*), hemagglutination, hemolysis, lysozyme, IgG, the innate/adaptive WBC ratio, OXY, and dROM all showed a difference of less than 0.5 standard deviations. This suggests that immune and oxidative status marker concentrations in pups converged on the adult phenotype before or around the time of increasing independence, as pups began to molt (Supplementary Table S6, Supplementary Fig. S1).

### Comparison of stress markers

There was no association between the neutrophil/lymphocyte ratio and baseline salivary cortisol in our full dataset of pups and mothers (*r* = −0.05, 95% CI = −0.22−0.12, *t* = −0.62, df = 130, *p* = 0.54) (Supplementary Fig. S19). Although the neutrophil-lymphocyte ratio has been cited as a potential indicator of chronic stress in multiple mammals^14^, our results add to the growing body of evidence that these two metrics may not be interchangeable assessments of stress (reviewed in ref. ^15^).

### Immunity in pups

**Fig. 2:**
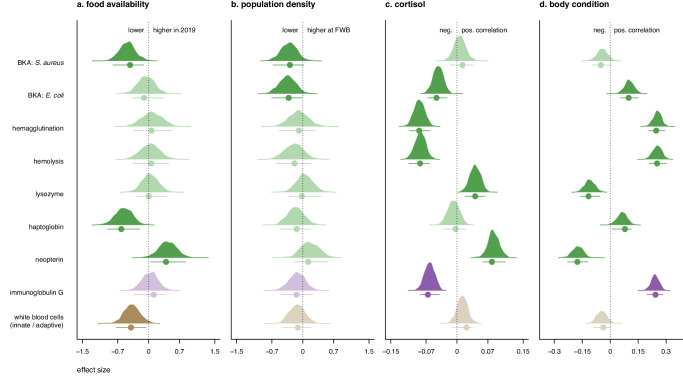
Density curves showing the total effect of each predictor variable for the nine immune markers measured in pups. BKA (*S. aureus* and *E. coli*), hemagglutination, hemolysis, lysozyme, haptoglobin, neopterin, immunoglobulin G, and the adaptive/innate white blood cell ratio. Posterior distributions of the relative probabilities and the posterior mode ± 95% highest posterior density intervals (HPDI) are shown for (**a**) food availability (2019 relative to 2020); (**b**) population density (FWB relative to SSB); (**c**) baseline salivary cortisol; and (**d**) body condition. The total effect of pup sex on immune marker levels was also assessed; all point estimates were close to zero with 95% HPDIs overlapping zero (Supplementary Data Table S1). Green: adaptive immune markers; Purple: innate immune markers; Brown: adaptive/innate ratio. When *p*MCMC < 0.05, posterior distributions are shown in bold.

The direct and total effect of pup sex on immune marker levels was weak, with all of the point estimates being close to zero and the 95% HPDIs overlapping zero (Supplementary Data Table S1). The proportion of BKA (*S. aureus*) and haptoglobin as well as the ratio of innate/adaptive WBCs were significantly lower in 2019 than 2020 and thus had lower values in the season of low food availability. By contrast, neopterin concentrations were higher in 2019 (Fig. 2a). Population density had a total effect on BKA (*S. aureus* and *E. coli*), with point estimates for both assays being comparatively higher in individuals born at SSB, the high-density colony (Fig. 2b). We also found a positive correlation between baseline salivary cortisol and two immune markers, neopterin and lysozyme, while cortisol was negatively correlated with four immune markers, BKA (*E. coli*), hemagglutination, hemolysis, and IgG (Fig. 2c). The exact opposite was found for body condition in pups, which was positively correlated with BKA (*E. coli*), hemagglutination, hemolysis, haptoglobin, and IgG, and was negatively correlated with neopterin and lysozyme (Fig. 2d).

### Immunity in mothers

**Fig. 3:**
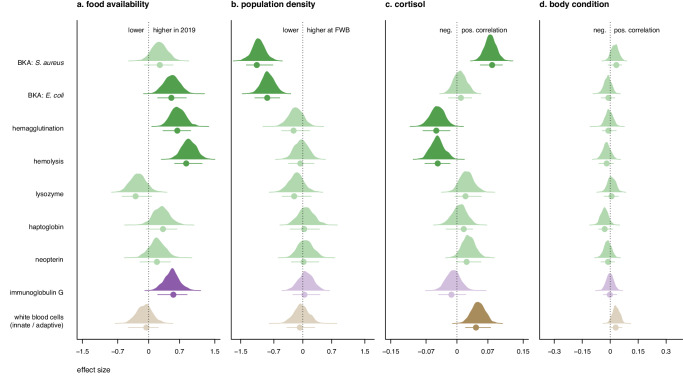
Density curves showing the total effect of each predictor variable for the nine immune markers measured in mothers. BKA (*S. aureus* and *E. coli*), hemagglutination, hemolysis, lysozyme, haptoglobin, neopterin, immunoglobulin G, and the adaptive/innate white blood cell ratio. Posterior distributions of the relative probabilities and the posterior mode ± 95% highest posterior density intervals (HPDI) are shown for (**a**) food availability (2019 relative to 2020); (**b**) population density (FWB relative to SSB); (**c**) baseline salivary cortisol; and (**d**) body condition. Green: adaptive immune markers; Purple: innate immune markers; Brown: adaptive/innate ratio. When *p*MCMC < 0.05, posterior distributions are shown in bold.

The concentrations of BKA (*E. coli*), hemagglutination, hemolysis, and IgG were significantly higher in 2019, the year of low food availability (Fig. 3a). Population density had a total effect on BKA (*S. aureus* and *E. coli*), with point estimates for both assays being higher in individuals breeding at SSB, the high-density colony (Fig. 3b). We found a significant positive correlation between baseline cortisol and two immune markers, BKA (*S. aureus*) and the innate/adaptive WBC ratio. By contrast, negative correlations were found between cortisol and both hemagglutination and hemolysis (Fig. 3c). Associations between maternal body condition and immune markers were weak, with all of the point estimates being close to zero and the 95% HPDIs overlapping zero (Fig. 3d).

### Oxidative stress

**Fig. 4:**
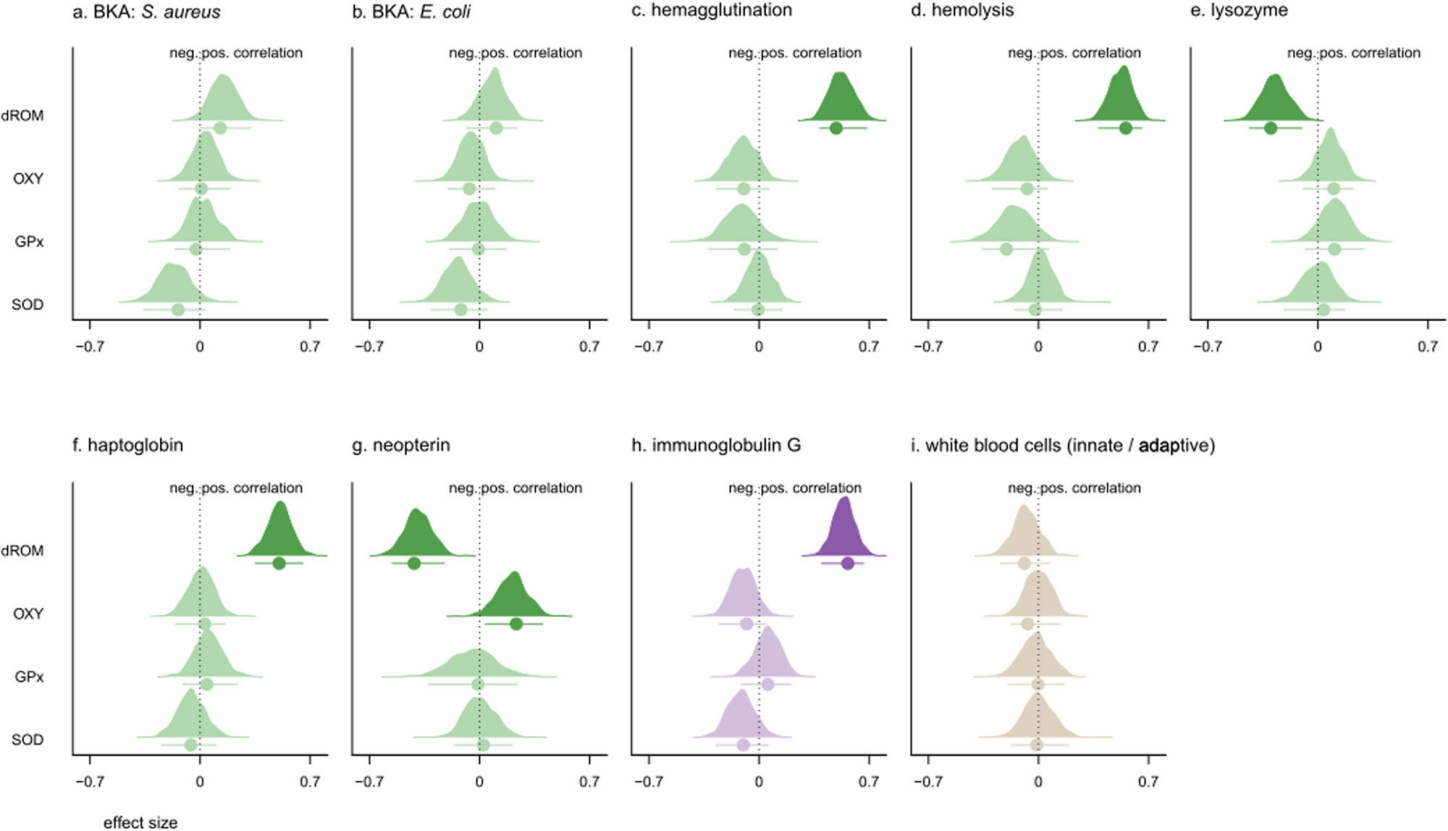
Density curves showing the total effect of each predictor variable on the four oxidative status markers measured in pups. **a** BKA (*S. aureus*); (**b**) BKA (*E. coli*); (**c**) hemagglutination; (**d**) hemolysis; (**e**) lysozyme; (**f**) haptoglobin; (**g**) neopterin; (**h**) immunoglobulin G; (**i**) adaptive/innate white blood cell ratio. Posterior distributions of the relative probabilities and the posterior mode ± 95% highest posterior density intervals (HPDI) are shown. Green: adaptive immune markers; Purple: innate immune markers; Brown: adaptive/innate ratio. OXY, antioxidant capacity; dROMs, reactive oxygen metabolites; GPx, glutathione peroxidase; SOD, superoxide dismutase. When *p*MCMC < 0.05, posterior distributions are shown in bold.

**Fig. 5:**
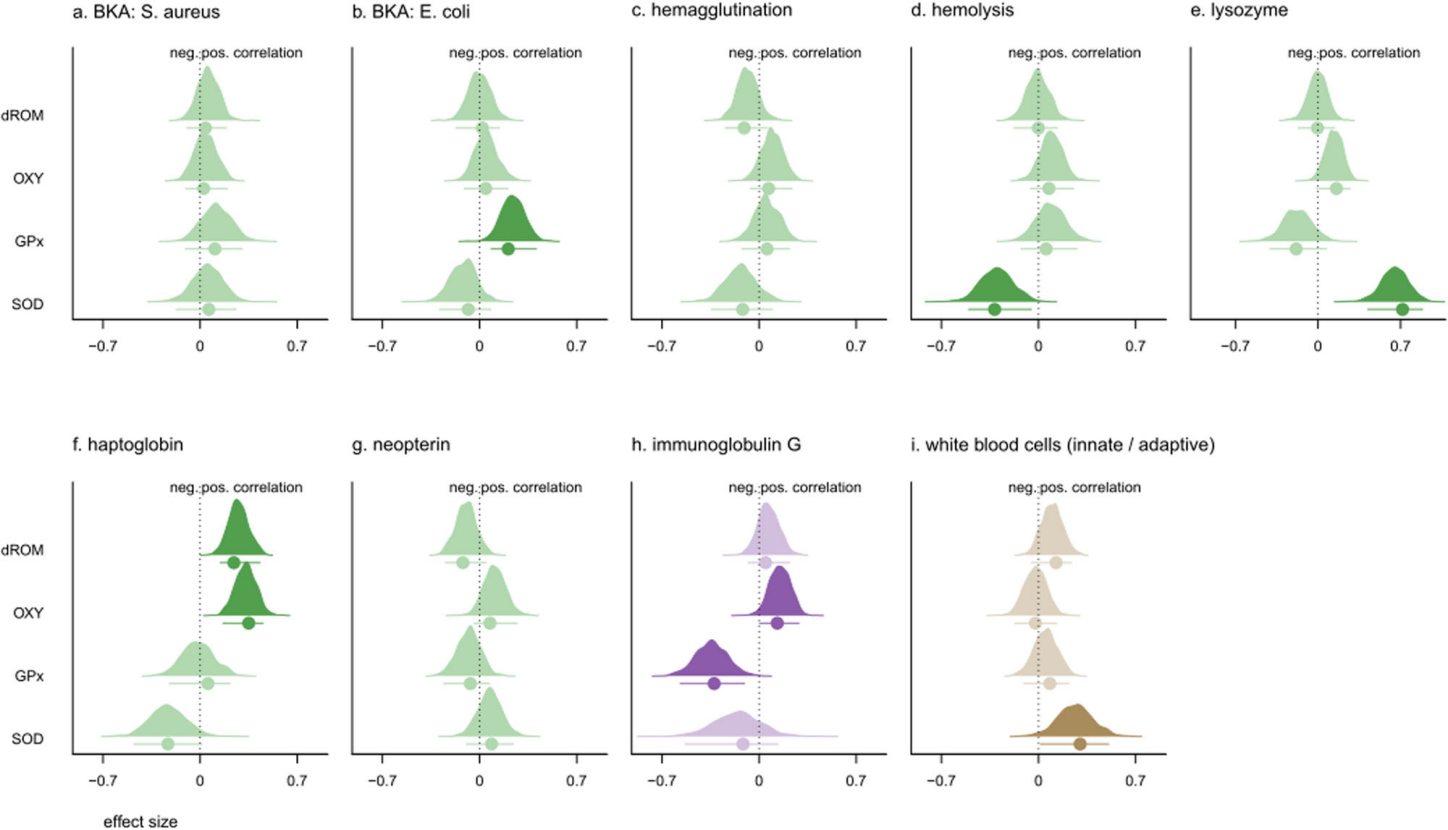
Density curves showing the total effect of each predictor variable on the four oxidative status markers measured in mothers. **a** BKA (*S. aureus*); (**b**) BKA (*E. coli*); (**c**) hemagglutination; (**d**) hemolysis; (**e**) lysozyme; (**f**) haptoglobin; (**g**) neopterin; (**h**) immunoglobulin G; (**i**) adaptive/innate white blood cell ratio. Posterior distributions of the relative probabilities and the posterior mode ± 95% highest posterior density intervals (HPDI) are shown. Green: adaptive immune markers; Purple: innate immune markers; Brown: adaptive/innate ratio. OXY, antioxidant capacity; dROMs, reactive oxygen metabolites; GPx, glutathione peroxidase; SOD, superoxide dismutase. When *p*MCMC < 0.05, posterior distributions are shown in bold.

In pups, neopterin was the only immune marker to correlate with OXY. None of the immune markers correlated significantly with GPx or SOD. By contrast, the majority of immune markers were associated with dROM. Specifically, hemagglutination, hemolysis, haptoglobin, and IgG all correlated positively with dROM levels, while neopterin and lysozyme were negatively correlated (Fig. 4). In mothers, the innate immune marker haptoglobin correlated positively with both OXY and dROM, while the adaptive immune marker IgG correlated positively only with OXY. BKA (*E. coli*) correlated positively, and IgG correlated negatively with GPx. Lysozyme and the innate/adaptive WBC ratio both correlated positively with SOD, while hemolysis showed a negative correlation (Fig. 5).

## Discussion

The trade-off between immune defense and other life-history traits may be influenced by any number of extrinsic and intrinsic factors. Investigating broad, cross-scale links among environmental variation, trait variation, immunity, and oxidative stress in wild populations is therefore essential for understanding the dynamics of immune activity and its influence on population dynamics. Here, we show that immune defense profiles are strongly dependent on the physical and social environment, and vary with life-history stage in Antarctic fur seals. Developing offspring are especially sensitive to environmental variation (i.e., food availability and population density) and invest differentially into immune defense depending on their cortisol levels and body condition. Furthermore, immune investment is strongly correlated with oxidative stress in pups, but not in mothers. Together, these results suggest that offspring pay a greater cost of immune defense. By implication, immunity appears to be a strong target for natural selection, even in polar environments which are believed to carry fewer pathogens^16^. Our findings lend support to calls for more long-term, longitudinal research into the evolutionary trajectories of immune defenses in wild populations^17^. This will become increasingly important as anthropogenic climate change creates ever more extreme and variable environmental conditions^18^.

Previous work on the ontogeny of immunity in various pinniped species has found that antibody levels differ between pups and adults at birth, but converge as pups approach their molt^19–24^. We obtained similar results, with pups and mothers differing significantly at nine of the eleven markers at birth, while only haptoglobin and neopterin remained different as the pups approached nutritional independence. Both haptoglobin and neopterin represent induced innate immune responses with high energetic and pathological costs but, while haptoglobin levels remained lower in pups relative to mothers throughout this study, neopterin levels were consistently higher. These patterns are indicative of antibacterial preparedness and higher cellular immune activation during early development, a time when individuals are exposed to various novel pathogens, especially bacteria, which are one of the main causes of death in this population^24^.

In line with our hypothesis that individuals experiencing the highest energetic demands should down-regulate the costliest defenses^25^, we found that Antarctic fur seal pups tended to invest less into immune responses under conditions of food stress. The only exception was neopterin, which had higher concentrations in pups during the year of lower food availability. If food-limited individuals prioritize other energetically demanding processes in lieu of immunity, they may become more susceptible to infections^26^. Given that neopterin is a byproduct of macrophage activity and an indicator of immune activation due to infection^27^, elevated levels of neopterin may indicate an increase in susceptibility to infection when food is scarce.

Contrary to our expectations, adult females showed increased investment into constitutive innate and adaptive immunity under food stress. This is surprising because undernourished individuals should have fewer resources to mount a strong immune response^4^. One possible explanation for this finding could be that breeding females were not necessarily under-nourished in the year of low food availability. This is supported by previous findings showing no differences in body condition between mothers in the two consecutive years of our study^12^. Alternatively, our results are consistent with food-limited individuals prioritizing investment into those components of the immune system that carry the lowest energetic and pathological costs (e.g., constitutive innate and antibody-mediated adaptive immunity).

We found that both life history stages responded similarly to higher population density, with pups and their mothers investing more into constitutive innate immunity. Individuals experiencing higher population densities may be more frequently exposed to parasites and pathogens^5,28^ such that maintaining high levels of basal immunity can be beneficial, despite potential trade-offs with other life-history components^29^. In other words, the relative threat of infection at higher density may be mirrored by the status of the immune system. However, despite this general pattern, we did not find an association between population density and IgG concentration, an adaptive immune marker that reflects cumulative exposure to pathogens over time^25^ and which is therefore typically elevated at high density^30,31^. The lack of association in our study could potentially be a reflection of the Antarctic setting, as a previous study of three Antarctic penguin species found that immunoglobulin levels increase northwards along a latitudinal gradient^32^. Alternatively, it is possible that our study was too short to detect cumulative increases in IgG, especially as the mothers spent much of their time foraging at sea.

None of the immune markers varied between male and female pups. Although contrary to our initial expectations, sex differences in immunity may only become evident after individuals reach sexual maturity and selection pressures on the sexes diverge^33^. This is supported by evidence from gray seal pups^34^ but contradicts previous findings in Galapagos sea lion pups^23^, implying that sex-specific responses are species- and/or context-dependent.

At both life history stages, we found null and negative correlations between baseline cortisol and the majority of immune markers, in line with the known inhibitory effects of glucocorticoids on the immune system^35^. There were, however, two notable exceptions. First, neopterin was positively correlated with baseline cortisol in pups. This is surprising given that neopterin is a byproduct of macrophage activity, and cortisol suppresses inflammation mediated by macrophages^35^. One possible explanation is that our measure of cortisol from saliva reflects the short-term stress response, which can enhance the expression of immune responses^36^. Alternatively, chronically elevated cortisol may increase susceptibility to infection and disease^36^ such that increased levels of neopterin indicate higher cellular immune activation under long-term stress. Second, BKA (*S. aureus*) was positively correlated with baseline cortisol in adult females. This might again be related to a positive effect of short-term stress responses on the immune system^36^, translated here as an upregulation of functionality (BKA, innate WBCs) and a downregulation of natural antibodies (agglutination).

We further found that the relationship between immune response and body condition differs between pups and mothers. In pups, most of the immune markers were positively correlated with body condition. This observation builds upon a large body of evidence from wild populations^37^, including pinnipeds^23,38,39^, suggesting that well-nourished individuals can mount a stronger immune response. We also found that those immune markers that were positively correlated with cortisol were negatively associated with body condition, and vice versa. This may indicate condition-dependent trade-offs between stress responses and immune investment, both of which are energetically demanding processes. In contrast to pups, no association was found between body condition and immunity in mothers. This is likely due to selection bias, as only those adult females that successfully carried a pup to term were included in our study, meaning that only mothers in relatively good condition were sampled. Consistent with this explanation, we previously reported no differences in quality measures (body condition, span, girth, weight, length) between mothers from the two breeding colonies and between the two years^12^.

We found a strong relationship between immunity and oxidative status in the pups. In particular, diverse components of the immune system were found to correlate with dROM, including both innate and adaptive immune markers. Reactive oxygen metabolites (ROMs) are by-products of the ROS-induced oxidation of organic substrates and their levels in the plasma often increase during an immune response^40^. Our results are therefore consistent with previous research showing that oxidative stress increases when individuals mount an immune response^6–8,40^. However, the opposite pattern was again observed for neopterin, suggesting that non-enzymatic antioxidant capacity (OXY) is increased and oxidative damage (dROM) is decreased in pups in response to elevated macrophage activity. Moreover, levels of the two antioxidant enzymes glutathione peroxidase (GPx) and superoxide dismutase (SOD) did not correlate with any of the immune markers, suggesting that these two enzymes were not upregulated in response to increased ROMs. The release of ROS by phagocytes is most commonly associated with bacterial killing. However, activated macrophages usually produce far lower levels of ROS than neutrophils, and antioxidants play an important role in mitigating oxidative damage, such as ROMs^41^, which might explain the associations observed in pups between ROMs and other immune markers. Thus, our results lend support to the notion that individuals invest more into innate cellular immune defense during early development, when they first encounter parasites and/or pathogens.

Contrary to our expectations, as well as our results for the pups, the majority of immune markers were not associated with our marker of oxidative damage in mothers. By contrast, we found several significant correlations between immune markers and non-enzymatic antioxidant capacity, GPx, and SOD. This is at odds with a recent meta-analysis, which found that the effect of the immune response on oxidative stress was similar in juveniles and adults^7^. Our results could potentially be explained by the metabolic demands of growth, which have been shown to significantly increase oxidative damage^8^. Given that Antarctic fur seals grow most rapidly during the time from birth to weaning^42^, this trade-off is expected to be more pronounced in pups than mothers. Another explanation might lie with the immaturity of the antioxidant system of pups, particularly in terms of the expression of antioxidant enzymes, as previously described in hooded seals (*Cystophora cristata*) and in other vertebrates^43,44^. In our case, this was particularly evident for GPx due to higher levels in adults than in pups. This is relevant because GPx detoxifies tissues from the accumulation of certain oxidized molecules quantified with our dROMs assay. Although the lack of upregulation of antioxidant enzymes came at a cost in terms of oxidative damage, it might have been advantageous to mount an effective immune response. Indeed, the generation of ROS during the oxidative burst in neutrophils is one important component of innate immunity. Both SOD and GPx remove certain ROS that are also generated during the oxidative burst. Thus, in order to not compromise an already naïve immune response, pups appeared to make the best of a bad job keeping the expression of these two enzymes low.

Our study exploited a unique natural experiment in combination with an unusually large panel of immune and oxidative status markers. Our fully crossed, repeated measures design allowed us to investigate life-history tradeoffs in the context of the social and physical environment, while our diverse panel of markers provided detailed insights into multiple components of the vertebrate immune system and their relationship with oxidative stress. Arguably the strongest criticism of our work relates to the correlative nature of our results. However, our Bayesian network (Fig. 1c) was based on a pre-defined set of causal hypotheses from the literature, such that likely causal relationships among the variables could be reasonably estimated. Furthermore, although antigen challenge experiments would in principle be possible, they are not without drawbacks^45^, and experimental manipulations of wild populations have ethical implications^46^ as well as the potential to disrupt natural host-parasite interactions and population dynamics^47^. These issues could be problematic for our study system, where long-term monitoring would be disrupted by invasive experimentation.

In conclusion, our results suggest that the immune profiles of developing offspring are more sensitive to both extrinsic and intrinsic factors than those of adults. Exposure to novel parasites and pathogens during this early life stage likely imposes strong selective pressures, such that only those individuals capable of coping with exposure to diverse pathogens survive to reproduce. Our results further suggest that a complex array of immune effectors control infections during early development. Consequently, natural selection appears to have favored diversity in the detection, control, and elimination of pathogens.

## Methods

### Experimental design

This study was conducted at Bird Island, South Georgia (54°00′24.8ʺS, 38°03′04.1ʺW), a sub-Antarctic island located in the southern Atlantic Ocean. During the 2018–19 (hereafter 2019) and 2019–20 (hereafter 2020) breeding seasons (December to March), 25 unique mother-pup pairs were sampled from two neighboring colonies, Freshwater Beach (FWB) and Special Study Beach (SSB) (Fig. 1a, b). Sampling was randomized with respect to pup sex, resulting in a total of 51 male and 49 female focal pups. Pup mortality averaged 25.6% over the two seasons and colonies but was overall higher at FWB (32%) than SSB (12%)^12^.

Mothers and pups were sampled together twice, around 2–3 days after birth (denoted as age = 0 in the raw data and Supplementary Table S1) and approximately 60 days thereafter, as the pups began to molt and gain nutritional independence. Given that the presence or absence of the mother could impact body condition and blood metabolites at the time of second sampling, mothers and pups were sampled concurrently on the first day that the pup was seen together with its mother post-60 days of age. For sampling, we followed animal handling protocols established and refined by the British Antarctic Survey over their 40-year Antarctic fur seal monitoring and survey program. In brief, adult females were captured with a noosing pole and immobilized on a restraint board. Pups were captured with a slip noose or by hand and were restrained by hand. Individuals were released as close to their capture site as possible and the pups were reunited with their mothers. When a focal individual was captured, weight and length measurements were taken, which were used to calculate body condition after^48^. Saliva was also collected to assess baseline cortisol concentrations; for a more detailed description of our saliva sampling methods, cortisol concentration assessment, and analysis protocols, see ref. ^49^.

For the analysis of immune and oxidative status markers, we collected up to 2.5 ml of blood from the hind flipper using BD Discardit Eccentric Luer-Slip two-piece syringes and BD Microlance stainless steel needles (25 G, 0.5 × 25 mm). Blood was immediately transferred to Greiner Bio-One Plasma tubes coated with lithium heparin to prevent clotting. One drop of blood was used to prepare a smear on a glass microscope slide (76 × 26 mm, cut edges, Menzel). The remaining blood was centrifuged at 1000 × *g* for ten minutes to separate the plasma from the blood cells. Samples were stored in 200 µL aliquots at −20 °C in the field and during transport, before being transferred to −80 °C in the laboratory. Although 18−20 gauge needles are recommended for blood collection in otariids^50^, the use of smaller gauge needles did not hinder our analysis in terms of blood clotting or hemolysis. Hemolysis was noted in the field whenever detected (*n* = 17 or 4% of the total samples) and these samples were assessed during laboratory and data analysis for possible effects.

### Immune and oxidative status assays

Humoral and cellular components of the innate and adaptive immune system (Table 1) were quantified using established assays that have been successfully applied to wild mammals e.g., refs. ^51,52^, including pinnipeds e.g., refs. ^20,23,38,39,53–55^. Additionally, we measured one marker of plasma oxidative damage, one marker of plasma non-enzymatic antioxidant capacity, and plasma levels of two antioxidant enzymes (superoxide dismutase, SOD, and glutathione peroxidase, GPx) using standard methods applied to a variety of wildlife species^40,56^.

#### Bacterial killing assays

We measured the overall function of the constitutive innate immune system by assessing the in vitro bacterial killing activity of the plasma against the gram-negative *Escherichia coli* (ATCC #8739) and gram-positive *Staphylococcus aureus* (ATCC # 6538 P) using a liquid growth inhibition assay modified from^57^. The samples were diluted 1:2 with TSB before 5 µL of each diluted sample was added to a flat-bottomed 96-well plate. The fresh bacterial cultures were diluted in TSB until the attenuance (*D*) at 595 nm was 0.01 and 45 µL was then added to each well. We mixed 45 µL of bacterial solution with 5 µL of TSB as a positive control and used 50 µL of TSB only as a negative control. The plates were incubated at 37 °C for 20 h and the *D* at 595 nm was read in a plate reader (Biotek) before and after incubation. After subtracting the *D* of samples at 0 h of incubation from the ones at 20 h of incubation, for each microbial strain the bacterial killing activity of was defined as the percent of the killed bacteria, which was calculated as 1 – (*D*_sample_/*D*_positive control_).

#### Hemolysis–hemagglutination assay

Natural antibodies, measured as hemagglutination (HA) titers, bind non-specifically to various antigens and have important roles in opsonization. Hemolysis (HL), mediated by complement, is part of the innate immune system and its activation results in cell lysis, especially those previously opsonized. Levels of HA and HL were assessed using the hemolysis–hemagglutination assay as described by ref. ^58^ modified for mammals by using chicken red blood cells as a target^52,59^. After pipetting 12.5 µL of plasma into the first two columns of a U-shaped 96-well microtitre plate, 12.5 µL sterile PBS were added to columns 2–12. The content of the second column of wells was serially diluted (1:2) until the 11th column, resulting in a dilution series for each sample from 1/1 to 1/1024. The last column of the plate was used as a negative control, containing only PBS. 12.5 µL of 1% chicken red blood cell suspension (supplied by Dunnlab) was added to all of the wells and incubated at 37 °C for 90 min. After incubation, to increase the visualization of agglutination, the plates were tilted at a 45° angle at room temperature. Agglutination and lysis were recorded after 20 and 90 min, respectively. Titers of HA and HL were given as the log_2_ of the reciprocal of the highest dilution of plasma showing complete hemagglutination or hemolysis, respectively^52,58^.

#### Lysozyme concentration

Lysozyme is an enzyme with antibacterial activity, especially against gram-positive bacteria. To measure the concentration of lysozyme, we used the lysoplate assay method^52,55^. 4 µL of the sample was inoculated in the test holes of a 1% Noble agar gel (A5431, Sigma) containing 25 mg/100 ml lyophilized *Micrococcus lysodeikticus* (M3770, Sigma), a bacterium that is particularly sensitive to lysozyme concentration. Crystalline hen egg white lysozyme (L6876, Sigma) (concentration: 0.5, 0.8, 1, 2, 4, 8, 10, 20, 40 and 100 µg/ml) was used to prepare a standard curve for each plate. Plates were incubated at 37 °C for 20 h. During incubation, a clear zone developed in the area of the gel surrounding the sample inoculation site corresponding the bacterial lysis. The diameters of the cleared zones are proportional to the log of the lysozyme concentration. This area was measured three times digitally using the software ImageJ (version 1.48, https://imagej.nih.gov/ij/) and the mean was converted to a semi-logarithmic plot into hen egg lysozyme equivalents (HEL equivalents, expressed in µg/ml) according to the standard curve^52^.

#### Haptoglobin concentration

Haptoglobin is an acute phase protein that usually occurs at low concentrations, but its production and secretion are increased in response to acute infections and trauma. To measure the concentration of haptoglobin, the standard procedure of the commercial kit “PHASE”^TM^ Haptoglobin Assay (Tridelta, Ireland) was followed, an assay previously used in various pinniped species (e.g., refs. ^39,53^). After diluting the plasma samples (1:2) with PBS, hemoglobin was added. Haptoglobin binds to hemoglobin and maintains its peroxidase activity at a low pH. The measured peroxidase activity of hemoglobin is directly proportional to the amount of haptoglobin in the sample. Haptoglobin concentrations were calculated according to the standard curve on each plate and were expressed as mg/ml. If the values measured were higher than the highest standard, the out-of-range samples were diluted and reanalyzed.

#### Neopterin concentration

Neopterin is synthesized primarily by activated macrophages and is considered a biomarker for the activation of the cellular Th1 immune response. To measure the concentration of neopterin, we used a commercial neopterin ELISA kit (#RE59321; IBL International GmbH, Germany). Following the manufacturer’s protocol, 20 µL of standards, commercial controls, and undiluted plasma samples were added in duplicate into wells of the microtiter plate coated with a goat anti-rabbit antibody. We added 100 µL of enzyme conjugate and 50 µL of neopterin antiserum to each well. We covered the microtiter plate with black adhesive foil and incubated it by gently vortexing the plate in the dark for 90 min. After incubation, we removed the adhesive foil and washed the plate four times with 300 µL of diluted wash buffer. We then added 150 µL of substrate solution into each well and incubated the plate for 10 min. We stopped the reaction by adding 150 µL of stop solution into each well. Absorbance was read at 450 nm (Biotek; µQuant Microplate Spectrophotometer). The final concentrations were extrapolated from the standard curve for each plate and expressed in nmol/L.

#### Protein A ELISA to measure immunoglobulin G concentrations

We measured the concentration of immunoglobulin G (IgG), the most common antibody subtype, in plasma with a Protein A ELISA^19,34^. Briefly, high-binding 96-well plates were coated with 100 µl diluted plasma samples (1:20,000 in 50 mM NaHCO_3_, pH 9.5) in duplicate and incubated for 1 h at 37 °C. Purified canine IgG (#0129-01, Southern Biontech) (concentration from 0.0625 µg/ml to 5 µg/ml) was used to prepare a standard curve for each plate. After incubation, the plates were washed in Tris-buffered saline/Tween-20 (TBS-T), blocked with 1% gelatin (Merck) solution and incubated for 30 min at 37 °C. After washing, 100 µl of Protein A-horseradish peroxidase conjugate solution (Invitrogen, 1:2000 in TBS-T, pH 7.4) was added to each well. The wells were washed after 30 min incubation at room temperature and submerged in 100 µl TMB 10% 3,3’, 5,5’-tetramethylbenzidine (SouthernBiotech) in DMSO (Sigma-Aldrich) diluted 1:100 in phosphate-citric buffer pH 5.0 and mixed with 30% H_2_O_2_ (Hedinger). The reaction was stopped after 5 min with 1 M sulfuric acid and the absorbance was read immediately at 450 nm (Biotek; µQuant Microplate Spectrophotometer). The IgG concentration (µg/ml) was calculated based on the standard curve.

#### White blood cell (WBC) counts

Blood smears were stained with May-Gruenwald’s solution (#T863.2, Carl Roth GmbH) and Giemsa (#T862.1, Carl Roth GmbH). The total WBC count for each individual was obtained by counting the number of WBCs in ten randomly selected microscope fields under 100x magnification, as detailed in ref. ^60^. Differential WBC counts were implemented by counting the relative numbers of neutrophils, basophils, eosinophils, monocytes, and lymphocytes among 100 leukocytes. Sixteen out of the 350 analyzed slides had a total WBC count of zero and the basophil count was zero for 224 of the slides. We therefore used the ratio of innate to adaptive white blood cells (i.e., the differential count of neutrophils + basophils + eosinophils + monocytes/lymphocytes) for further downstream analyses. This also equates to the ratio of myeloid to lymphoid cells and acts as a non-specific indicator of acute inflammation. To assess the reliability of these data, we then repeated differential WBC counts for a random 5% of slides and calculated the interclass correlation coefficient (ICC) using the R package psych version 2.2.9^61^. All of the ICC values were above 0.5, indicative of moderate to excellent repeatability (ICC (3, *k*) for neutrophils = 0.91, basophils = 0.57, eosinophils = 0.69, monocytes = 0.60, and lymphocytes = 0.84)^62^. Finally, we assessed the degree of association between the neutrophil-to-lymphocyte ratio, a frequently used marker of stress in vertebrates, and salivary baseline cortisol concentration per individual with a Pearson’s *r* using the R package stats version 4.2.1. Repeated measurements were taken into consideration by calculating the correlation coefficient of the individual means^63^.

#### dROM test

ROMs (primary products of oxidative damage) were measured in plasma using the d-ROMs test (Diacron International, Italy)^8,64^. We pipetted 4 µl of the sample plasma, reference standards (0.225 to 1.8 mM H_2_O_2_ equivalents), blanks, and quality controls in duplicate into 96-well plates. We added to each well 200 µl of a solution containing acetic acid/sodium acetate buffer (0.01 M, pH 4.8) and the chromogen N,N-diethyl-p-phenylenediamine (ratio 100:1). The ROMs that occur in the plasma react with the chromogen, generating a pink color whose intensity is proportional to the concentration. After an incubation at 37 °C for 75 min, the optical density was measured at 505 nm and the resulting values were expressed as mM of H_2_O_2_ equivalents.

#### OXY-adsorbent test

The non-enzymatic antioxidant capacity of the plasma was measured using the OXY-adsorbent test (Diacron International, Italy)^8,64^. This assay quantifies the in vitro reaction between non-enzymatic antioxidants (e.g., protein thiols, vitamins C and E) and hypochlorous acid (HOCl; pro-oxidant generated endogenously by vertebrates). All plasma samples, reference standards (340 mM), and quality controls were initially diluted 1:100 with distilled water. We then pipetted 5 µl of each one in duplicate into 96-well plates and immediately added 200 µl of HOCl. After incubation at 37 °C for 10 min, we added 2 µl of N,N-diethyl-p-phenylenediamine to each well and mixed the solution. The N,N-diethyl-p-phenylenediamine reacts with the HOCl that did not react with the plasma antioxidants, generating a pink color. We therefore measured the optical density at 505 nm and expressed the resulting OXY values as mM of HOCl neutralized.

#### Ransel test

We measured the activity of glutathione peroxidase (GPx) in plasma using the Ransel test (Randox Labs, UK). This assay quantifies the GPx relying on its catalytic activity of the oxidation of glutathione by cumune hydroperoxide. In the presence of glutathione reductase and NADPH, the oxidized glutathione is converted to the reduced form with a concomitant oxidation of NADPH, resulting in a decrease in absorbance at 340 nm with time. The kinetic reaction was followed for 3 min and a blank reaction was subtracted from the sample absorbance. The activity of GPx was expressed as units/l.

#### Ransod test

We measured the activity of superoxide dismutase (SOD) in plasma using the Ransod test (Randox Labs, UK). This assay employs xanthine and xanthine oxidase to generate superoxide radicals, which react with 2-(4-iodophenyl)-3-(4-nitrophenol)-5-phenyltetrazolium chloride (INT) to form a red formazan dye; SOD is measured by the degree of inhibition of this reaction due to its role in the dismutation of superoxide radicals. SOD activity was quantified using a calibration curve run for each assay. The activity of SOD was expressed as units/ml, where one unit of SOD causes a 50% inhibition.

### Development of immune and oxidative status markers over time

To investigate whether immune and oxidative status marker concentrations changed in pups and mothers during the course the study, we respectively compared group means at birth and molt. We then compared the concentration of each marker in pups relative to mothers at the two measured time points to determine whether pup concentrations had adjusted to adult levels by the time they began molting. We compared group means using ANOVA and Tukey’s HSD post-hoc tests in the R package multcomp version 1.4-20^65^. Cohen’s d measure of effect size was calculated using the R package rstatix version 0.7.0^66^. As proposed by ref. ^67^, |0.2| is considered a small, |0.5| a moderate, and |0.8| a large effect size.

### Statistics and reproducibility

Generalized linear mixed models were fitted in a Bayesian framework using Markov chain Monte Carlo methods in the R package MCMCglmm version 2.34^68,69^. We included season (food availability), colony (density), body condition, baseline cortisol, and sex as predictor variables of the immune markers (BKA (*E. coli* and *S. aureus*), hemagglutination, hemolysis, lysozyme, haptoglobin, neopterin, IgG, and the innate/adaptive WBC ratio). The nine immune markers were then independently fitted as predictor variables of the four oxidative status markers OXY, dROM, GPx, and SOD. The hypothesized causal relationships among the predictor and response variables are depicted in Fig. 1c as a directed acyclic graph (DAG). Sets of covariates for the unbiased estimation of direct and total causal effects^70^ were checked using the R package dagitty version 0.3.2^71^.

#### Justification for the DAG casual structure

We hypothesized direct effects of season on density, body condition, and baseline cortisol. Yearly environmental changes on Bird Island are the main driver of variability in food availability, with the first year of this study being among the worst on record in terms of food abundance. Consequently, significantly fewer individuals came ashore to breed and population densities were overall lower in 2019 compared with 2020^12^. Our previous research has further shown that both body condition^12^ and salivary cortisol levels^49^ vary by year. Finally, lower food availability is expected to limit an individual’s ability to mount an immune response^4^, an effect that is likely to be stronger in pups, which have to invest in other energetically demanding processes such as growth.

We hypothesized direct effects of colony on body condition, baseline cortisol, and immune markers. We know from previous studies of the same population that body condition^12^ and hair cortisol concentrations^13^ differ significantly between the two breeding colonies. This variability has been attributed to contrasting population densities, with FWB having significantly fewer breeding females per square meter^13^ and a lower modal local density of pups throughout the breeding season^12^. In general, the transmission of parasites and pathogens is also density dependent and consequently population density tends to correlate positively with immune cell profiles and the strength of immune responses^72–74^.

We hypothesized direct effects of three additional variables on immune marker levels: (i) Sex. Immune responses often differ between the sexes, although the direction of immunocompetence can depend on a number of factors, such as sex-specific growth patterns and behaviors^33^. Sex has also been shown to influence cortisol levels in Antarctic fur seal pups^49^. (ii) Cortisol. Glucocorticoids may mediate immune responses because elevated levels of the stress hormone have been strongly linked to immune defense suppression^35^. Reverse causality has, however, also been suggested, whereby parasite infection may elicit an increase in plasma cortisol^75^. (iii) Body condition. Studies of wild populations provide strong evidence for a two-way relationship between body condition and immunity^37^. In particular, poor condition may predispose an individual to infection, which will further reduce condition.

Immune markers are included in our DAG as response variables, and predictors of oxidative status marker concentrations. Assuming limited resources, there is a predicted trade-off between investment in immune function and antioxidant defense^76^. Furthermore, increased innate immune activity may necessitate the downregulation of antioxidant enzymes, which would otherwise minimize inflammation and remove reactive oxygen species purposefully generated to kill bacteria^8^.

#### Random effects structure

All of our models included individual identity (ID) as a random variable to control for repeated measures. We also considered the possibility that the time of sampling could contribute towards some of the variation in our response variables given that components of the immune system are known to oscillate along an active/rest cycle^77^. Previous research has shown that Antarctic fur seal pups are diurnal^78^ but less is known about adult female activity patterns. We therefore fitted linear models to assess the proportion of variability in immune and oxidative status marker concentrations explained by the hour of sample collection. Models were fitted using the R package lmer version 1.1-30^79^. None of the markers crossed a pre-defined threshold R-squared value of 5%, with the average proportion of variance in the dependent variable explained by the hour of sampling being 0.48% (Supplementary Table S3). We therefore elected not to include the time of sampling as a random effect in the models.

#### Model parameters

All immune and oxidative status markers were zero mean and unit standardized. Baseline cortisol, a continuous predictor variable, was measured in ng/ml. Binary predictor variables were coded as such (i.e., 0/1), with 2019 as the reference category for season (a proxy of food availability), FWB as the reference category for colony (a proxy of density), and female as the reference category for sex. Mothers and pups were analyzed separately. Sex was not included as a predictor variable in models for the mothers.

We fitted models with a Gaussian distribution. For the R structure in our model, we kept the flat, default prior for the mean (−10^6^, 10^6^) and specified a weak prior variance (V = 1, nu = 0.002). For the G structure (i.e., random effects variance components), we specified a proper Cauchy prior (V = 2, nu = 1). Pilot runs (thinning = 10, burn-in samples = 3000, number of iterations = 13,000) indicated autocorrelation for some variance components. We therefore increased the thinning interval, burn-in, and number of iterations (x 100) to guarantee the mean and variance effective sample sizes were least 600, but ideally ≥1000. We assessed convergence of the chains visually and ensured that autocorrelation was less than 0.1. Our final check was to simulate new data given the model parameter values (variance/co-variance structures) and plot them against our real data to ensure they overlapped.

#### Model outputs

For the binary response variables, the standardized mean difference was used as an index of effect size^80^. For the continuous variables, the posterior was the concentration of probability around the estimate of the mean, such that positive values are indicative of a positive relationship between the predictor and response. We considered a predictor variable to significantly correlate with the response when the *p*MCMC < 0.05, where *p*MCMC is equal to two times the smaller of the MCMC posterior probability estimates that the parameter value is above or below zero.

#### Repeated measures

Repeatability, i.e., individual consistency in immune marker levels over the 60 days from birth to the start of molt, was calculated using the full model according to ref. ^81^. Specifically, R = V_ID_/(V_ID_ + V_F +_ V_R_), where V_ID_ stands for between-individual variance (here, also random effect variance), V_F_ for the fixed effect variance, and V_R_ for the residual variance. For comparison, as recommended by ref. ^82^, repeatability was also calculated for the null model (i.e., without including the fixed effects), which provides an estimate of the variance observed in the raw data. A high repeatability (i.e., point estimates for R approaching 1) would indicate that individual immune marker levels differ minimally between sampling time points, suggesting comparable investment into immune parameters at birth and molt. Given that we collected two measurements per individual and have repeated measures from 67 pups and 80 mothers, the standard error of the within-subject standard deviation is 15%^83^.

We ran all analyses in R version 4.2.1^84^. The raw data and code can be found on Zenodo^85^.

### Reporting summary

Further information on research design is available in the Nature Portfolio Reporting Summary linked to this article.

### Supplementary information

Supplementary Information

Description of Additional Supplementary Files

Supplementary Data 1

Supplementary Data 2

Reporting Summary

## Data Availability

Raw data including the numerical source data underlying the graphs and charts shown in the manuscript can be found on Zenodo under 10.5281/zenodo.11208287.

## Code availability

We ran all analyses in R version 4.2.1. Code can be found on Zenodo under 10.5281/zenodo.11208287.
