# Supplementary material for: Life-history stage influences immune investment and oxidative stress in response to environmental heterogeneity in Antarctic fur seals
Source: Commun Biol. 2024 Jun 29;7:788. doi: 10.1038/s42003-024-06499-6 (PMC11217341; doi:10.1038/s42003-024-06499-6)
Supplement: Supplementary file 1 — Supplementary Information [file 42003_2024_6499_MOESM1_ESM.docx]

Supplementary Information

Life-history stage influences immune investment and oxidative stress in response to environmental heterogeneity in Antarctic fur seals

Rebecca Nagel, Katja Pohle, Lilla Jordán, Iva Tuponja, Claire Stainfield, Camille Toscani, Cameron Fox‑Clarke, David Costantini, Gábor Á. Czirják, Jaume Forcada, Joseph I. Hoffman

Supplementary Fig. S1.


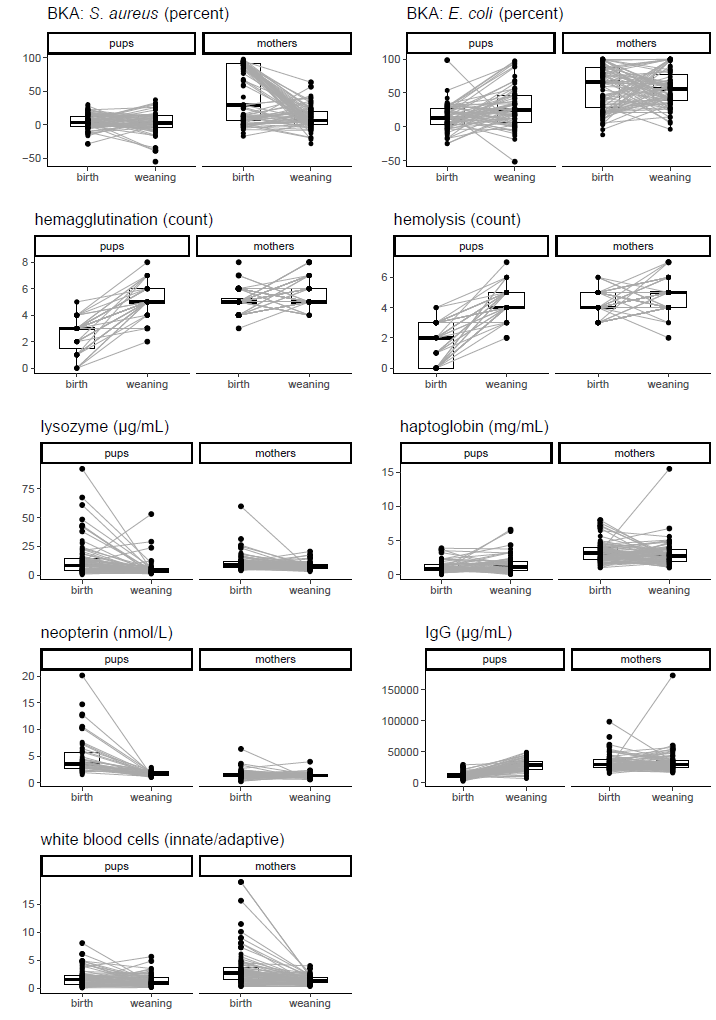


Immune response over time. Data is plotted using the ggpubr version 0.4.0 package in R ^1^. Boxes show median values ±75% percentiles with the vertical lines indicating 95% confidence intervals. Lines connect repeated measures taken within the same individual.

**Supplementary Fig. S2.**


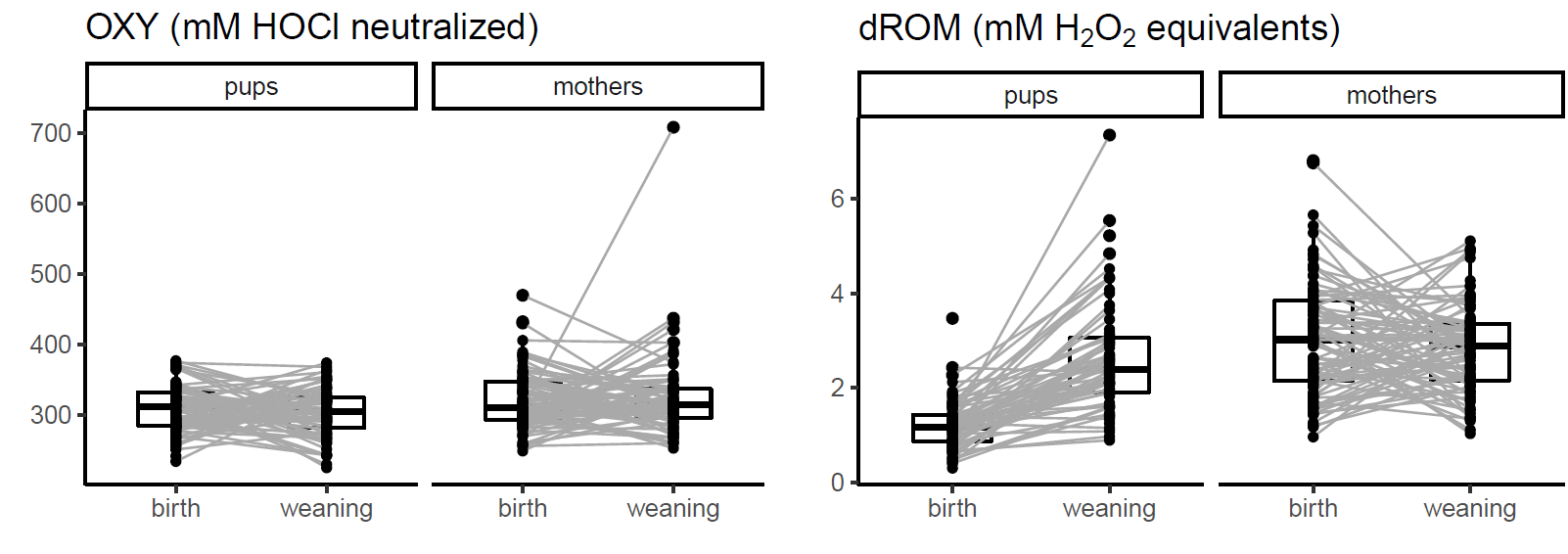


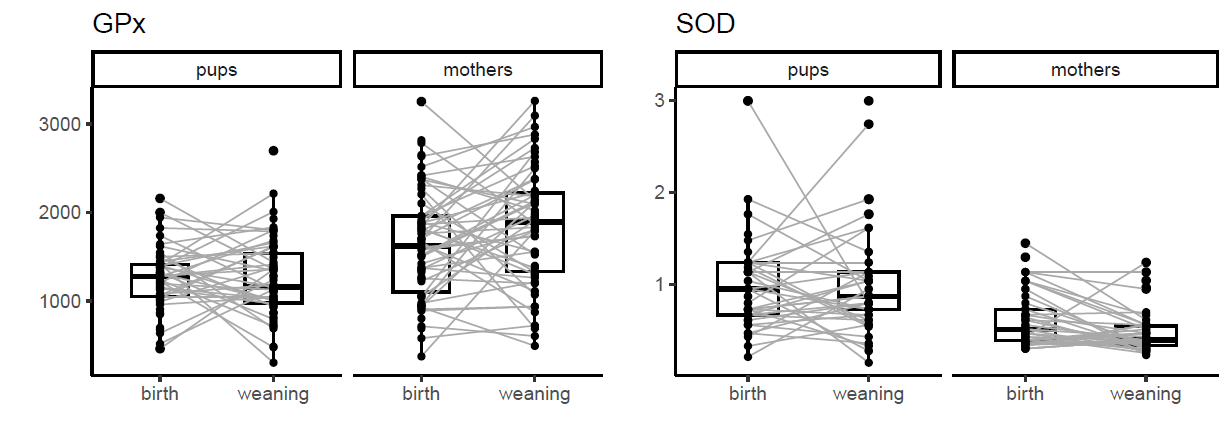


Oxidative stress response over time. Data is plotted using the ggpubr version 0.4.0 package in R ^1^. Boxes show median values ±75% percentiles with the vertical lines indicating 95% confidence intervals. Lines connect repeated measures taken within the same individual.

**Supplementary Fig. S3.**


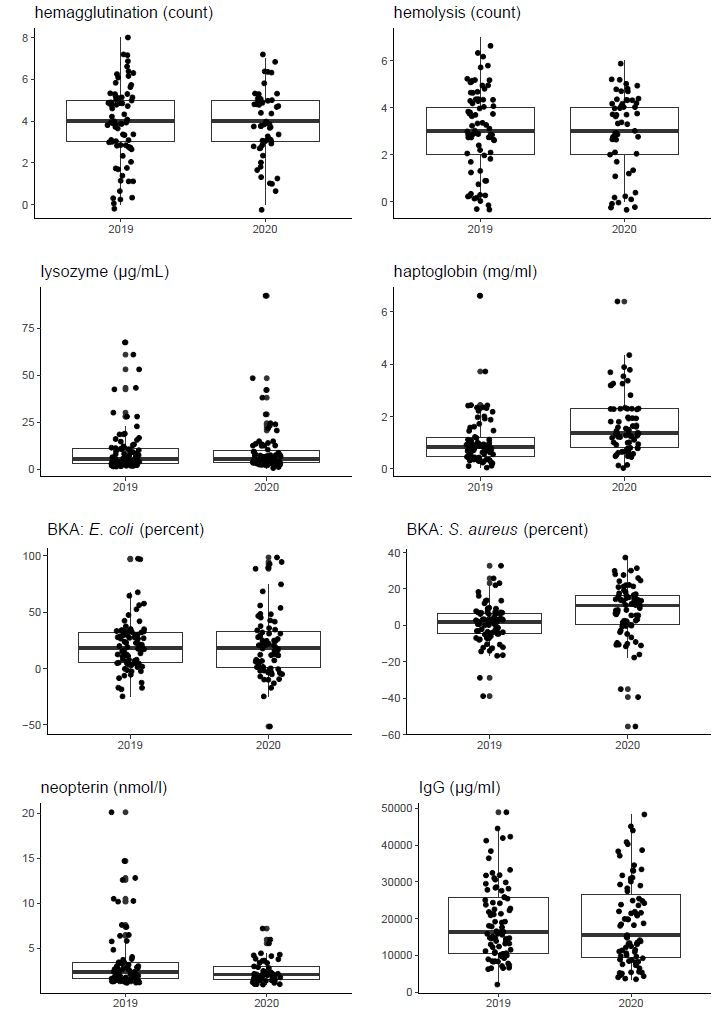


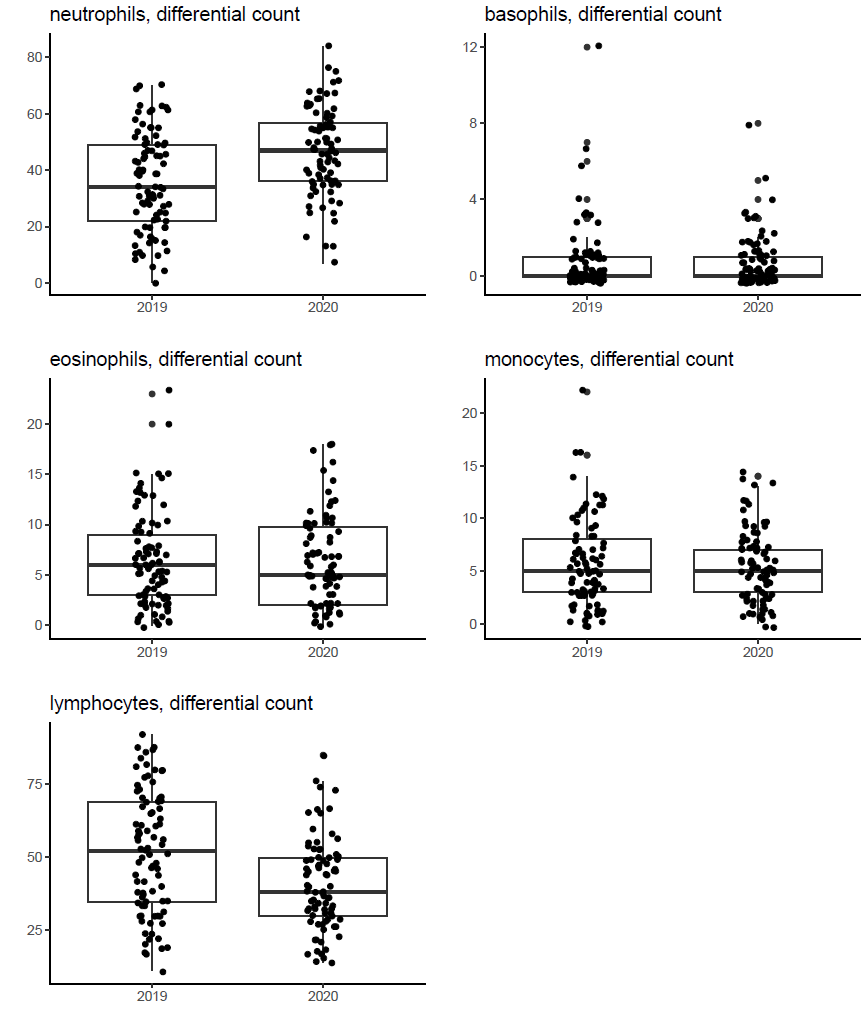
 Raw data for all immune markers measured in pups, delimited by season. Boxes show median values ±75% percentiles with the vertical lines indicating 95% confidence intervals.

**Supplementary Fig. S4.**


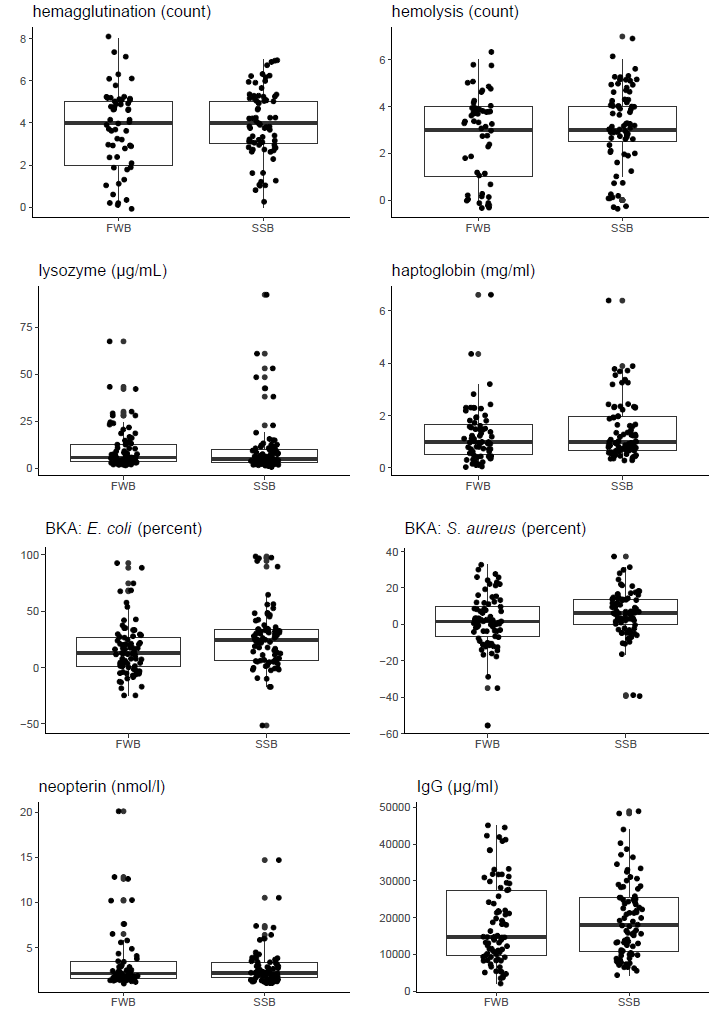


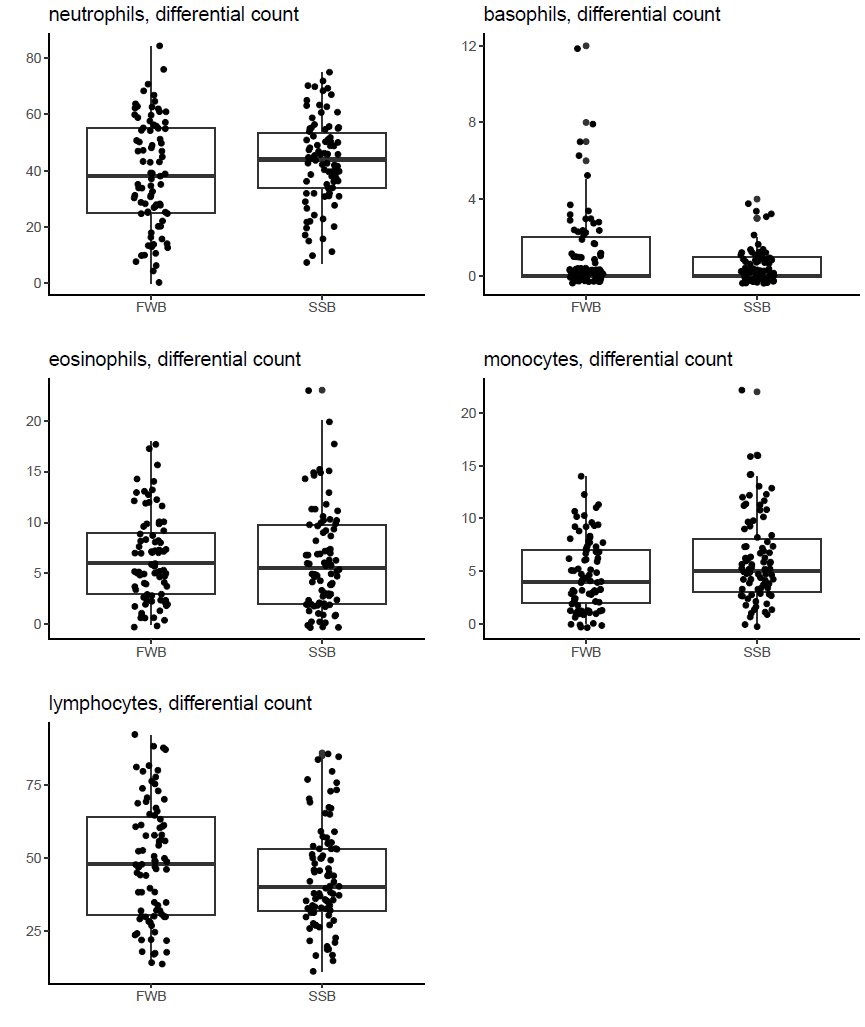
 Raw data for all immune markers measured in pups, delimited by colony. Boxes show median values ±75% percentiles with the vertical lines indicating 95% confidence intervals.

**Supplementary Fig. S5.**


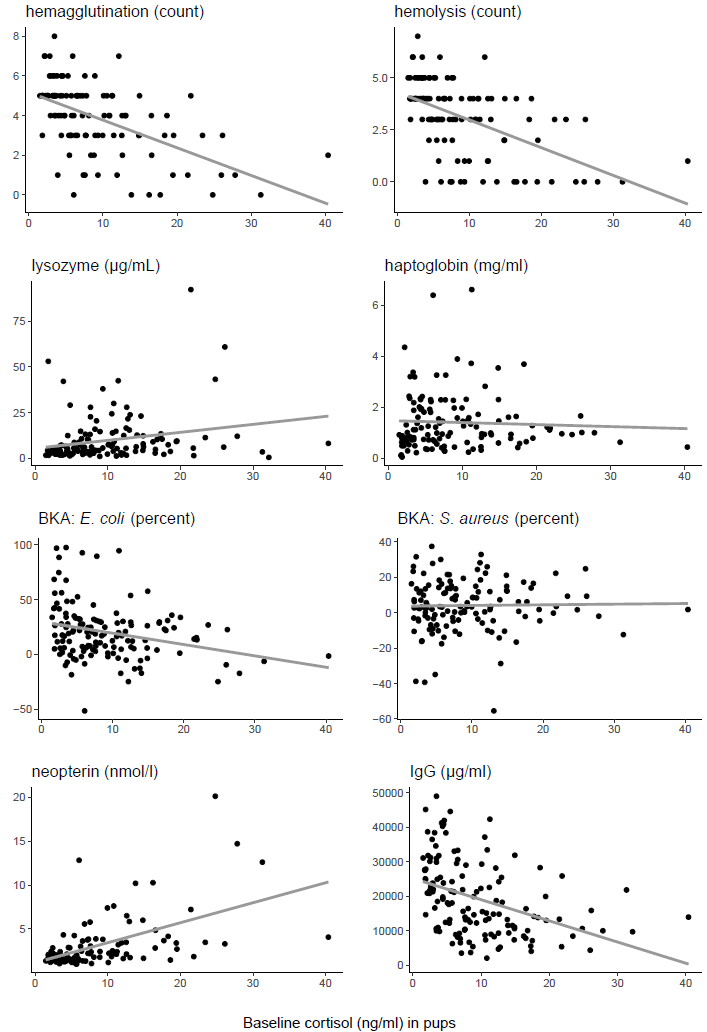


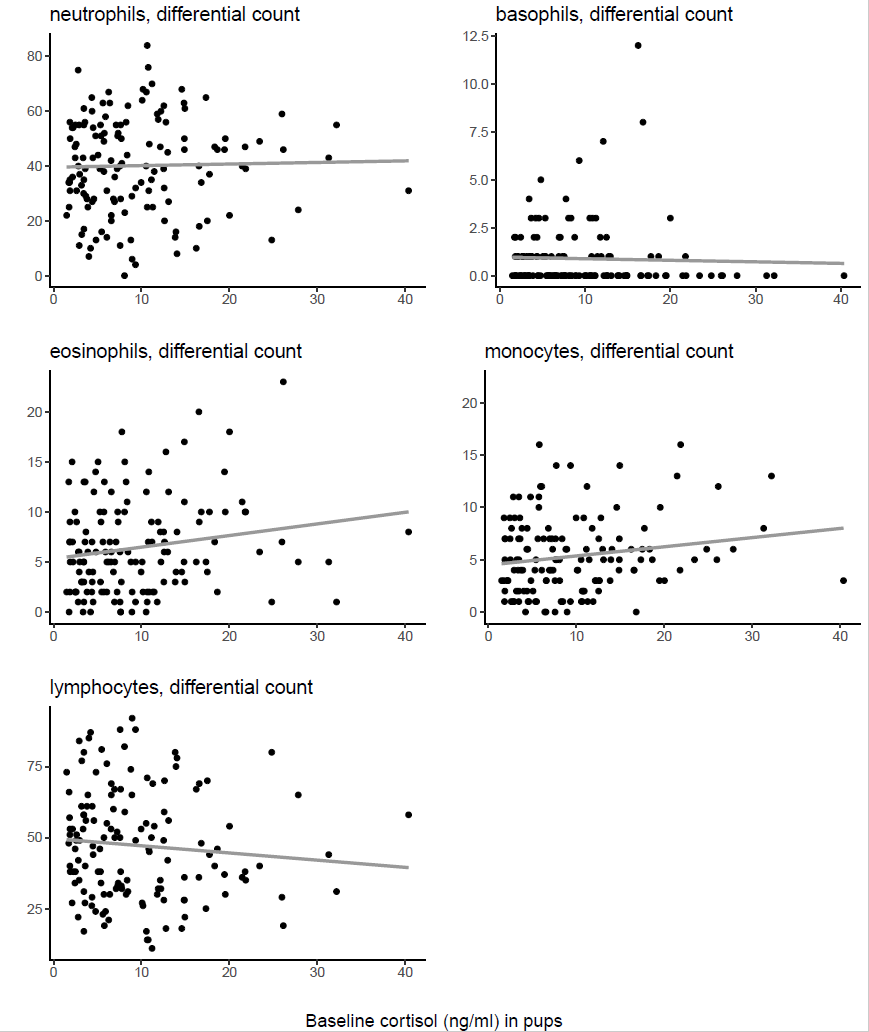
 Raw data for all immune markers measured in pups regressed against baseline cortisol. The line shows the linear regression between the predicted (cortisol) and response (immune marker) variable.

**Supplementary Fig. S6.**


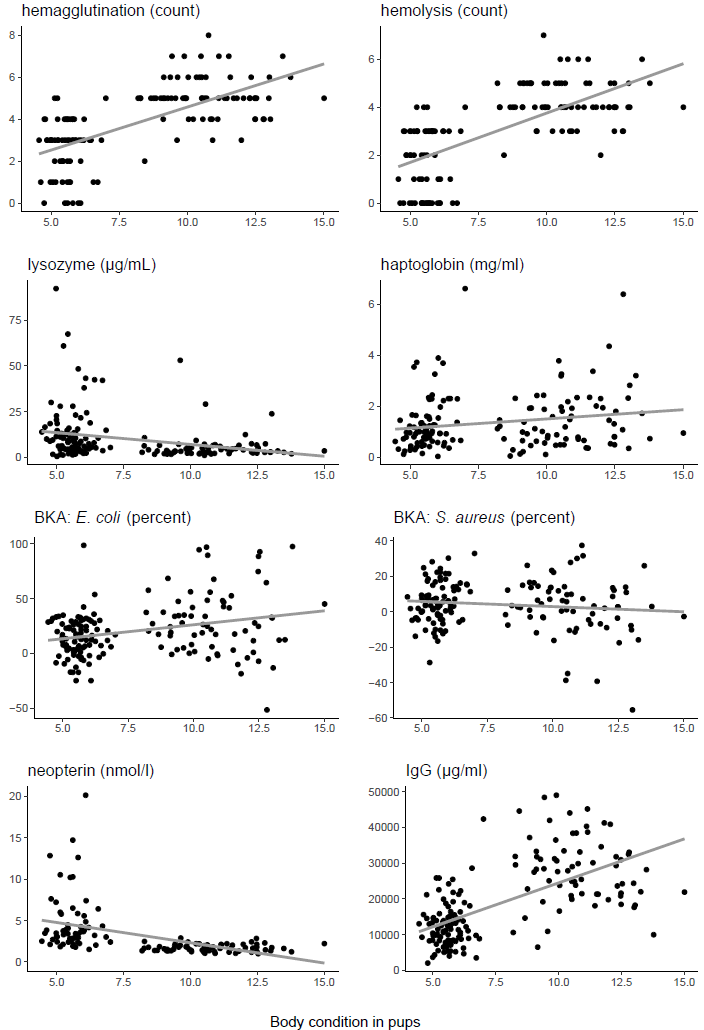


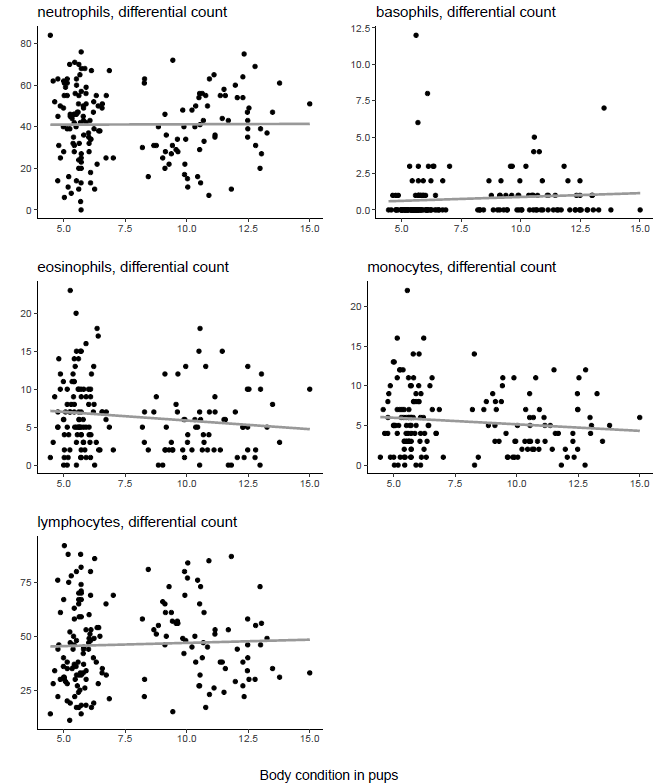


Raw data for all immune markers measured in pups regressed against body condition. The line shows the linear regression between the predicted (body condition) and response (immune marker) variable.

**Supplementary Fig. S7.**


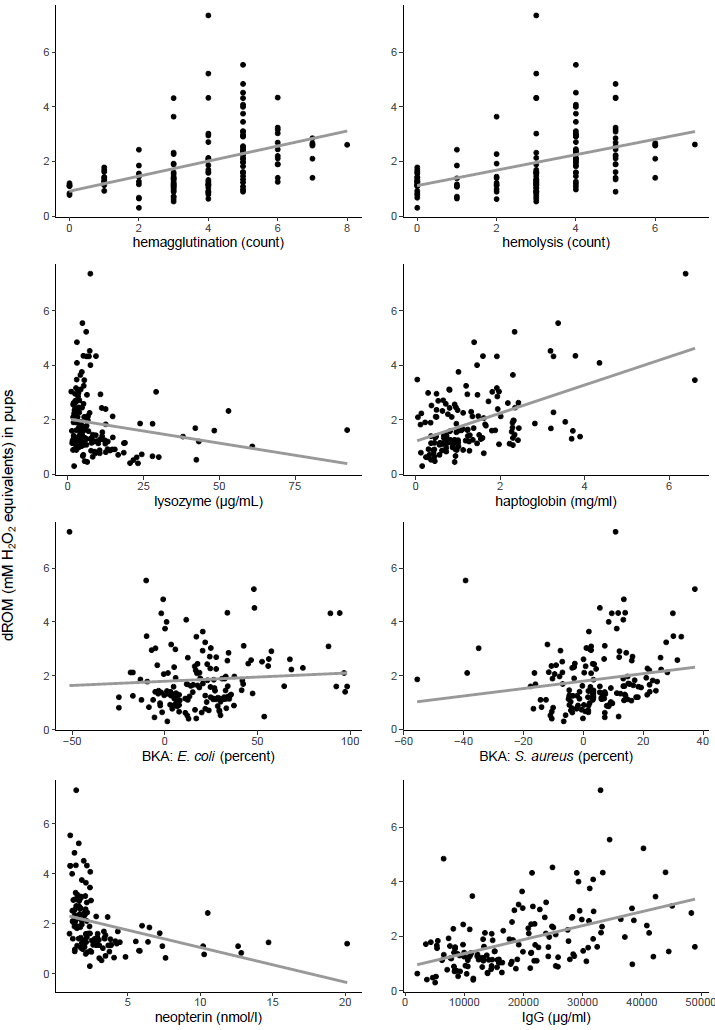


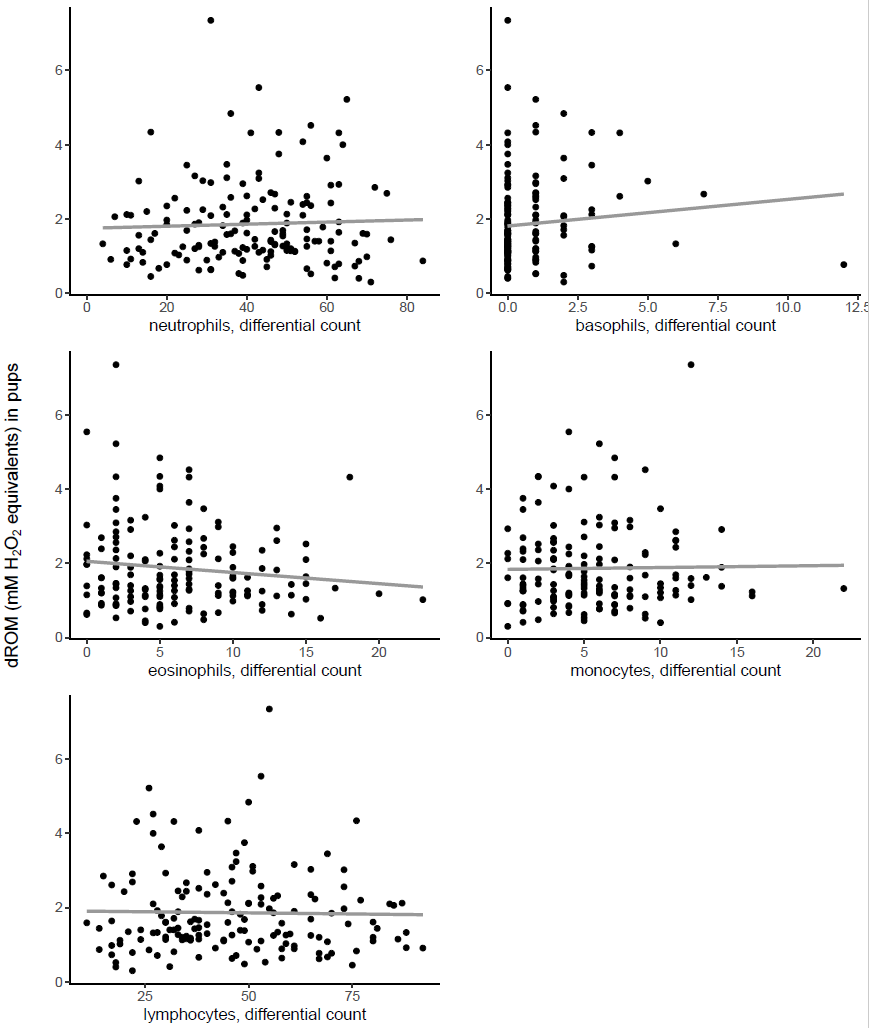


Raw data for dROM, regressed against all immune markers measured in pups. The line shows the linear regression between the predicted (immune marker) and response (dROM) variable.

**Supplementary Fig. S8.**


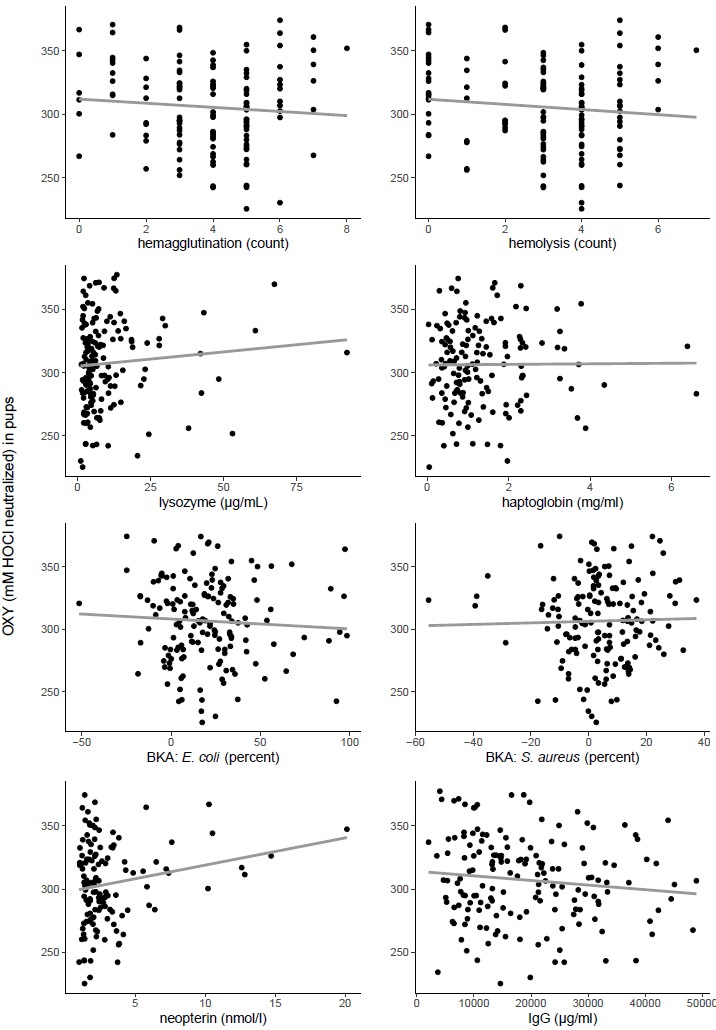


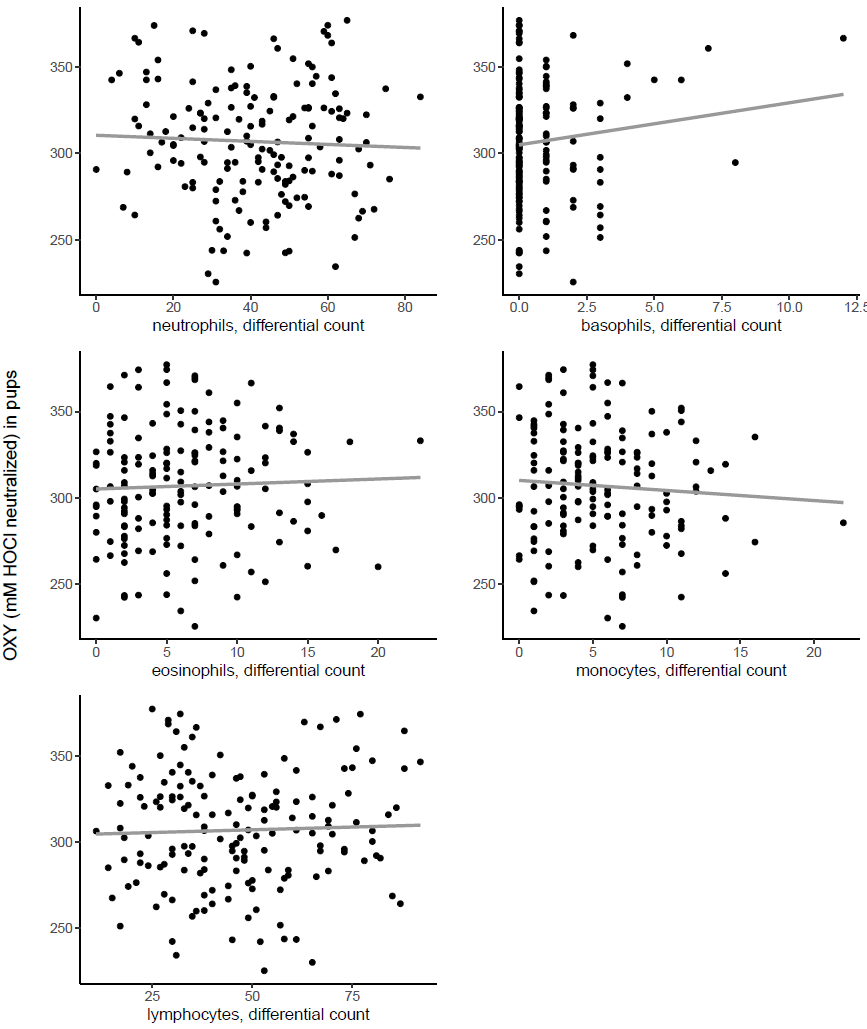


Raw data for OXY, regressed against all immune markers measured in pups. The line shows the linear regression between the predicted (immune marker) and response (OXY) variable.

**Supplementary Fig. S9.**


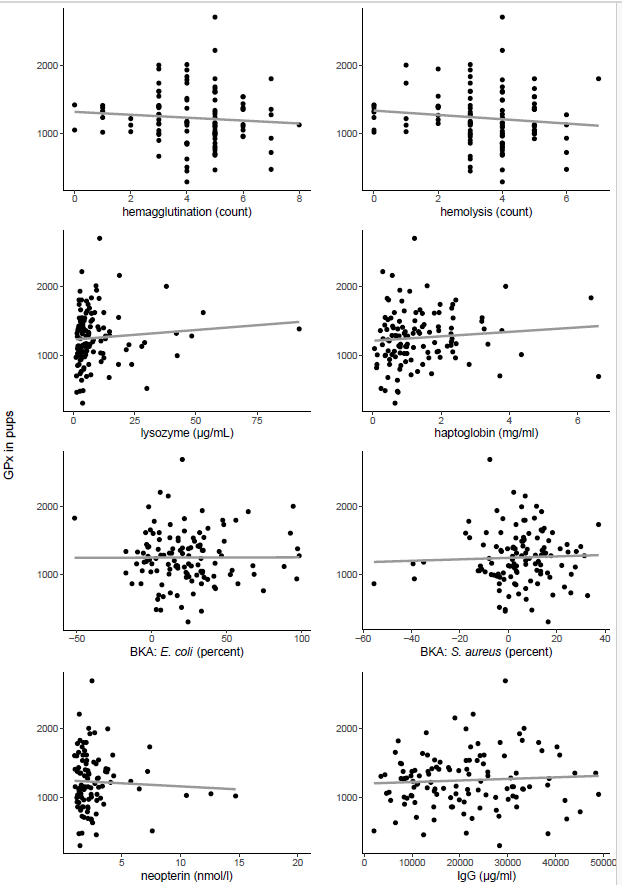

Raw data for GPx, regressed against all immune markers measured in pups. The line shows the linear regression between the predicted (immune marker) and response (GPx) variable.

**Supplementary Fig. S10.**

Raw data for SOD, regressed against all immune markers measured in pups. The line shows the linear regression between the predicted (immune marker) and response (SOD) variable.

**Supplementary Fig. S11.**


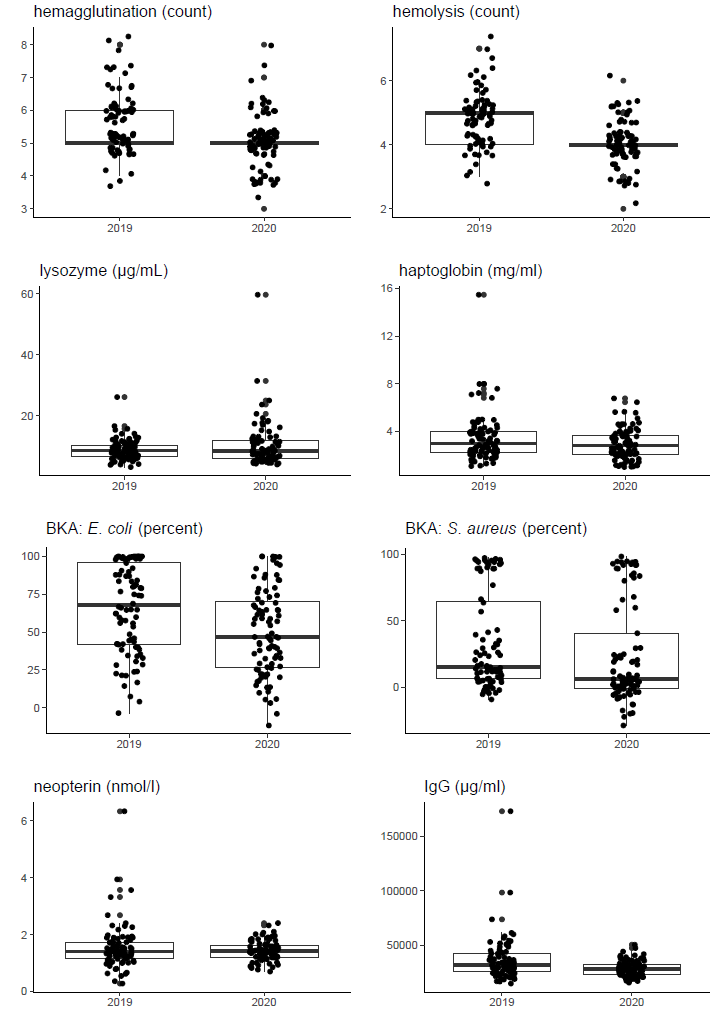


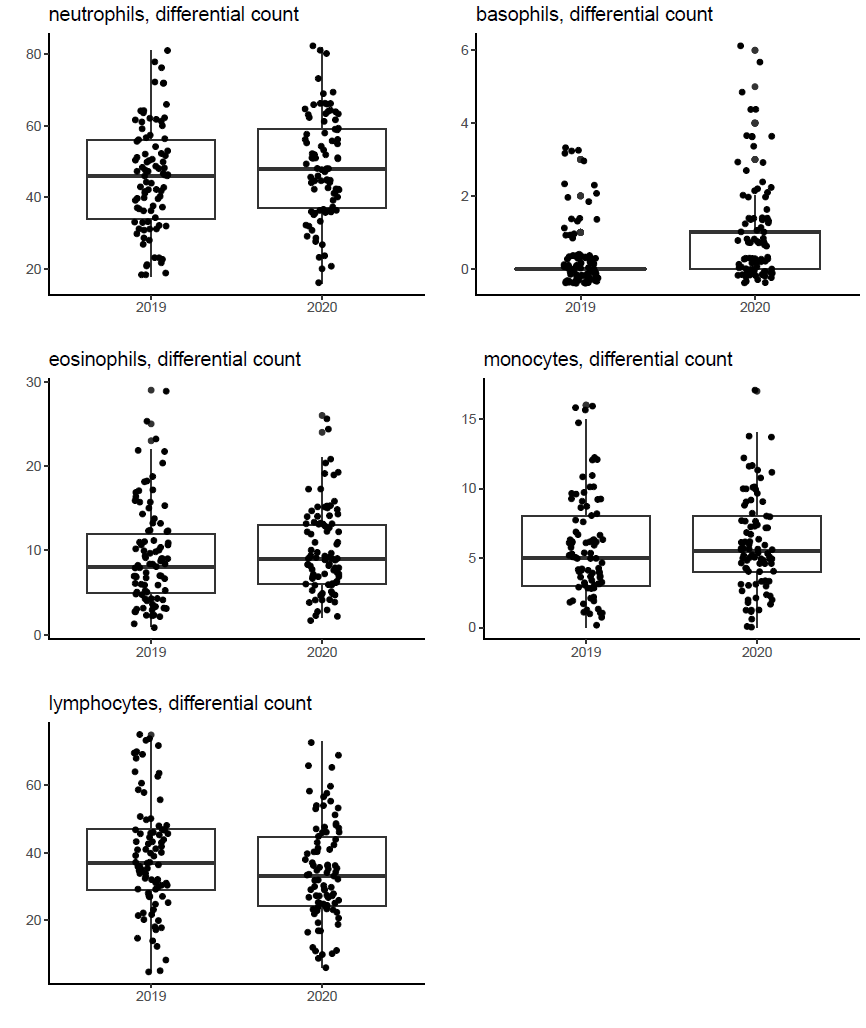
 Raw data for all immune markers measured in mothers, delimited by season. Boxes show median values ±75% percentiles with the vertical lines indicating 95% confidence intervals.

**Supplementary Fig. S12.**


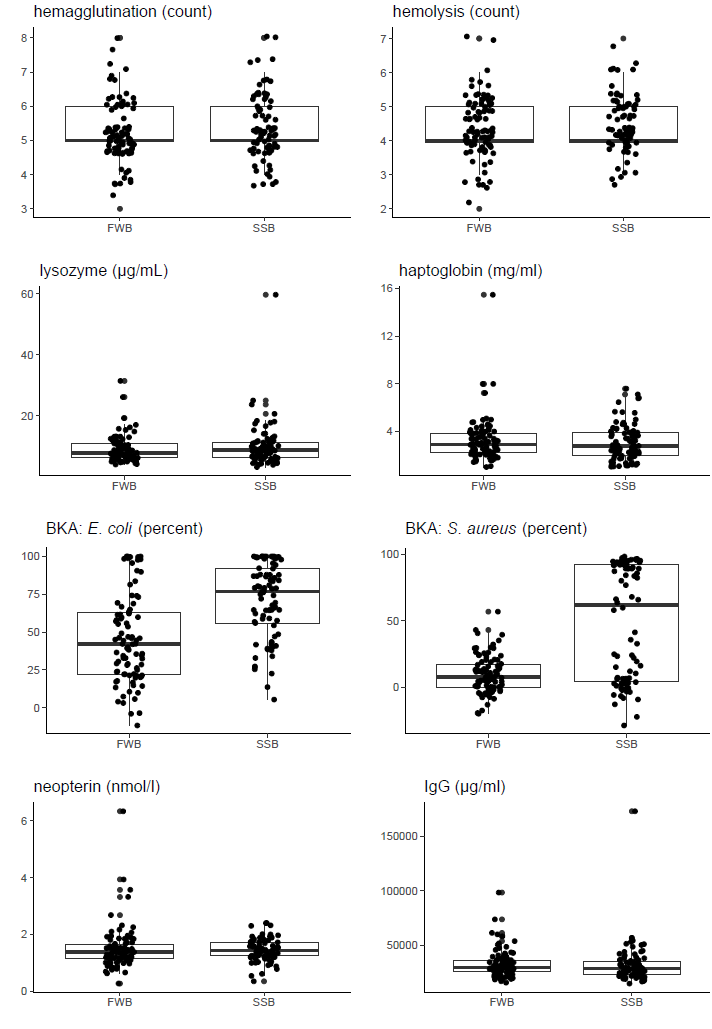


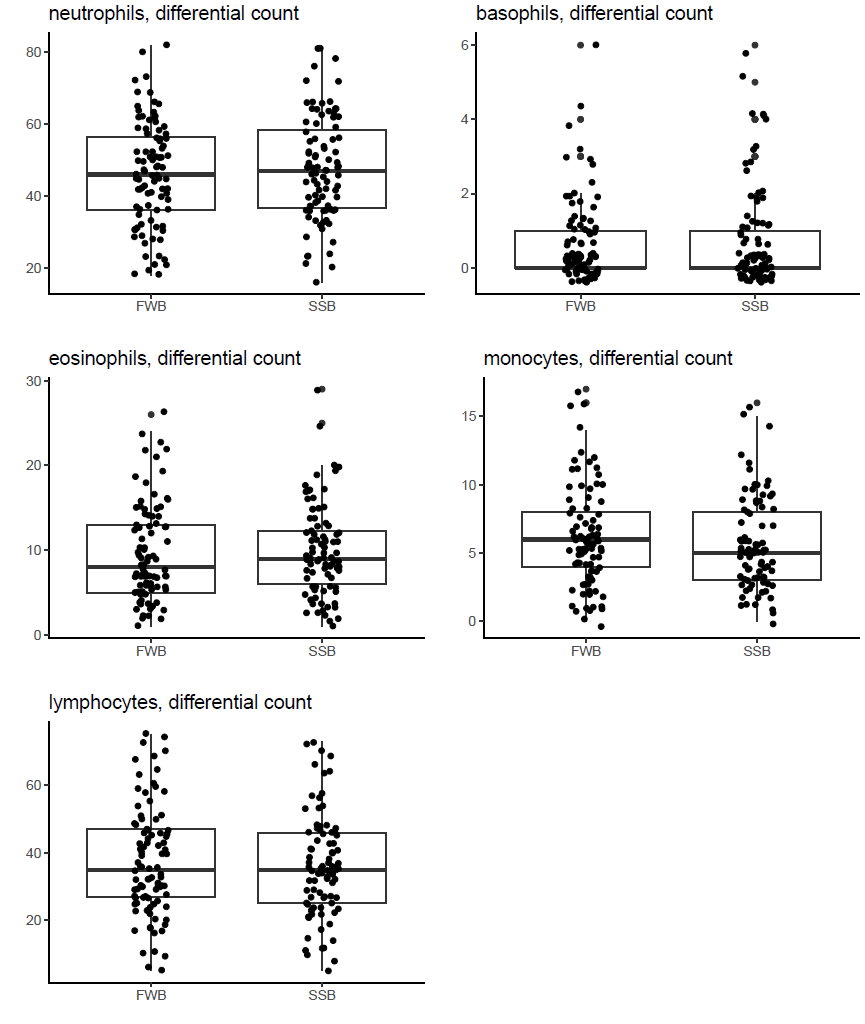


Raw data for all immune markers measured in mothers, delimited by colony. Boxes show median values ±75% percentiles with the vertical lines indicating 95% confidence intervals.

**Supplementary Fig. S13.**


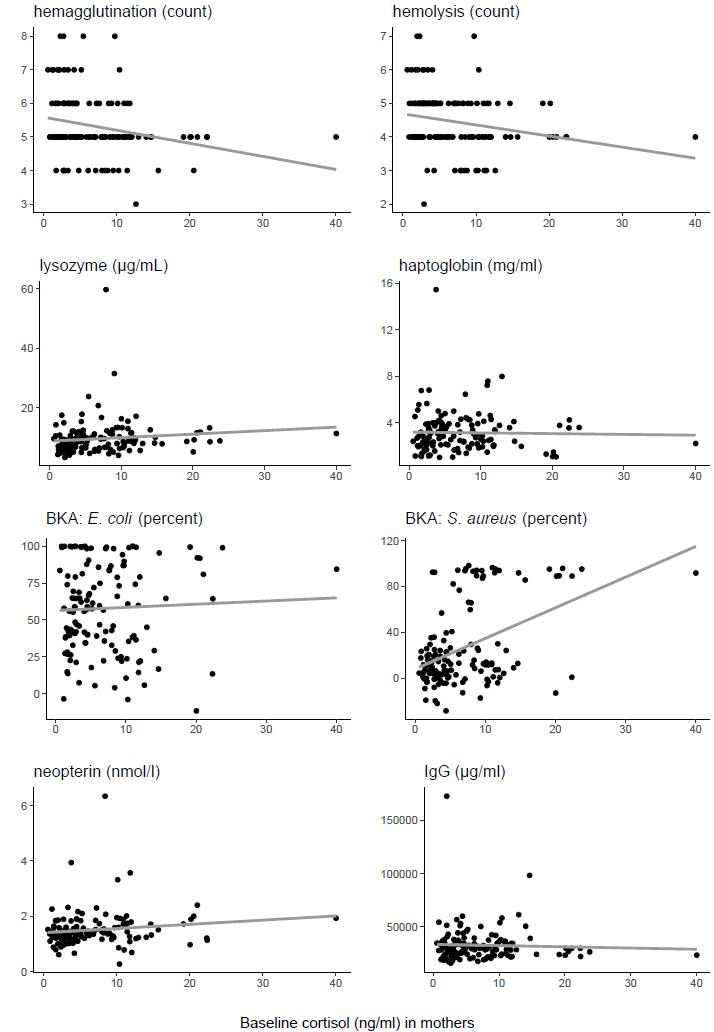


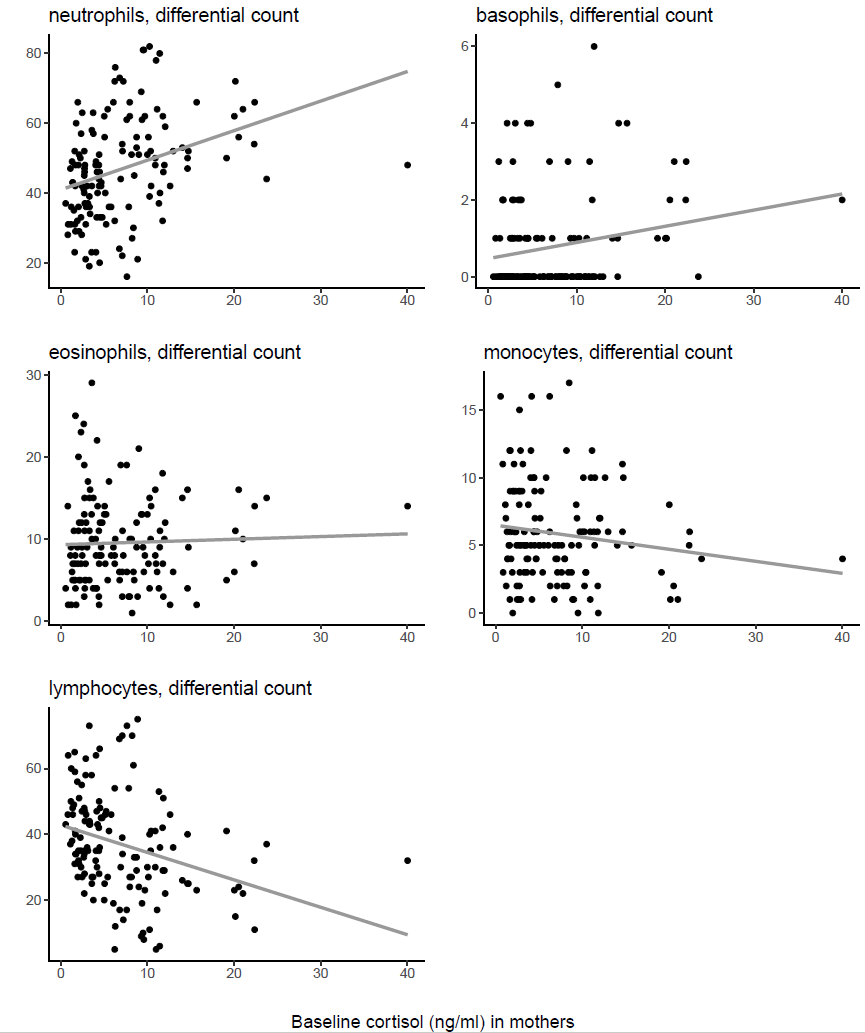


Raw data for all immune markers measured in mothers regressed against baseline cortisol. The line shows the linear regression between the predicted (cortisol) and response (immune marker) variable.

**Supplementary Fig. S14.**


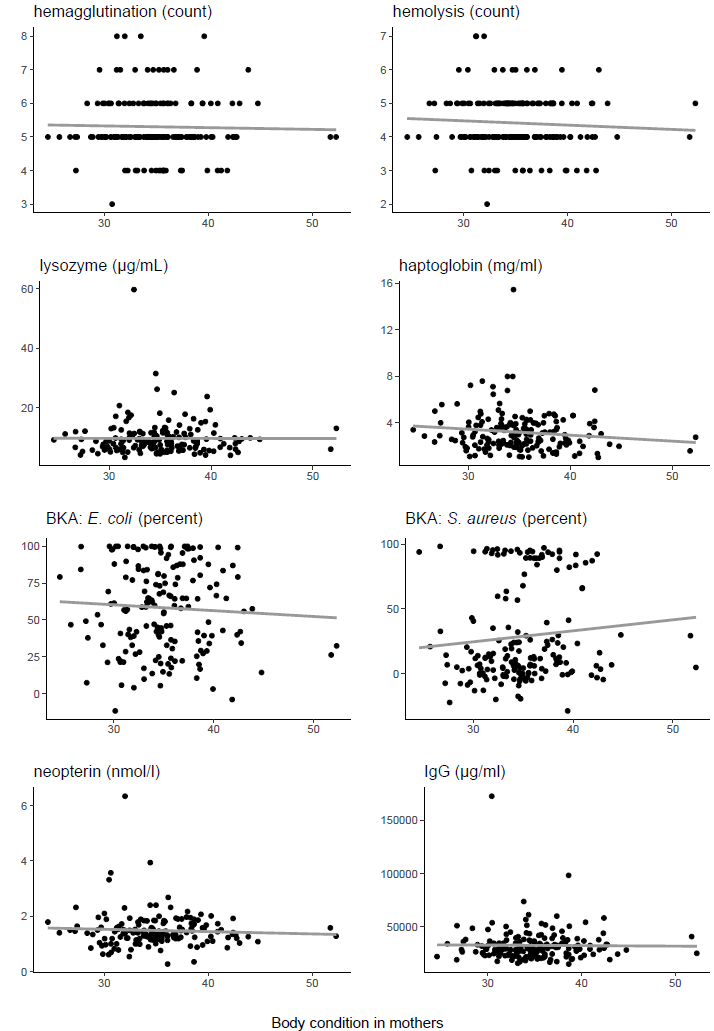


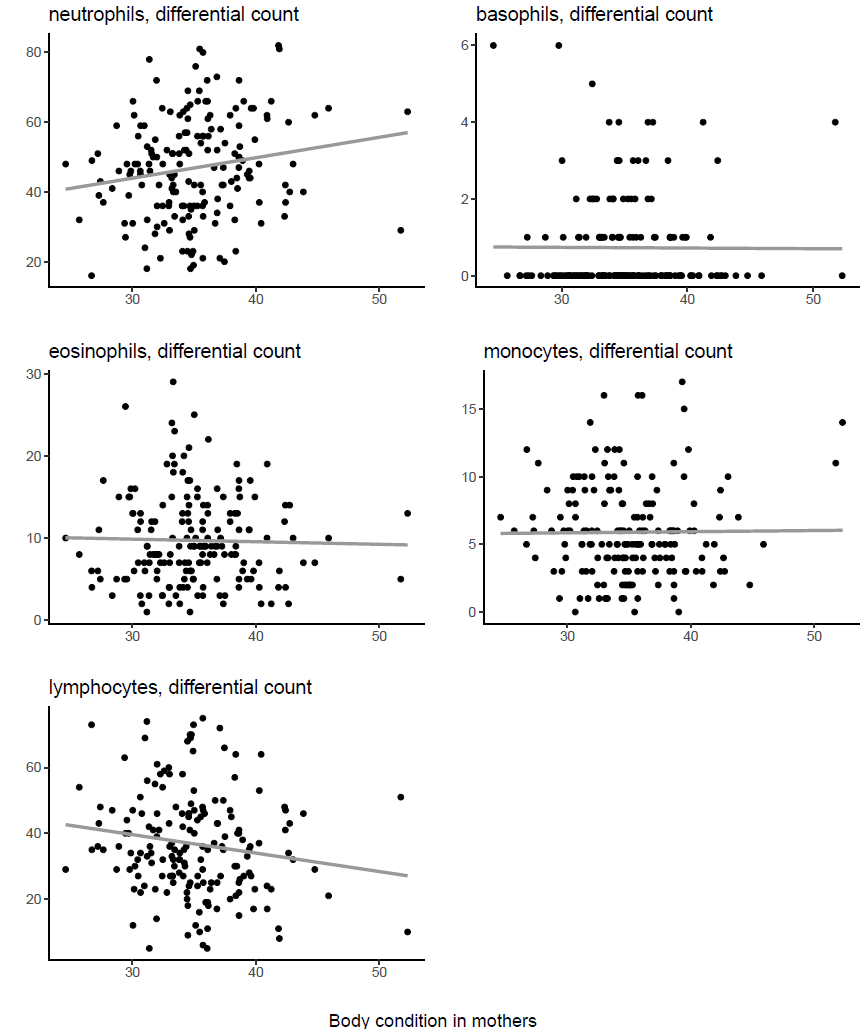


Raw data for all immune markers measured in mothers regressed against body condition. The line shows the linear regression between the predicted (body condition) and response (immune marker) variable.

**Supplementary Fig. S15.**


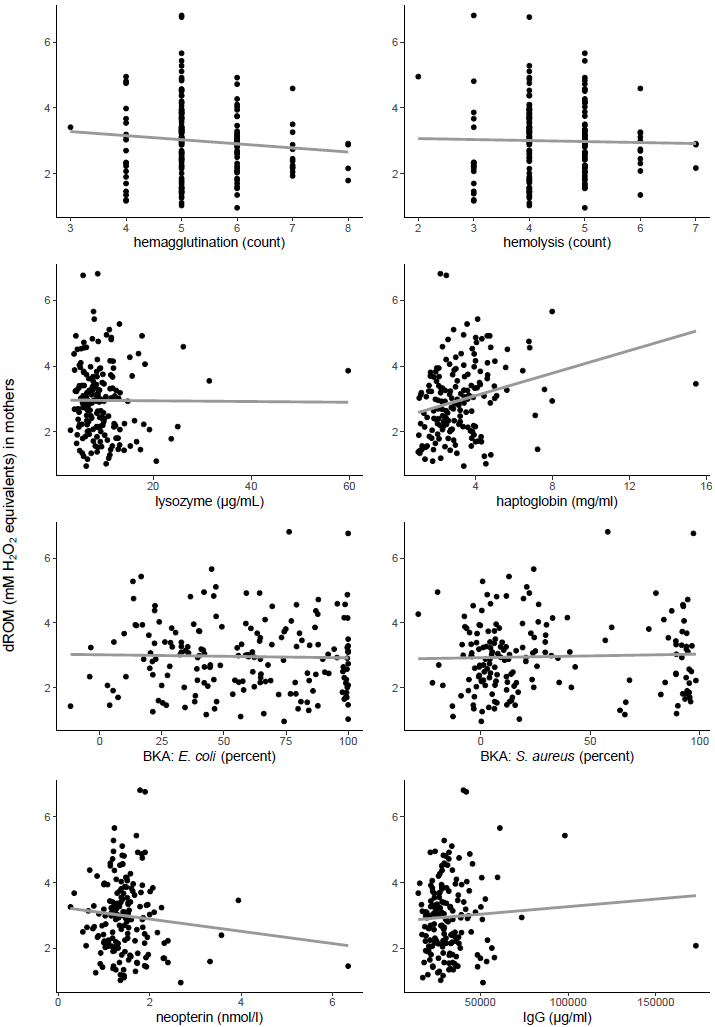


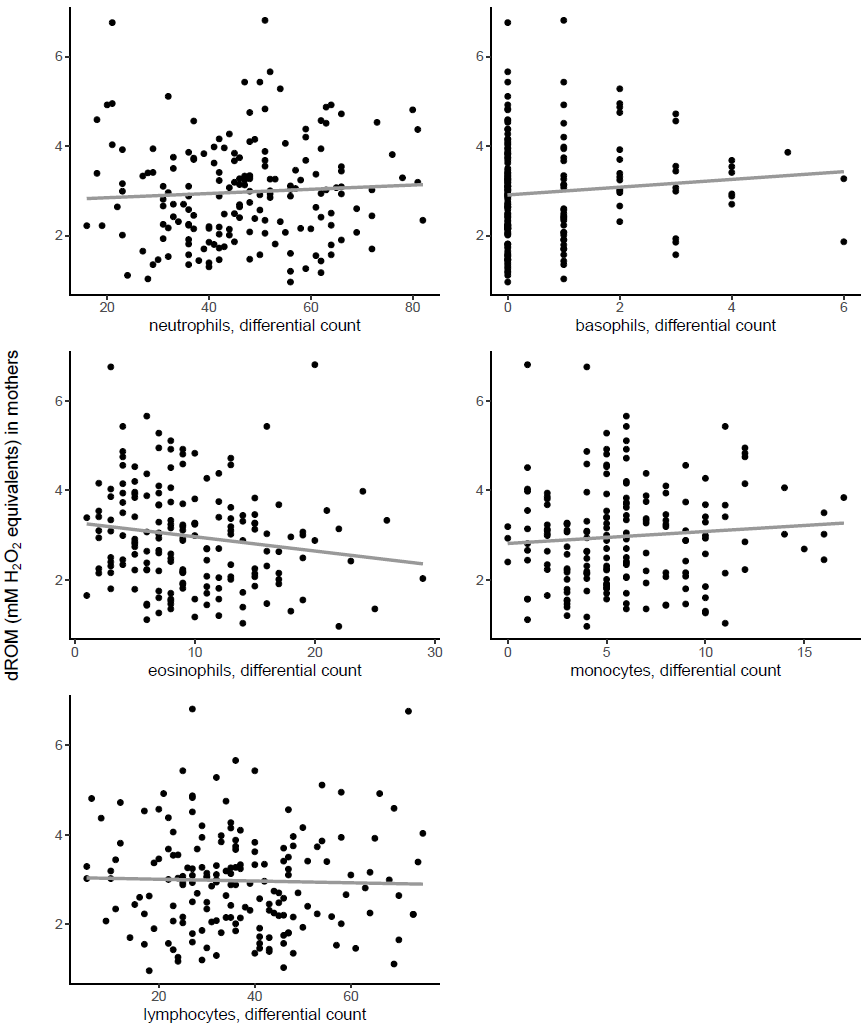


Raw data for dROM, regressed against all immune markers measured in mothers. The line shows the linear regression between the predicted (immune marker) and response (dROM) variable.

**Supplementary Fig. S16.**


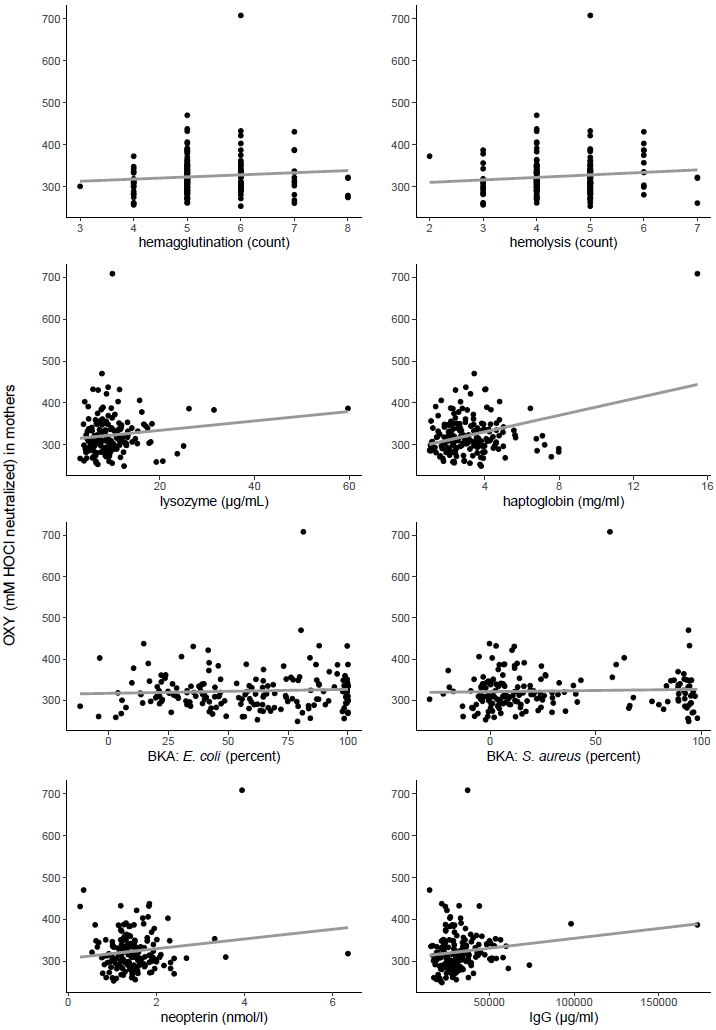


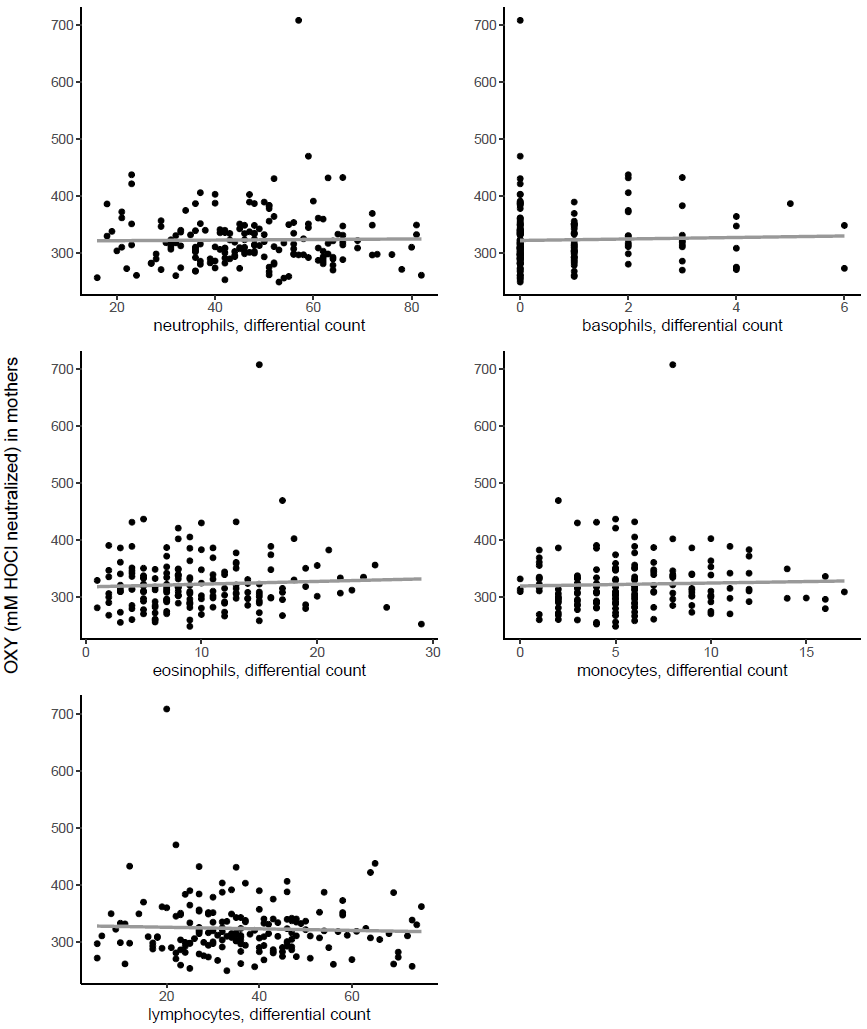


Raw data for OXY, regressed against all immune markers measured in mothers. The line shows the linear regression between the predicted (immune marker) and response (OXY) variable.

**Supplementary Fig. S17.**

Raw data for GPx, regressed against all immune markers measured in mothers. The line shows the linear regression between the predicted (immune marker) and response (GPx) variable.

**Supplementary Fig. S18.**

Raw data for SOD, regressed against all immune markers measured in mothers. The line shows the linear regression between the predicted (immune marker) and response (SOD) variable.

**Supplementary Fig. S19.**


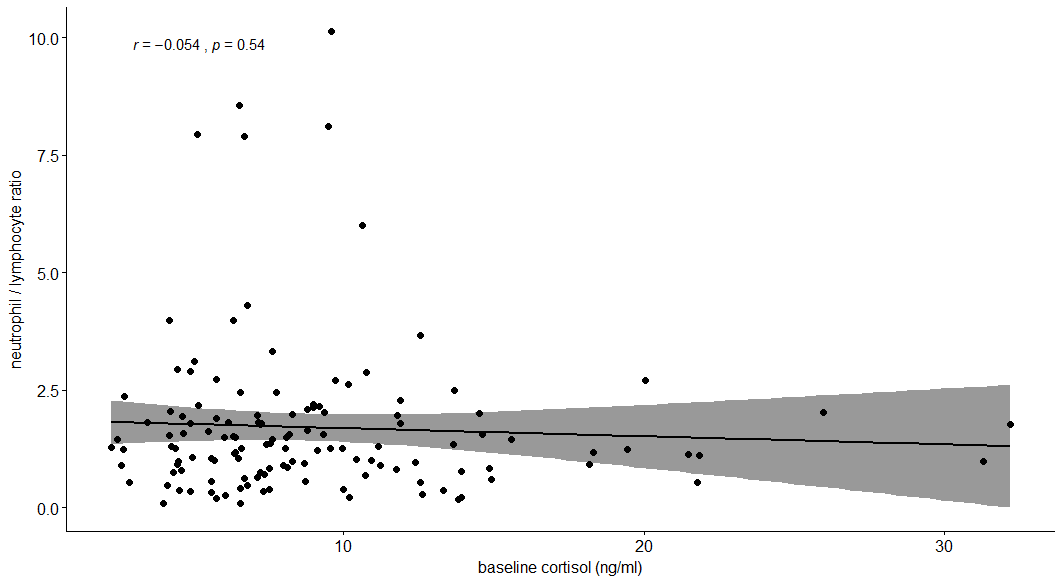


Pearson correlation coefficient denoting the degree of association between the neutrophil / lymphocyte ratio and baseline cortisol in n = 350 Antarctic fur seals (cor.test: *r* = -0.05, 95% CI = -0.22 – 0.12, *t* = -0.62, df = 130, *p* = 0.54). Repeated measurements were taken into consideration by calculating the correlation coefficient for the mean neutrophil / lymphocyte ratio and the mean baseline cortisol per individual ^2^. The plot was made using the R package ggpubr version 0.4.0 ^1^.

**Supplementary Table S1**. Summary statistics for focal Antarctic fur seals. (a) We collected data from 94 focal pups (by sex, 46 females and 48 males: by season, 51 from 2019 and 43 from 2020: by colony, 47 from FWB and 47 from SSB). (b) We collected data from 93 focal mothers (by season, 47 from 2019 and 46 from 2020: by colony, 48 from FWB and 45 from SSB).

| (a) pups at birth / weaning | Age (days) | Weight (kg) | Length (cm) | Body condition ^3^ | Baseline cortisol (ng/ml) |
| --- | --- | --- | --- | --- | --- |
| Min. | 0 / 45 | 3.5 / 5.8 | 53.0 / 65.5 | 4.4 / 7.0 | 3.9 / 1.5 |
| 1st Qu. | 0 / 55 | 5.1 / 9.5 | 63.0 / 76.5 | 5.2 / 9.5 | 8.1 / 2.8 |
| Median | 0 / 63 | 5.6 / 10.8 | 65.0 / 81.0 | 5.6 / 10.5 | 11.8 / 3.8 |
| Mean | 0 / 63 | 5.6 / 10.8 | 64.7 / 80.6 | 5.6 / 10.7 | 13.4 / 5.0 |
| 3rd Qu. | 0 / 70 | 6.2 / 12.4 | 67.0 / 85.0 | 5.9 / 12.0 | 16.7 / 6.0 |
| Max. | 0 / 90 | 7.8 / 16.8 | 74.5 / 92.0 | 6.8 / 15.0 | 40.4 / 18.6 |
| NAs |  | 1 / 0 | 1 / 0 | 1 / 0 | 19 / 6 |

| (b) mothers at birth / weaning | Days postpartum | Weight (kg) | Length (cm) | Body condition ^3^ | Baseline cortisol (ng/ml) |
| --- | --- | --- | --- | --- | --- |
| Min. | 0 / 17 | 23.8 / 23.8 | 105.5 | 24.6 / 25.6 | 2.4 / 0.5 |
| 1st Qu. | 0 / 52 | 32.0 / 32.1 | 119.0 | 33.2 / 31.2 | 7.8 / 1.9 |
| Median | 0 / 62 | 35.4 / 34.6 | 124.2 | 35.7 / 33.9 | 9.8 / 2.8 |
| Mean | 0 / 59 | 34.9 / 34.7 | 123.6 | 35.7 / 34.1 | 11.1 / 3.2 |
| 3rd Qu. | 0 / 69 | 38.0 / 37.3 | 128.5 | 38.3 / 36.4 | 11.9 / 4.2 |
| Max. | 0 / 90 | 49.0 / 50.1 | 139.0 | 52.3 / 51.7 | 40.0 / 10.2 |
| NAs |  | 0 / 0 | 0 | 0 / 2 | 29 / 12 |

**Supplementary Table S2**. Summary statistics for immunity and oxidative stress markers. (a) For focal pups. (b) For focal mothers.

| (a) pups at  birth / molt | BKA: *S. aureus* (percent) | BKA: *E. coli* (percent) | hemagglutination | hemolysis | lysozyme  (µg/mL) | haptoglobin (mg/ml) | neopterin (nmol/l) | IgG  (µg/ml) |
| --- | --- | --- | --- | --- | --- | --- | --- | --- |
| Min. | -28.76 / -55.47 | -24.78 / -51.43 | 0 / 2 | 0 / 2 | 0.52 / 1.09 | 0.03 / 0.05 | 1.51 / 0.99 | 2050 / 6501 |
| 1st Qu. | -2.06 / -4.21 | 2.85 /7.06 | 2 / 5 | 0 / 4 | 4.34 / 2.73 | 0.61 / 0.68 | 2.66 / 1.35 | 8281 / 21478 |
| Median | 3.38 / 2.82 | 12.83 / 24.84 | 3 / 5 | 2 / 4 | 8.46 / 3.79 | 0.96 / 1.19 | 3.47 / 1.65 | 11430 / 28210 |
| Mean | 4.86 / 2.98 | 13.84 / 29.17 | 3 / 5 | 2 / 4 | 13.11 / 5.56 | 1.19 / 1.54 | 4.75 / 1.70 | 12258 / 28216 |
| 3rd Qu. | 12.41 / 13.36 | 26.44 / 45.81 | 3 / 6 | 3 / 5 | 14.74 / 6.07 | 1.48 / 1.99 | 5.67 / 2.03 | 15022 / 33228 |
| Max. | 30.09 / 37.28 | 98.43 / 97.34 | 5 / 8 | 4 / 7 | 92.22 / 53.03 | 3.89 / 6.61 | 20.13 / 2.88 | 28580 / 48360 |
| NAs | 5 / 0 | 5 / 0 | 31 / 2 | 31 / 2 | 0 / 0 | 15 / 0 | 35 / 4 | 0 / 1 |

| pups at  birth / molt | differential counts | | | | | OXY  (mM HOCl neutralized) | dROM  (mM H_2_O_2_ equivalents) | GPx | SOD |
| --- | --- | --- | --- | --- | --- | --- | --- | --- | --- |
|  | neutrophils | basophils | eosinophils | monocytes | lymphocytes |  |  |  |  |
| Min. | 0 / 7.0 | 0 / 0 | 0 / 0 | 0 / 0 | 11.0 / 15.0 | 234.4 / 225.40 | 0.30 / 0.89 | 460 / 302 | 0.2 / 0.1 |
| 1st Qu. | 28.0 / 28.5 | 0 / 0 | 4.0 / 2.0 | 3.0 / 2.0 | 30.0 / 34.5 | 285.5 / 281.60 | 0.87 / 1.89 | 1050 / 976 | 0.7 / 0.7 |
| Median | 44.5 / 40.0 | 0 / 0 | 7.0 / 5.0 | 5.0 / 4.0 | 39.0 / 49.0 | 311.4 / 305.10 | 1.16 / 2.39 | 1278 / 1159 | 0.9 / 0.9 |
| Mean | 41.8 / 39.9 | 0.6 / 0.9 | 7.1 / 5.3 | 5.9 / 4.9 | 44.3 / 48.7 | 309.8 / 303.90 | 1.20 / 2.64 | 1251 / 1252 | 1.0 / 1.0 |
| 3rd Qu. | 55.0 / 54.0 | 1.0 / 1.0 | 10.0 / 7.0 | 8.0 / 7.0 | 59.0 / 59.5 | 332.8 / 324.90 | 1.43 / 3.06 | 1411 / 1540 | 1.2 / 1.1 |
| Max. | 84.0 / 75.0 | 12.0 / 7.0 | 23.0 / 18.0 | 22.0 / 14.0 | 92.0 / 87.0 | 377.2 / 374.20 | 3.47 / 7.35 | 2159 / 2698 | 3.0 / 3.0 |
| NAs | 0 / 0 | 0 / 0 | 0 / 0 | 0 / 0 | 0 / 0 | 1 / 0 | 9 / 0 | 40 / 6 | 41 / 11 |

| (b) mothers after giving birth / molt | BKA: *S. aureus* (percent) | BKA: *E. coli* (percent) | hemagglutination | hemolysis | lysozyme (µg/mL) | haptoglobin (mg/ml) | neopterin (nmol/l) | IgG (µg/ml) |
| --- | --- | --- | --- | --- | --- | --- | --- | --- |
| Min. | -17.39 / -28.62 | -11.78 / -3.60 | 3 / 4 | 3 / 2 | 3.88 / 3.16 | 1.04 / 1.01 | 0.27 / 0.61 | 14670 / 15500 |
| 1st Qu. | 6.08 / -0.32 | 28.95 / 39.25 | 5 / 5 | 4 / 4 | 6.61 / 6.16 | 2.21 / 2.01 | 1.23 / 1.19 | 23830 / 24153 |
| Median | 29.56 / 6.35 | 66.04 / 55.97 | 5 / 5 | 4 / 5 | 9.10 / 7.51 | 3.19 / 2.73 | 1.44 / 1.36 | 29410 / 29060 |
| Mean | 45.77 / 9.64´4 | 58.39 / 57.13 | 5 / 5 | 4 / 4 | 10.77 / 8.19 | 3.33 / 3.00 | 1.57 / 1.39 | 32111 / 32492 |
| 3rd Qu. | 91.77 / 19.15 | 86.99 / 76.91 | 5 / 6 | 5 / 5 | 12.33 / 9.50 | 4.04 / 3.68 | 1.82 / 1.54 | 36410 / 35450 |
| Max. | 98.19 / 63.54 | 100 / 100 | 8 / 8 | 6 / 7 | 59.74 / 20.64 | 7.98 / 15.46 | 6.34 / 3.94 | 98200 / 172800 |
| NAs | 0 / 0 | 0 / 1 | 5 / 2 | 5 / 2 | 0 / 0 | 0 / 1 | 5 / 2 | 0 / 0 |

| mothers after giving birth / molt | differential counts | | | | | OXY  (mM HOCl neutralized) | dROM (mM H_2_O_2_ equivalents) | GPx | SOD |
| --- | --- | --- | --- | --- | --- | --- | --- | --- | --- |
|  | neutrophils | basophils | eosinophils | monocytes | lymphocytes |  |  |  |  |
| Min. | 16.0 / 18.0 | 0 / 0 | 1.0 / 1.0 | 0 / 0 | 5.0 / 20.0 | 249.20 / 253.20 | 0.96 / 1.03 | 603 / 373 | 0.2 / 0.3 |
| 1st Qu. | 44.5 / 32.0 | 0 / 0 | 6.0 / 5.0 | 3.0 /4.0 | 21.5 / 34.0 | 293.80 / 295.70 | 2.14 / 2.15 | 1258 / 1345 | 0.4 / 0.4 |
| Median | 55.0 / 40.0 | 0 / 0 | 9.0 / 9.0 | 5.0 / 6.0 | 27.0 / 41.5 | 310.30 / 314.30 | 3.02 / 2.88 | 1698 / 1810 | 0.4 / 0.5 |
| Mean | 53.2 / 39.6 | 0.7 / 0.7 | 9.2 / 10.3 | 5.5 / 6.2 | 31.2 / 42.9 | 321.50 / 324.20 | 3.07 / 2.83 | 1707 / 1778 | 0.6 / 0.5 |
| 3rd Qu. | 63.5 / 47.0 | 1.0 / 1.0 | 12.0 / 14.2 | 7.0 / 9.0 | 40.0 / 48.2 | 347.50 / 337.10 | 3.84 / 3.35 | 2119 / 2373 | 0.6 / 0.7 |
| Max. | 82.0 / 66.0 | 6.0 / 6.0 | 22.0 / 29.0 | 17.0 / 16.0 | 75.0 / 74.0 | 470.00 / 708.40 | 6.81 / 5.11 | 3262 / 3254 | 1.4 / 1.1 |
| NAs | 0 / 0 | 0 / 0 | 0 / 0 | 0 / 0 | 0 / 0 | 0 / 0 | 0 / 0 | 32 / 26 | 42 / 31 |

Supplementary Table S3. R-squared value for the linear models assessing the proportion of the variability in the response variable (immune or oxidative stress marker) that is explained by the time of day (hour) at which the sample was collected. Models were fitted using the R package lmer version 1.1-30 ^4^.

|  | **R-squared** |
| --- | --- |
| BKA (*S. aureus*) | 0.044 |
| BKA (*E. coli*) | 0.007 |
| hemagglutination | 0.004 |
| hemolysis | 0.012 |
| lysozyme | 0.000 |
| haptoglobin | 0.001 |
| neopterin | 0.000 |
| IgG | 0.002 |
| Innate / adaptive WBC ratio | 0.007 |
| OXY | 0.000 |
| dROM | 0.008 |
| GPx | 0.002 |
| SOD | 0.008 |

Supplementary Table S4. Repeatability estimate and 95% highest posterior density intervals (HPDI) of immune marker concentrations in pups and mothers assessed at birth and approximately 60 days later, as individuals began to molt and pups weaned. Repeatability was calculated according to ^5^ as R = V_ID_ / (V_ID_ + V_F_ + V_R_), where V_ID_ stands for between-individual variance (here, also random-effect variance), V_F_ for the fixed-effect variance, and V_R_ for the residual variance. BKA, bacterial killing assay; Ig, immunoglobulin; WBC, white blood cell

|  | pups | 95% HPDI | | mothers | 95% HPDI | |
| --- | --- | --- | --- | --- | --- | --- |
|  | *mean* | *lower* | *upper* | *mean* | *lower* | *upper* |
| BKA (*S. aureus*) | 0.26 | 0.12 | 0.41 | 0.13 | 0.07 | 0.20 |
| BKA (*E. coli*) | 0.25 | 0.12 | 0.39 | 0.18 | 0.08 | 0.29 |
| hemagglutination | 0.17 | 0.09 | 0.26 | 0.18 | 0.09 | 0.28 |
| hemolysis | 0.14 | 0.07 | 0.21 | 0.17 | 0.08 | 0.28 |
| lysozyme | 0.24 | 0.13 | 0.37 | 0.19 | 0.08 | 0.30 |
| haptoglobin | 0.27 | 0.12 | 0.42 | 0.31 | 0.12 | 0.48 |
| neopterin | 0.17 | 0.09 | 0.27 | 0.49 | 0.26 | 0.72 |
| IgG | 0.19 | 0.10 | 0.28 | 0.19 | 0.09 | 0.30 |
| WBC count (innate/adaptive) | 0.24 | 0.12 | 0.37 | 0.23 | 0.11 | 0.37 |

Supplementary Table S5. Posterior means and 95% highest posterior density intervals (HPDI) for repeatability estimates from a null model including only individual ID as a random effect [R = V_ID_ / (V_ID_ + V_e_)]. This provides a “simple estimate of the phenotypic variance observed in the raw data” ^6^. BKA, bacterial killing assay; Ig, immunoglobulin; WBC, white blood cell

|  | pups | 95% HPDI | | mothers | 95% HPDI | |
| --- | --- | --- | --- | --- | --- | --- |
|  | *mean* | *lower* | *upper* | *mean* | *lower* | *upper* |
| BKA (*S. aureus*) | 0.28 | 0.13 | 0.43 | 0.16 | 0.08 | 0.25 |
| BKA (*E. coli*) | 0.22 | 0.12 | 0.35 | 0.26 | 0.14 | 0.40 |
| hemagglutination | 0.18 | 0.09 | 0.28 | 0.19 | 0.10 | 0.30 |
| hemolysis | 0.17 | 0.08 | 0.25 | 0.25 | 0.12 | 0.37 |
| lysozyme | 0.19 | 0.09 | 0.29 | 0.18 | 0.10 | 0.29 |
| haptoglobin | 0.27 | 0.13 | 0.41 | 0.23 | 0.12 | 0.35 |
| neopterin | 0.20 | 0.10 | 0.32 | 0.20 | 0.10 | 0.31 |
| IgG | 0.14 | 0.07 | 0.21 | 0.24 | 0.12 | 0.36 |
| WBC count (innate/adaptive) | 0.26 | 0.13 | 0.39 | 0.22 | 0.12 | 0.34 |

Supplementary Table S6. Pairwise comparisons of group means. The estimate, standard error, t-score, adjusted p-value, and Cohen’s d measure of effect size are provided. Negative t-scores and Cohen’s d values indicate a lower concentration at birth compared to weaning. Large (> |0.8|) and moderate (> |0.5|) effects sizes are marked in red. Box plots of raw data can be seen in supplementary figures S1 and S2.

| marker | comparisons: group 1 | group 2 | estimate | std error | t |  | adj p | Cohen's d | |
| --- | --- | --- | --- | --- | --- | --- | --- | --- | --- |
| BKA  *S. aureus* | mother, birth | mother, weaning | -36.14 | 3.81 | -9.49 |  | 0.00 | 1.14 | large |
|  | pup, birth | pup, weaning | -1.87 | 4.03 | -0.46 |  | 0.97 | 0.13 | negligible |
|  | mother, birth | pup, birth | -40.92 | 3.75 | -10.91 |  | 0.00 | 1.34 | large |
|  | mother, weaning | pup, weaning | -6.65 | 4.08 | -1.63 |  | 0.36 | 0.40 | small |
| BKA  *E. coli* | mother, birth | mother, weaning | -1.26 | 4.12 | -0.30 |  | 0.99 | 0.04 | negligible |
|  | pup, birth | pup, weaning | 15.33 | 4.34 | 3.53 |  | 0.00 | -0.62 | moderate |
|  | mother, birth | pup, birth | -44.55 | 4.05 | -11.00 |  | 0.00 | 1.70 | large |
|  | mother, weaning | pup, weaning | -27.96 | 4.41 | -6.34 |  | 0.00 | 0.98 | large |
| hem-  agglutination | mother, birth | mother, weaning | 0.38 | 0.16 | 2.39 |  | 0.08 | -0.43 | small |
|  | pup, birth | pup, weaning | 2.55 | 0.18 | 14.30 |  | 0.00 | -2.11 | large |
|  | mother, birth | pup, birth | -2.59 | 0.17 | -15.33 |  | 0.00 | 2.38 | large |
|  | mother, weaning | pup, weaning | -0.41 | 0.17 | -2.47 |  | 0.07 | 0.41 | small |
| hemolysis | mother, birth | mother, weaning | 0.40 | 0.15 | 2.59 |  | 0.05 | -0.48 | small |
|  | pup, birth | pup, weaning | 2.58 | 0.17 | 14.89 |  | 0.00 | -2.18 | large |
|  | mother, birth | pup, birth | -2.54 | 0.16 | -15.50 |  | 0.00 | 2.28 | large |
|  | mother, weaning | pup, weaning | -0.36 | 0.16 | -2.21 |  | 0.12 | 0.39 | small |
| lysozyme | mother, birth | mother, weaning | -2.58 | 1.43 | -1.81 |  | 0.27 | 0.47 | small |
|  | pup, birth | pup, weaning | -7.55 | 1.49 | -5.07 |  | 0.00 | 0.64 | moderate |
|  | mother, birth | pup, birth | 2.34 | 1.38 | 1.69 |  | 0.33 | -0.20 | negligible |
|  | mother, weaning | pup, weaning | -2.63 | 1.53 | -1.72 |  | 0.31 | 0.47 | small |
| haptoglobin | mother, birth | mother, weaning | -0.33 | 0.22 | -1.50 |  | 0.44 | 0.19 | negligible |
|  | pup, birth | pup, weaning | 0.35 | 0.24 | 1.50 |  | 0.44 | -0.32 | small |
|  | mother, birth | pup, birth | -2.14 | 0.22 | -9.73 |  | 0.00 | 1.72 | large |
|  | mother, weaning | pup, weaning | -1.46 | 0.23 | -6.29 |  | 0.00 | 0.92 | large |
| neopterin | mother, birth | mother, weaning | -0.18 | 0.26 | -0.69 |  | 0.90 | 0.29 | small |
|  | pup, birth | pup, weaning | -3.04 | 0.30 | -10.18 |  | 0.00 | 1.19 | large |
|  | mother, birth | pup, birth | 3.18 | 0.28 | 11.29 |  | 0.00 | -1.23 | large |
|  | mother, weaning | pup, weaning | 0.31 | 0.28 | 1.14 |  | 0.66 | -0.72 | moderate |
| IgG | mother, birth | mother, weaning | 380.93 | 1853.42 | 0.21 |  | 1.00 | -0.02 | negligible |
|  | pup, birth | pup, weaning | 15958.01 | 1943.91 | 8.21 |  | 0.00 | -2.06 | large |
|  | mother, birth | pup, birth | -19852.74 | 1800.87 | -11.02 |  | 0.00 | 2.06 | large |
|  | mother, weaning | pup, weaning | -4275.66 | 1992.69 | -2.15 |  | 0.14 | 0.29 | small |
| WBC count (innate/adaptive) | mother, birth | mother, weaning | -1.93 | 0.30 | -6.28 |  | 0.00 | 0.77 | moderate |
|  | pup, birth | pup, weaning | -0.44 | 0.31 | -1.37 |  | 0.51 | 0.33 | small |
|  | mother, birth | pup, birth | -1.62 | 0.29 | -5.54 |  | 0.00 | 0.61 | moderate |
|  | mother, weaning | pup, weaning | -0.13 | 0.32 | -0.39 |  | 0.97 | 0.14 | negligible |
| OXY | mother, birth | mother, weaning | 2.66 | 6.41 | 0.42 |  | 0.98 | -0.05 | negligible |
|  | pup, birth | pup, weaning | -5.89 | 6.71 | -0.88 |  | 0.82 | 0.18 | negligible |
|  | mother, birth | pup, birth | -11.74 | 6.24 | -1.88 |  | 0.24 | 0.32 | small |
|  | mother, weaning | pup, weaning | -20.29 | 6.86 | -2.96 |  | 0.02 | 0.43 | small |
| dROM | mother, birth | mother, weaning | -0.23 | 0.15 | -1.51 |  | 0.43 | 0.21 | small |
|  | pup, birth | pup, weaning | 1.44 | 0.16 | 8.93 |  | 0.00 | -1.59 | large |
|  | mother, birth | pup, birth | -1.86 | 0.15 | -12.35 |  | 0.00 | 1.97 | large |
|  | mother, weaning | pup, weaning | -0.19 | 0.16 | -1.18 |  | 0.64 | 0.18 | negligible |
| GPx | mother, birth | mother, weaning | 216.32 | 97.60 | 2.22 |  | 0.12 | -0.34 | small |
|  | pup, birth | pup, weaning | 0.86 | 98.37 | 0.01 |  | 0.99 | -0.00 | negligible |
|  | mother, birth | pup, birth | -380.61 | 99.44 | -3.83 |  | 0.00 | 0.75 | moderate |
|  | mother, weaning | pup, weaning | -596.08 | 96.50 | -6.18 |  | 0.00 | 1.07 | large |
| SOD | mother, birth | mother, weaning | -0.14 | 0.08 | -1.65 |  | 0.35 | 0.53 | moderate |
|  | pup, birth | pup, weaning | -0.28 | 0.08 | -0.34 |  | 0.98 | 0.05 | negligible |
|  | mother, birth | pup, birth | 0.41 | 0.08 | 4.06 |  | 0.00 | -0.93 | large |
|  | mother, weaning | pup, weaning | 0.52 | 0.08 | 6.33 |  | 0.00 | -1.27 | large |

References:

1. Kassambara, A. ggpubr: ‘ggplot2’ Based Publication Ready Plots. (2020).

2. Bland, A. Calculating correlation coefficients with repeated observations: Part 2 - correlation between subjects. *BMJ* **310**, 633 (1995).

3. Peig, J. & Green, A. J. New perspectives for estimating body condition from mass/length data: The scaled mass index as an alternative method. *Oikos* **118**, 1883–1891 (2009).

4. Bates, D., Mächler, M., Bolker, B. & Walker, S. Fitting linear mixed-effects models using lme4. *Journal of Statistical Software* **67**, 1–48 (2015).

5. de Villemereuil, P., Morrissey, M. B., Nakagawa, S. & Schielzeth, H. Fixed-effect variance and the estimation of repeatabilities and heritabilities: issues and solutions. *Journal of Evolutionary Biology* **31**, 621–632 (2018).

6. Wilson, A. J. Why h2 does not always equal VA/VP? *Journal of Evolutionary Biology* **21**, 647–650 (2008).
