# Supplementary material for: Life-history stage influences immune investment and oxidative stress in response to environmental heterogeneity in Antarctic fur seals
Source: Commun Biol. 2024 Jun 29;7:788. doi: 10.1038/s42003-024-06499-6 (PMC11217341; doi:10.1038/s42003-024-06499-6)
Supplement: Supplementary file 2 — Description of Additional Supplementary Files [file 42003_2024_6499_MOESM2_ESM.docx]

Description of Additional Supplementary Files

**File name:** Supplementary Data 1

**Description:** Summary of models for pups. Generalized linear mixed models were fitted in a Bayesian framework using Markov chain Monte Carlo methods in the R package MCMCglmm version 2.34 ^1,2^. The point estimate of the posterior mean and 95% highest posterior density intervals (HPDI), effective sample size, and *p*MCMC value for each predictor variable are provided.

**File name:** Supplementary Data 2

**Description:** Summary of models for mothers. Generalized linear mixed models were fitted in a Bayesian framework using Markov chain Monte Carlo methods in the R package MCMCglmm version 2.34 ^1,2^. The point estimate of the posterior mean and 95% highest posterior density intervals (HPDI), effective sample size, and *p*MCMC value for each predictor variable are provided.

1. Hadfield, J. D. MCMC methods for multi-response generalized linear mixed models: The MCMCglmm R package. *Journal of Statistical Software* **33**, 1–22 (2010).

2. Hadfield, J. Markov chain Monte Carlo generalised linear mixed models - Course Notes. (2019).
