# Supplementary material for: Life-history stage influences immune investment and oxidative stress in response to environmental heterogeneity in Antarctic fur seals
Source: Commun Biol. 2024 Jun 29;7:788. doi: 10.1038/s42003-024-06499-6 (PMC11217341; doi:10.1038/s42003-024-06499-6)
Supplement: Supplementary file 3 — Supplementary Data 1 [file 42003_2024_6499_MOESM3_ESM.docx]

Supplementary Data Table 1. Summary of models for pups. Generalized linear mixed models were fitted in a Bayesian framework using Markov chain Monte Carlo methods in the R package MCMCglmm version 2.34 ^1,2^. The point estimate of the posterior mean and 95% highest posterior density intervals (HPDI), effective sample size, and *p*MCMC value for each predictor variable are provided.

| **BKA (*S. saureus*) ~** | **Estimate** | **Effect** | **Mean [95% HPDI]** | **Effective *n*** | ***p*MCMC** |
| --- | --- | --- | --- | --- | --- |
| cortisol + beach + sex | intercept |  | 0.03 [-0.36 - 0.53] | 1000 | 0.86 |
|  | cortisol | direct, total | 0.01 [-0.01 - 0.03] | 1000 | 0.62 |
|  | beach: FWB | direct | -0.31 [-0.75 - 0.06] | 1075 | 0.13 |
|  | sex: F | direct | 0.13 [-0.30 - 0.49] | 1000 | 0.49 |
| season + cortisol + beach + sex | intercept |  | 0.25 [-0.19 - 0.72] | 1000 | 0.27 |
|  | season: 2019 | direct | -0.49 [-0.88 - -0.05] | 1000 | 0.02 |
| season | intercept |  | 0.25 [-0.02 – 0.48] | 1000 | 0.06 |
|  | season: 2019 | total | -0.47 [-0.80 - -0.11] | 1000 | 0.01 |
| beach + season | intercept |  | 0.40 [0.09 - 0.69] | 1321 | 0.008 |
|  | beach: FWB | total | -0.31 [-0.67 - 0.02] | 1312 | 0.07 |
| sex | intercept |  | -0.07 [-0.31 - 0.16] | 1000 | 0.53 |
|  | sex: F | total | 0.16 [-0.17 - 0.52] | 1000 | 0.38 |
| condition | intercept |  | 0.33 [-0.09 - 0.78] | 1094 | 0.14 |
|  | condition | n/a | -0.04 [-0.09 - 0.01] | 1000 | 0.10 |

| **BKA (*E. coli*) ~** | **Estimate** | **Effect** | **Mean [95% HPDI]** | **Effective *n*** | ***p*MCMC** |
| --- | --- | --- | --- | --- | --- |
| cortisol + beach + sex | intercept |  | 0.50 [0.08 - 0.91] | 1106 | 0.02 |
|  | cortisol | direct, total | -0.04 [-0.06 - -0.02] | 1093 | 0.002 |
|  | beach: FWB | direct | -0.32 [-0.73 - 0.02] | 806 | 0.10 |
|  | sex: F | direct | 0.05 [-0.30 - 0.44] | 1000 | 0.78 |
| season + cortisol + beach + sex | intercept |  | 0.50 [0.03 - 1.01] | 1000 | 0.04 |
|  | season: 2019 | direct | -0.01 [-0.35 - 0.39] | 1000 | 0.97 |
| season | intercept |  | 0.01 [-0.22 – 0.28] | 1000 | 0.91 |
|  | season: 2019 | total | -0.05 [-0.36 – 0.33] | 1000 | 0.78 |
| beach + season | intercept |  | 0.18 [-0.10 - 0.49] | 1000 | 0.21 |
|  | beach: FWB | total | -0.34 [-0.70 - -0.01] | 1000 | 0.05 |
| sex | intercept |  | -0.09 [-0.35 - 0.14] | 1000 | 0.45 |
|  | sex: F | total | 0.18 [-0.18 - 0.54] | 1000 | 0.32 |
| condition | intercept |  | -0.81 [-1.21 - -0.38] | 1111 | 0.002 |
|  | condition | n/a | 0.10 [0.05 - 0.14] | 1119 | 0.002 |

| **hemagglutination ~** | **Estimate** | **Effect** | **Mean [95% HPDI]** | **Effective *n*** | ***p*MCMC** |
| --- | --- | --- | --- | --- | --- |
| cortisol + beach + sex | intercept |  | 0.89 [0.52 - 1.35 ] | 1170 | <0.001 |
|  | cortisol | direct, total | -0.08 [-0.10 - -0.06] | 1000 | <0.001 |
|  | beach: FWB | direct | -0.20 [-0.59 - 0.18] | 1000 | 0.29 |
|  | sex: F | direct | -0.03 [-0.40 - 0.34] | 1000 | 0.85 |
| season + cortisol + beach + sex | intercept |  | 0.79 [0.40 – 1.24] | 1000 | <0.001 |
|  | season: 2019 | direct | 0.29 [-0.09 - 0.65] | 1000 | 0.14 |
| season | intercept |  | -0.01 [-0.31 - 0.33] | 1000 | 0.99 |
|  | season: 2019 | total | 0.07 [-0.33 – 0.53] | 1000 | 0.74 |
| beach + season | intercept |  | 0.04 [-0.28 - 0.38] | 1000 | 0.79 |
|  | beach: FWB | total | -0.09 [-0.53 - 0.27] | 782 | 0.64 |
| sex | intercept |  | -0.01 [-0.31 - 0.25] | 1000 | 0.92 |
|  | sex: F | total | 0.03 [-0.37 - 0.42] | 1000 | 0.84 |
| condition | intercept |  | -2.09 [-2.51 - -1.74] | 1000 | <0.001 |
|  | condition | n/a | 0.25 [0.20 - 0.29] | 1096 | <0.001 |

| **hemolysis ~** | **Estimate** | **Effect** | **Mean [95% HPDI]** | **Effective *n*** | ***p*MCMC** |
| --- | --- | --- | --- | --- | --- |
| cortisol + beach + sex | intercept |  | 0.99 [0.59 - 1.38] | 1000 | <0.001 |
|  | cortisol | direct, total | -0.08 [-0.10 - -0.06] | 1000 | <0.001 |
|  | beach: FWB | direct | -0.33 [-0.70 - 0.05] | 1000 | 0.09 |
|  | sex: F | direct | -0.09 [-0.44 - 0.27] | 2081 | 0.61 |
| season + cortisol + beach + sex | intercept |  | 0.86 [0.41 - 1.29] | 1000 | <0.001 |
|  | season:2019 | direct | 0.29 [-0.10 - 0.65] | 822 | 0.15 |
| season | intercept |  | -0.02 [-0.31 – 0.30] | 1000 | 0.88 |
|  | season:2019 | total | 0.03 [-0.35 – 0.46] | 1000 | 0.85 |
| beach + season | intercept |  | 0.04 [-0.30 - 0.38] | 1000 | 0.77 |
|  | beach: FWB | total | -0.19 [-0.60 - 0.17] | 1000 | 0.33 |
| sex | intercept |  | -0.03 [-0.29 - 0.23] | 1000 | 0.75 |
|  | sex: F | total | 0.05 [-0.30 - 0.43] | 1000 | 0.78 |
| condition | intercept |  | -2.09 [-2.52 - -1.71] | 1000 | <0.001 |
|  | condition | n/a | 0.25 [0.20 - 0.30] | 1000 | <0.001 |

| **lysozyme ~** | **Estimate** | **Effect** | **Mean [95% HPDI]** | **Effective *n*** | ***p*MCMC** |
| --- | --- | --- | --- | --- | --- |
| cortisol + beach + sex | intercept |  | -0.53 [-0.93 - -0.07] | 1000 | 0.01 |
|  | cortisol | direct, total | 0.04 [0.01 - 0.06] | 1095 | <0.001 |
|  | beach: FWB | direct | 0.04 [-0.30 - 0.45] | 1110 | 0.85 |
|  | sex: F | direct | 0.18 [-0.17 - 0.54] | 1000 | 0.32 |
| season + cortisol + beach + sex | intercept |  | -0.50 [-0.91 - -0.04] | 1000 | 0.02 |
|  | season:2019 | direct | -0.03 [-0.37 - 0.31] | 1000 | 0.86 |
| season | intercept |  | -0.02 [-0.25 – 0.23] | 1000 | 0.86 |
|  | season:2019 | total | 0.03 [-0.28 – 0.39] | 1000 | 0.86 |
| beach + season | intercept |  | -0.03 [-0.34 - 0.24] | 1000 | 0.81 |
|  | beach: FWB | total | 0.04 [-0.29 - 0.41] | 1000 | 0.83 |
| sex | intercept |  | -0.12 [-0.34 - 0.12] | 1000 | 0.33 |
|  | sex: F | total | 0.24 [-0.07 - 0.58] | 886 | 0.16 |
| condition | intercept |  | 0.84 [0.44 - 1.29] | 1272 | <0.001 |
|  | condition | n/a | -0.10 [-0.15 - -0.05] | 1181 | <0.001 |

| **haptoglobin ~** | **Estimate** | **Effect** | **Mean [95% HPDI]** | **Effective *n*** | ***p*MCMC** |
| --- | --- | --- | --- | --- | --- |
| cortisol + beach + sex | intercept |  | 0.20 [-0.25 - 0.62] | 1123 | 0.36 |
|  | cortisol | direct, total | -0.01 [-0.03 - 0.01] | 1122 | 0.56 |
|  | beach: FWB | direct | -0.11 [-0.49 - 0.28] | 886 | 0.62 |
|  | sex: F | direct | -0.06 [-0.46 - 0.34] | 1000 | 0.78 |
| season + cortisol + beach + sex | intercept |  | 0.44 [0.01 - 0.92] | 882 | 0.05 |
|  | season: 2019 | direct | -0.54 [-0.94 - -0.14] | 1000 | 0.14 |
| season | intercept |  | 0.28 [0.04 – 0.54] | 1102 | 0.02 |
|  | season: 2019 | total | -0.56 [-0.92 - -0.20] | 1000 | <0.001 |
| beach + season | intercept |  | 0.36 [0.06 - 0.66] | 1000 | 0.02 |
|  | beach: FWB | total | -0.15 [-0.50 - 0.17] | 783 | 0.37 |
| sex | intercept |  | -0.04 [-0.30 - 0.19] | 1262 | 0.77 |
|  | sex: F | total | 0.07 [-0.26 - 0.45] | 1216 | 0.69 |
| condition | intercept |  | -0.51 [-0.92 - -0.04] | 1273 | 0.03 |
|  | condition | n/a | 0.06 [0.01 - 0.11] | 1000 | 0.01 |

| **neopterin ~** | **Estimate** | **Effect** | **Mean [95% HPDI]** | **Effective *n*** | ***p*MCMC** |
| --- | --- | --- | --- | --- | --- |
| cortisol + beach + sex | intercept |  | -0.89 [-1.32 - -0.50] | 1000 | <0.001 |
|  | cortisol | direct, total | 0.08 [0.05 - 0.10] | 1317 | <0.001 |
|  | beach: FWB | direct | 0.33 [-0.04 - 0.74] | 1000 | 0.09 |
|  | sex: F | direct | 0.01 [-0.35 - 0.39] | 732 | 0.96 |
| season + cortisol + beach + sex | intercept |  | -0.97 [-1.40 - -0.53] | 1000 | <0.001 |
|  | season: 2019 | direct | 0.19 [-0.22 - 0.57] | 1000 | 0.32 |
| season | intercept |  | -0.24 [-0.49 – 0.06] | 1000 | 0.10 |
|  | season: 2019 | total | 0.43 [0.05 – 0.83] | 1000 | 0.03 |
| beach + season | intercept |  | -0.31 [-0.63 - 0.07] | 1149 | 0.08 |
|  | beach: FWB | total | 0.16 [-0.21 - 0.56] | 1000 | 0.44 |
| sex | intercept |  | -0.02 [-0.33 - 0.22] | 906 | 0.83 |
|  | sex: F | total | 0.04 [-0.34 - 0.40] | 1000 | 0.85 |
| condition | intercept |  | 1.44 [0.95 - 1.92] | 1000 | <0.001 |
|  | condition | n/a | -0.17 [-0.22 - -0.11] | 1000 | <0.001 |

| **IgG ~** | **Estimate** | **Effect** | **Mean [95% HPDI]** | **Effective *n*** | ***p*MCMC** |
| --- | --- | --- | --- | --- | --- |
| cortisol + beach + sex | intercept |  | 0.80 [0.41 - 1.22] | 1000 | <0.001 |
|  | cortisol | direct, total | -0.06 [-0.08 - -0.03] | 1000 | <0.001 |
|  | beach: FWB | direct | -0.14 [-0.50 - 0.18] | 1000 | 0.42 |
|  | sex: F | direct | -0.22 [-0.56 - 0.13] | 926 | 0.22 |
| season + cortisol + beach + sex | intercept |  | 0.71 [0.27 - 1.16] | 1742 | 0.004 |
|  | season: 2019 | direct | 0.18 [-0.13 - 0.61] | 1113 | 0.30 |
| season | intercept |  | -0.02 [-0.29 – 0.21] | 1122 | 0.81 |
|  | season: 2019 | total | 0.03 [-0.33 – 0.35] | 1000 | 0.83 |
| beach + season | intercept |  | 0.03 [-0.25 - 0.34] | 1000 | 0.80 |
|  | beach: FWB | total | -0.13 [-0.48 - 0.22] | 1000 | 0.46 |
| sex | intercept |  | 0.06 [-0.15 - 0.31] | 1000 | 0.56 |
|  | sex: F | total | -0.16 [-0.48 - 0.23] | 884 | 0.37 |
| condition | intercept |  | -1.87 [-2.21 - -1.51] | 1000 | <0.001 |
|  | condition | n/a | 0.24 [0.19 - 0.28] | 1000 | <0.001 |

| **WBC (innate / adaptive) ~** | **Estimate** | **Effect** | **Mean [95% HPDI]** | **Effective *n*** | ***p*MCMC** |
| --- | --- | --- | --- | --- | --- |
| cortisol + beach + sex | intercept |  | -0.07 [-0.53 – 0.30] | 1000 | 0.68 |
|  | cortisol | direct, total | 0.01 [-0.01 – 0.03] | 1000 | 0.29 |
|  | beach: FWB | direct | -0.11 [-0.48 – 0.24] | 897 | 0.55 |
|  | sex: F | direct | -0.04 [-0.44 – 0.29] | 1340 | 0.84 |
| season + cortisol + beach + sex | intercept |  | 0.10 [-0.38 – 0.53] | 1000 | 0.62 |
|  | season: 2019 | direct | -0.43 [-0.80 - -0.07] | 1000 | 0.01 |
| season | intercept |  | 0.19 [-0.04 – 0.43] | 1000 | 0.11 |
|  | season: 2019 | total | -0.37 [-0.73 - -0.05] | 1000 | 0.03 |
| beach + season | intercept |  | 0.25 [-0.02 – 0.55] | 844 | 0.08 |
|  | beach: FWB | total | -0.12 [-0.45 – 0.17] | 1000 | 0.44 |
| sex | intercept |  | 0.05 [-0.17 – 0.30] | 1000 | 0.64 |
|  | sex: F | total | -0.12 [-0.47 – 0.23] | 1231 | 0.47 |
| condition | intercept |  | 0.34 [-0.09 – 0.72] | 902 | 0.11 |
|  | condition | n/a | -0.04 [-0.09 – 0.00] | 859 | 0.09 |

| **dROM ~** | **Estimate** | **Mean [95% HPDI]** | **Effective *n*** | ***p*MCMC** |
| --- | --- | --- | --- | --- |
| hemagglutination | intercept | 0.10 [-0.06 - 0.29] | 1000 | 0.26 |
|  | hemagglutination | 0.52 [0.38 – 0.68] | 1000 | <0.001 |
| hemolysis | intercept | 0.10 [-0.09 - 0.28] | 1000 | 0.29 |
|  | hemolysis | 0.52 [0.38 – 0.65] | 1000 | <0.001 |
| lysozyme | intercept | -0.01 [-0.18 – 0.17] | 1000 | 0.85 |
|  | lysozyme | -0.27 [-0.43 - -0.10] | 982 | <0.001 |
| haptoglobin | intercept | 0.04 [-0.11 - 0.20] | 1000 | 0.59 |
|  | haptoglobin | 0.50 [0.35 - 0.65] | 1000 | <0.001 |
| BKA (*E. coli*) | intercept | -0.01 [-0.17 - 0.17] | 910 | 0.95 |
|  | BKA (*E. coli*) | 0.07 [-0.08 - 0.23] | 1000 | 0.34 |
| BKA (*S. aureus*) | intercept | -0.01 [-0.18 - 0.15] | 1000 | 0.91 |
|  | BKA (*S. aureus*) | 0.14 [-0.00 - 0.32] | 1000 | 0.07 |
| neopterin | intercept | 0.13 [-0.05 - 0.32] | 1000 | 0.17 |
|  | neopterin | -0.39 [-0.55 - -0.22] | 1022 | <0.001 |
| IgG | intercept | -0.03 [-0.20 - 0.11] | 867 | 0.64 |
|  | IgG | 0.53 [0.39 - 0.66] | 1000 | <0.001 |
| WBC count (innate / adaptive) | intercept | 0.00 [-0.18 – 0.17] | 1034 | 0.99 |
|  | ratio | -0.07 [-0.24 – 0.07] | 1000 | 0.33 |

| **OXY ~** | **Estimate** | **Mean [95% HPDI]** | **Effective *n*** | ***p*MCMC** |
| --- | --- | --- | --- | --- |
| BKA (*S. aureus*) | intercept | -0.01 [-0.20 - 0.14] | 1000 | 0.85 |
|  | BKA (*S. aureus*) | 0.03 [-0.13 - 0.18] | 1196 | 0.66 |
| BKA (*E. coli*) | intercept | -0.01 [-0.18 - 0.16] | 1143 | 0.90 |
|  | BKA (*E. coli*) | -0.05 [-0.20 - 0.09] | 1003 | 0.50 |
| hemagglutination | intercept | -0.04 [-0.24 - 0.12] | 1236 | 0.64 |
|  | hemagglutination | -0.09 [-0.28 - 0.06] | 1000 | 0.27 |
| hemolysis | intercept | -0.04 [-0.23 - 0.13] | 842 | 0.64 |
|  | hemolysis | -0.11 [-0.29 - 0.05] | 1000 | 0.22 |
| lysozyme | intercept | 0.01 [-0.16 - 0.18] | 1000 | 0.94 |
|  | lysozyme | 0.07 [-0.09 - 0.22] | 1350 | 0.35 |
| haptoglobin | intercept | -0.01 [-0.18 - 0.14] | 1000 | 0.86 |
|  | haptoglobin | 0.01 [-0.15 - 0.15] | 1000 | 0.91 |
| neopterin | intercept | -0.09 [-0.26 - 0.09] | 1000 | 0.34 |
|  | neopterin | 0.20 [0.03 - 0.40] | 1000 | 0.04 |
| IgG | intercept | 0.00 [-0.15 - 0.19] | 1590 | 0.98 |
|  | IgG | -0.10 [-0.25 - 0.04] | 1000 | 0.17 |
| WBC count (innate / adaptive) | intercept | -0.00 [-0.18 – 0.16] | 1094 | 0.96 |
|  | ratio | -0.00 [-0.17 – 0.13] | 1000 | 0.97 |

| **GPx ~** | **Estimate** | **Mean [95% HPDI]** | **Effective *n*** | ***p*MCMC** |
| --- | --- | --- | --- | --- |
| BKA (*S. aureus*) | intercept | -0.03 [-0.24 - 0.17] | 910 | 0.74 |
|  | BKA (*S. aureus*) | 0.02 [-0.16 - 0.19] | 1000 | 0.91 |
| BKA (*E. coli*) | intercept | -0.02 [-0.23 - 0.17] | 1000 | 0.83 |
|  | BKA (*E. coli*) | -0.01 [-0.20 - 0.17] | 1000 | 0.94 |
| hemagglutination | intercept | -0.03 [-0.27 - 0.19] | 1000 | 0.76 |
|  | hemagglutination | -0.11 [-0.32 - 0.11] | 1118 | 0.29 |
| hemolysis | intercept | -0.01 [-0.24 - 0.21] | 902 | 0.89 |
|  | hemolysis | -0.15 [-0.36 - 0.07] | 1000 | 0.18 |
| lysozyme | intercept | -0.02 [-0.21 - 0.18] | 894 | 0.83 |
|  | lysozyme | 0.11 [-0.08 - 0.29] | 1000 | 0.24 |
| haptoglobin | intercept | -0.02 [-0.25 - 0.18] | 1000 | 0.87 |
|  | haptoglobin | 0.05 [-0.11 - 0.24] | 1000 | 0.54 |
| neopterin | intercept | -0.07 [-0.30 - 0.16] | 1000 | 0.56 |
|  | neopterin | -0.04 [-0.33 - 0.24] | 1000 | 0.78 |
| IgG | intercept | -0.04 [-0.23 - 0.16] | 1000 | 0.73 |
|  | IgG | 0.06 [-0.11 - 0.20] | 1000 | 0.48 |
| WBC count (innate / adaptive) | intercept | -0.03 [-0.24 – 0.17] | 810 | 0.75 |
|  | ratio | -0.02 [-0.19 – 0.16] | 1000 | 0.82 |

| **SOD ~** | **Estimate** | **Mean [95% HPDI]** | **Effective *n*** | ***p*MCMC** |
| --- | --- | --- | --- | --- |
| BKA (*S. aureus*) | intercept | 0.02 [-0.16 - 0.25] | 883 | 0.80 |
|  | BKA (*S. aureus*) | -0.16 [-0.36 - 0.083] | 1000 | 0.10 |
| BKA (*E. coli*) | intercept | 0.03 [-0.18 - 0.24] | 1000 | 0.73 |
|  | BKA (*E. coli*) | -0.13 [-0.30 - 0.05] | 1000 | 0.14 |
| hemagglutination | intercept | -0.06 [-0.26 - 0.15] | 1000 | 0.57 |
|  | hemagglutination | -0.00 [-0.16 - 0.15] | 1000 | 0.96 |
| hemolysis | intercept | -0.08 [-0.28 - 0.14] | 911 | 0.44 |
|  | hemolysis | 0.01 [-0.15 - 0.15] | 1000 | 0.85 |
| lysozyme | intercept | 0.02 [-0.19 - 0.22] | 1000 | 0.83 |
|  | lysozyme | -0.00 [-0.22 - 0.17] | 1000 | 0.98 |
| haptoglobin | intercept | 0.01 [-0.22 - 0.21] | 1000 | 0.95 |
|  | haptoglobin | -0.06 [-0.24 - 0.10] | 1000 | 0.52 |
| neopterin | intercept | -0.06 [-0.26 - 0.15] | 1077 | 0.60 |
|  | neopterin | 0.00 [-0.16 - 0.20] | 1000 | 0.99 |
| IgG | intercept | -0.04 [-0.16 - 0.26] | 1000 | 0.69 |
|  | IgG | -0.11 [-0.27 - 0.05] | 1000 | 0.19 |
| WBC count (innate / adaptive) | intercept | 0.04 [-0.17 – 0.25] | 1000 | 0.74 |
|  | ratio | -0.00 [-0.17 – 0.19] | 1000 | 0.96 |

1. Hadfield, J. D. MCMC methods for multi-response generalized linear mixed models: The MCMCglmm R package. *Journal of Statistical Software* **33**, 1–22 (2010).

2. Hadfield, J. Markov chain Monte Carlo generalised linear mixed models - Course Notes. (2019).
