# Supplementary material for: Life-history stage influences immune investment and oxidative stress in response to environmental heterogeneity in Antarctic fur seals
Source: Commun Biol. 2024 Jun 29;7:788. doi: 10.1038/s42003-024-06499-6 (PMC11217341; doi:10.1038/s42003-024-06499-6)
Supplement: Supplementary file 4 — Supplementary Data 2 [file 42003_2024_6499_MOESM4_ESM.docx]

Supplementary Data Table 2. Summary of models for mothers. Generalized linear mixed models were fitted in a Bayesian framework using Markov chain Monte Carlo methods in the R package MCMCglmm version 2.34 ^1,2^. The point estimate of the posterior mean and 95% highest posterior density intervals (HPDI), effective sample size, and *p*MCMC value for each predictor variable are provided.

| **BKA (*S. saureus*) ~** | **Estimate** | **Effect** | **Mean [95% HPDI]** | **Effective *n*** | ***p*MCMC** |
| --- | --- | --- | --- | --- | --- |
| cortisol + beach | intercept |  | -0.19 [-0.48 – 0.09] | 1000 | 0.17 |
|  | cortisol | direct, total | 0.07 [ 0.05 - 0.10] | 1000 | <0.001 |
|  | beach: FWB | direct | -0.78 [-1.13 - -0.49] | 1136 | <0.001 |
| season + cortisol + beach | intercept |  | -0.38 [-0.72 - -0.06] | 1000 | 0.02 |
|  | season: 2019 | direct | 0.32 [0.01 – 0.63] | 1000 | 0.04 |
| season | intercept |  | -0.12 [-0.35 – 0.11] | 1000 | 0.31 |
|  | season: 2019 | total | 0.24 [-0.10 – 0.55] | 1000 | 0.14 |
| beach + season | intercept |  | 0.42 [0.18 – 0.70] | 1000 | 0.006 |
|  | beach: FWB | total | -1.06 [-1.36 - -0.72] | 891 | <0.001 |
| condition | intercept |  | -0.84 [-2.23 – 0.36] | 1000 | 0.21 |
|  | condition | n/a | 0.02 [-0.01 – 0.06] | 1090 | 0.20 |

| **BKA (*E. coli*) ~** | **Estimate** | **Effect** | **Mean [95% HPDI]** | **Effective *n*** | ***p*MCMC** |
| --- | --- | --- | --- | --- | --- |
| cortisol + beach | intercept |  | 0.37 [0.07 - 0.71] | 1000 | 0.03 |
|  | cortisol | direct, total | 0.01 [-0.01 – 0.03] | 1000 | 0.62 |
|  | beach: FWB | direct | -0.82 [-1.18 - -0.50] | 1000 | <0.001 |
| season + cortisol + beach | intercept |  | 0.10 [-0.26 – 0.46] | 1000 | 0.59 |
|  | season: 2019 | direct | 0.50 [0.16 – 0.83] | 1000 | 0.01 |
| season | intercept |  | -0.25 [-0.48 - -0.02] | 1000 | 0.04 |
|  | season: 2019 | total | 0.52 [0.20 – 0.85] | 1118 | <0.001 |
| beach + season | intercept |  | 0.18 [-0.09 – 0.42] | 752 | 0.15 |
|  | beach: FWB | total | -0.84 [-1.15 - -0.55] | 1000 | <0.001 |
| condition | intercept |  | 0.48 [-0.73 – 1.84] | 1000 | 0.48 |
|  | condition | n/a | -0.01 [-0.04 – 0.02] | 1000 | 0.49 |

| **hemagglutination ~** | **Estimate** | **Effect** | **Mean [95% HPDI]** | **Effective *n*** | ***p*MCMC** |
| --- | --- | --- | --- | --- | --- |
| cortisol + beach | intercept |  | 0.44 [0.09 - 0.81] | 1105 | 0.01 |
|  | cortisol | direct, total | -0.04 [-0.07 - -0.01] | 1316 | 0.006 |
|  | beach: FWB | direct | -0.21 [-0.63 - 0.19] | 1347 | 0.29 |
| season + cortisol + beach | intercept |  | 0.03 [-0.38 – 0.45] | 1000 | 0.88 |
|  | season:2019 | direct | 0.71 [0.31 – 1.09] | 1000 | 0.006 |
| season | intercept |  | -0.32 [-0.53 - -0.11] | 1000 | <0.001 |
|  | season: 2019 | total | 0.66 [0.32 – 0.95] | 1000 | <0.001 |
| beach + season | intercept |  | -0.22 [ -0.54 – 0.06] | 1000 | 0.15 |
|  | beach: FWB | total | -0.16 [-0.51 – 0.16] | 1000 | 0.33 |
| condition | intercept |  | 0.20 [-1.14 – 1.50] | 1000 | 0.76 |
|  | condition | n/a | -0.01 [-0.04 – 0.03] | 1000 | 0.76 |

| **hemolysis ~** | **Estimate** | **Effect** | **Mean [95% HPDI]** | **Effective *n*** | ***p*MCMC** |
| --- | --- | --- | --- | --- | --- |
| cortisol + beach | intercept |  | 0.41 [0.02 - 0.72] | 1000 | 0.01 |
|  | cortisol | direct, total | -0.04 [-0.07 - -0.01] | 1000 | 0.004 |
|  | beach: FWB | direct | -0.14 [-0.57 - 0.22] | 1000 | 0.52 |
| season + cortisol + beach | intercept |  | -0.16 [-0.56 – 0.21] | 1000 | 0.37 |
|  | season: 2019 | direct | 1.01 [0.63 – 1.34] | 962 | <0.001 |
| season | intercept |  | -0.44 [-0.64 - -0.21] | 1000 | 0.002 |
|  | season: 2019 | total | 0.91 [0.58 – 1.21] | 1000 | <0.001 |
| beach + season | intercept |  | -0.42 [-0.68 - -0.15] | 1000 | 0.004 |
|  | beach: FWB | total | -0.03 [-0.344 - 0.27] | 1000 | 0.84 |
| condition | intercept |  | 0.65 [-0.75 – 2.01] | 1000 | 0.36 |
|  | condition | n/a | -0.01 [-0.06 – 0.01] | 1000 | 0.34 |

| **lysozyme ~** | **Estimate** | **Effect** | **Mean [95% HPDI]** | **Effective *n*** | ***p*MCMC** |
| --- | --- | --- | --- | --- | --- |
| cortisol + beach | intercept |  | -0.10 [-0.47 - 0.23] | 1000 | 0.53 |
|  | cortisol | direct, total | 0.02 [0.00 - 0.05] | 1144 | 0.14 |
|  | beach: FWB | direct | -0.12 [-0.50 - 0.26] | 1136 | 0.54 |
| season + cortisol + beach | intercept |  | 0.06 [-0.32 – 0.47] | 1000 | 0.72 |
|  | season: 2019 | direct | -0.31 [-0.72 – 0.08] | 1124 | 0.09 |
| season | intercept |  | 0.11 [-0.12 – 0.34] | 1000 | 0.34 |
|  | season: 2019 | total | -0.23 [-0.60 – 0.07] | 1000 | 0.16 |
| beach + season | intercept |  | 0.19 [-0.11 – 0.49] | 1032 | 0.21 |
|  | beach: FWB | total | -0.15 [-0.49 – 0.20] | 1000 | 0.35 |
| condition | intercept |  | -0.25 [-1.62 – 1.13] | 1000 | 0.73 |
|  | condition | n/a | 0.01 [-0.03 – 0.04] | 1000 | 0.73 |

| **haptoglobin ~** | **Estimate** | **Effect** | **Mean [95% HPDI]** | **Effective *n*** | ***p*MCMC** |
| --- | --- | --- | --- | --- | --- |
| cortisol + beach | intercept |  | -0.17 [-0.55 - 0.18] | 1000 | 0.38 |
|  | cortisol | direct, total | 0.01 [-0.02 - 0.03] | 1000 | 0.70 |
|  | beach: FWB | direct | 0.23 [-0.17 - 0.63] | 1000 | 0.27 |
| season + cortisol + beach | intercept |  | -0.36 [-0.84 – 0.01] | 1000 | 0.07 |
|  | season: 2019 | direct | 0.35 [-0.01 – 0.80] | 1000 | 0.07 |
| season | intercept |  | -0.13 [-0.36 – 0.13] | 1206 | 0.28 |
|  | season: 2019 | total | 0.28 [-0.05 – 0.64] | 1000 | 0.09 |
| beach + season | intercept |  | -0.17 [-0.49 – 0.10] | 1000 | 0.26 |
|  | beach: FWB | total | 0.07 [-0.30 – 0.40] | 1053 | 0.67 |
| condition | intercept |  | 1.07 [-0.26 – 2.32] | 1000 | 0.11 |
|  | condition | n/a | -0.03 [-0.06 – 0.01] | 1000 | 0.11 |

| **neopterin ~** | **Estimate** | **Effect** | **Mean [95% HPDI]** | **Effective *n*** | ***p*MCMC** |
| --- | --- | --- | --- | --- | --- |
| cortisol + beach | intercept |  | -0.25 [-0.58 - 0.11] | 1000 | 0.14 |
|  | cortisol | direct, total | 0.02 [0.00 – 0.05] | 1000 | 0.06 |
|  | beach: FWB | direct | 0.24 [-0.17 – 0.65] | 1000 | 0.24 |
| season + cortisol + beach | intercept |  | -0.41 [-0.83 – 0.03] | 1321 | 0.06 |
|  | season: 2019 | direct | 0.27 [-0.09 – 0.70] | 1272 | 0.16 |
| season | intercept |  | -0.09 [-0.34 – 0.15] | 738 | 0.41 |
|  | season: 2019 | total | 0.18 [-0.19 – 0.49] | 1000 | 0.28 |
| beach + season | intercept |  | -0.12 [-0.40 – 0.16] | 1000 | 0.40 |
|  | beach: FWB | total | 0.06 [-0.27 – 0.38] | 1023 | 0.74 |
| condition | intercept |  | 0.47 [-0.87 – 1.71] | 1882 | 0.48 |
|  | condition | n/a | -0.01 [-0.04 – 0.02] | 1859 | 0.48 |

| **IgG ~** | **Estimate** | **Effect** | **Mean [95% HPDI]** | **Effective *n*** | ***p*MCMC** |
| --- | --- | --- | --- | --- | --- |
| cortisol + beach | intercept |  | 0.03 [-0.34 – 0.37] | 1000 | 0.86 |
|  | cortisol | direct, total | -0.01 [-0.04 – 0.02] | 899 | 0.59 |
|  | beach: FWB | direct | 0.05 [-0.37 – 0.45] | 1171 | 0.78 |
| season + cortisol + beach | intercept |  | -0.25 [-0.69 – 0.10] | 1103 | 0.22 |
|  | season: 2019 | direct | 0.52 [0.15 – 0.94] | 1156 | 0.01 |
| season | intercept |  | -0.26 [-0.49 - -0.03] | 1000 | 0.03 |
|  | season: 2019 | total | 0.52 [0.20 – 0.87] | 896 | <0.001 |
| beach + season | intercept |  | -0.29 [-0.58 – 0.01] | 1000 | 0.05 |
|  | beach: FWB | total | 0.07 [-0.24 – 0.41] | 1000 | 0.63 |
| condition | intercept |  | 0.05 [-1.27 – 1.27] | 1000 | 0.93 |
|  | condition | n/a | 0.00 [-0.03 – 0.03] | 1000 | 0.93 |

| **WBC (innate / adaptive) ~** | **Estimate** | **Effect** | **Mean [95% HPDI]** | **Effective *n*** | ***p*MCMC** |
| --- | --- | --- | --- | --- | --- |
| cortisol + beach | intercept |  | -0.32 [-0.67 – 0.03] | 887 | 0.09 |
|  | cortisol | direct, total | 0.04 [0.01 – 0.07] | 1000 | 0.002 |
|  | beach: FWB | direct | -0.00 [-0.43 – 0.39] | 1000 | 0.96 |
| season + cortisol + beach | intercept |  | -0.31 [-0.73 – 0.11] | 1000 | 0.14 |
|  | season: 2019 | direct | 0.00 [-0.40 – 0.39] | 1187 | 0.98 |
| season | intercept |  | 0.06 [-0.18 – 0.28] | 1107 | 0.62 |
|  | season: 2019 | total | -0.09 [-0.46 – 0.21] | 1203 | 0.57 |
| beach + season | intercept |  | 0.07 [-0.21 – 0.36] | 1000 | 0.65 |
|  | beach: FWB | total | -0.03 [-0.38 – 0.29] | 1000 | 0.83 |
| condition | intercept |  | -1.08 [-2.39 – 0.02] | 1000 | 0.08 |
|  | condition | n/a | 0.03 [-0.00 – 0.06] | 1000 | 0.08 |

| **dROM ~** | **Estimate** | **Mean [95% HPDI]** | **Effective *n*** | ***p*MCMC** |
| --- | --- | --- | --- | --- |
| BKA (*S. aureus*) | intercept | 0.01 [-0.15 – 0.18] | 1000 | 0.84 |
|  | BKA (*S. aureus*) | 0.05 [-0.09 – 0.19] | 1156 | 0.46 |
| BKA (*E. coli*) | intercept | 0.01 [-0.14 – 0.19] | 908 | 0.82 |
|  | BKA (*E. coli*) | 0.00 [-0.16 – 0.14] | 1000 | 0.97 |
| hemagglutination | intercept | 0.05 [-0.10 – 0.25] | 1000 | 0.56 |
|  | hemagglutination | -0.08 [-0.24 – 0.06] | 1000 | 0.25 |
| hemolysis | intercept | 0.05 [-0.13 – 0.22] | 1000 | 0.54 |
|  | hemolysis | -0.01 [-0.17 – 0.13] | 1000 | 0.92 |
| lysozyme | intercept | 0.01 [-0.15 – 0.19] | 1000 | 0.88 |
|  | lysozyme | 0.00 [-0.16 – 0.14] | 1000 | 0.97 |
| haptoglobin | intercept | 0.01 [-0.13 – 0.19] | 1000 | 0.87 |
|  | haptoglobin | 0.27 [0.14 – 0.43] | 1119 | <0.001 |
| neopterin | intercept | 0.05 [-0.11 – 0.23] | 1000 | 0.55 |
|  | neopterin | -0.09 [-0.24 – 0.04] | 1000 | 0.20 |
| IgG | intercept | 0.01 [-0.18 – 0.16] | 1000 | 0.88 |
|  | IgG | 0.06 [-0.07 – 0.22] | 1000 | 0.36 |
| WBC count (innate / adaptive) | intercept | 0.03 [-0.12 – 0.21] | 1000 | 0.73 |
|  | ratio | 0.09 [-0.05 – 0.23] | 1000 | 0.19 |

| **OXY ~** | **Estimate** | **Mean [95% HPDI]** | **Effective *n*** | ***p*MCMC** |
| --- | --- | --- | --- | --- |
| BKA (*S. aureus*) | intercept | 0.00 [-0.18 – 0.14] | 1000 | 0.99 |
|  | BKA (*S. aureus*) | 0.03 [-0.10 – 0.19] | 1000 | 0.63 |
| BKA (*E. coli*) | intercept | 0.00 [-0.15 – 0.16] | 894 | 0.95 |
|  | BKA (*E. coli*) | 0.04 [-0.11 – 0.20] | 1099 | 0.56 |
| hemagglutination | intercept | 0.03 [-0.12 – 0.22] | 1066 | 0.65 |
|  | hemagglutination | 0.09 [-0.06 – 0.23] | 1000 | 0.22 |
| hemolysis | intercept | 0.03 [-0.13 – 0.20] | 1000 | 0.68 |
|  | hemolysis | 0.09 [-0.05 – 0.25] | 1000 | 0.25 |
| lysozyme | intercept | 0.00 [-0.16 – 0.15] | 1000 | 0.99 |
|  | lysozyme | 0.13 [-0.01 – 0.27] | 1234 | 0.08 |
| haptoglobin | intercept | 0.00 [-0.15 – 0.17] | 1000 | 0.95 |
|  | haptoglobin | 0.32 [0.16 – 0.45] | 1000 | <0.001 |
| neopterin | intercept | 0.02 [-0.14 – 0.20] | 945 | 0.72 |
|  | neopterin | 0.10 [-0.03 – 0.27] | 1252 | 0.15 |
| IgG | intercept | 0.00 [-0.14 – 0.17] | 1000 | 0.96 |
|  | IgG | 0.15 [0.00 – 0.28] | 1000 | 0.04 |
| WBC count (innate / adaptive) | intercept | 0.01 [-0.15 – 0.19] | 1000 | 0.90 |
|  | ratio | -0.02 [-0.16 – 0.12] | 1000 | 0.78 |

| **GPx ~** | **Estimate** | **Mean [95% HPDI]** | **Effective *n*** | ***p*MCMC** |
| --- | --- | --- | --- | --- |
| BKA (*S. aureus*) | intercept | 0.02 [-0.19 – 0.22] | 1000 | 0.83 |
|  | BKA (*S. aureus*) | 0.12 [-0.10 – 0.30] | 1000 | 0.29 |
| BKA (*E. coli*) | intercept | 0.05 [-0.16 – 0.27] | 813 | 0.65 |
|  | BKA (*E. coli*) | 0.24 [0.08 – 0.40] | 1000 | 0.006 |
| hemagglutination | intercept | -0.03 [-0.24 – 0.17] | 1000 | 0.78 |
|  | hemagglutination | 0.06 [-0.12 – 0.22] | 893 | 0.52 |
| hemolysis | intercept | -0.03 [-0.26 – 0.17] | 1000 | 0.77 |
|  | hemolysis | 0.09 [-0.13 – 0.28] | 1000 | 0.41 |
| lysozyme | intercept | -0.02 [-0.20 – 0.23] | 1000 | 0.87 |
|  | lysozyme | -0.18 [-0.41 – 0.07] | 1000 | 0.16 |
| haptoglobin | intercept | 0.00 [-0.21 – 0.22] | 1000 | 0.97 |
|  | haptoglobin | -0.02 [-0.22 – 0.22] | 1000 | 0.89 |
| neopterin | intercept | -0.02 [-0.26 – 0.17] | 1000 | 0.82 |
|  | neopterin | -0.08 [-0.28 – 0.07] | 1000 | 0.35 |
| IgG | intercept | -0.03 [-0.23 – 0.19] | 1207 | 0.85 |
|  | IgG | -0.34 [-0.57 – -0.11] | 1107 | 0.004 |
| WBC count (innate / adaptive) | intercept | -0.02 [-0.23 – 0.19] | 793 | 0.81 |
|  | ratio | 0.05 [-0.11 – 0.22] | 1000 | 0.54 |

| **SOD ~** | **Estimate** | **Mean [95% HPDI]** | **Effective *n*** | ***p*MCMC** |
| --- | --- | --- | --- | --- |
| BKA (*S. aureus*) | intercept | 0.01 [-0.22 – 0.23] | 890 | 0.88 |
|  | BKA (*S. aureus*) | 0.05 [-0.17 – 0.26] | 910 | 0.61 |
| BKA (*E. coli*) | intercept | -0.01 [-0.27 – 0.21] | 1000 | 0.92 |
|  | BKA (*E. coli*) | -0.12 [-0.29 – 0.08] | 1000 | 0.19 |
| hemagglutination | intercept | -0.01 [-0.22 – 0.22] | 1000 | 0.89 |
|  | hemagglutination | -0.13 [-0.34 – 0.09] | 1000 | 0.24 |
| hemolysis | intercept | -0.03 [-0.26 – 0.19] | 1000 | 0.80 |
|  | hemolysis | -0.30 [-0.50 – -0.05] | 1000 | 0.01 |
| lysozyme | intercept | -0.09 [-0.11 – 0.28] | 1284 | 0.41 |
|  | lysozyme | 0.65 [0.42 – 0.89] | 1000 | < 0.001 |
| haptoglobin | intercept | -0.01 [-0.22 – 0.23] | 1000 | 0.88 |
|  | haptoglobin | -0.23 [-0.48 – 0.01] | 1000 | 0.08 |
| neopterin | intercept | -0.00 [-0.23 – 0.24] | 815 | 0.98 |
|  | neopterin | -0.07 [-0.09 – 0.24] | 1000 | 0.39 |
| IgG | intercept | -0.01 [-0.24 – 0.21] | 1000 | 0.94 |
|  | IgG | -0.16 [-0.53 – -0.13] | 1000 | 0.36 |
| WBC count (innate / adaptive) | intercept | 0.01 [-0.20 – 0.22] | 1000 | 0.88 |
|  | ratio | 0.26 [0.01 – 0.50] | 892 | 0.05 |

1. Hadfield, J. D. MCMC methods for multi-response generalized linear mixed models: The MCMCglmm R package. *Journal of Statistical Software* **33**, 1–22 (2010).

2. Hadfield, J. Markov chain Monte Carlo generalised linear mixed models - Course Notes. (2019).
