# Supplementary material for: Life-history stage influences immune investment and oxidative stress in response to environmental heterogeneity in Antarctic fur seals
Source: Commun Biol. 2024 Jun 29;7:788. doi: 10.1038/s42003-024-06499-6 (PMC11217341; doi:10.1038/s42003-024-06499-6)
Supplement: Supplementary file 5 — Reporting Summary [file 42003_2024_6499_MOESM5_ESM.pdf]

Corresponding author(s): Rebecca Nagel
Last updated by author(s): May 17, 2024

Reporting Summary

Nature Portfolio wishes to improve the reproducibility of the work that we publish. This form provides structure for consistency and transparency in reporting. For further information on Nature Portfolio policies, see our Editorial Policies and the Editorial Policy Checklist.

Statistics

For all statistical analyses, confirm that the following items are present in the figure legend, table legend, main text, or Methods section.

n/a	Confirmed	
☐	☒	The exact sample size (*n*) for each experimental group/condition, given as a discrete number and unit of measurement
☐	☒	A statement on whether measurements were taken from distinct samples or whether the same sample was measured repeatedly
☐	☒	The statistical test(s) used AND whether they are one- or two-sided<br>*Only common tests should be described solely by name; describe more complex techniques in the Methods section.*
☐	☒	A description of all covariates tested
☐	☒	A description of any assumptions or corrections, such as tests of normality and adjustment for multiple comparisons
☐	☒	A full description of the statistical parameters including central tendency (e.g. means) or other basic estimates (e.g. regression coefficient) AND variation (e.g. standard deviation) or associated estimates of uncertainty (e.g. confidence intervals)
☐	☒	For null hypothesis testing, the test statistic (e.g. F, t, r) with confidence intervals, effect sizes, degrees of freedom and P value noted<br>*Give P values as exact values whenever suitable.*
☐	☒	For Bayesian analysis, information on the choice of priors and Markov chain Monte Carlo settings
☐	☒	For hierarchical and complex designs, identification of the appropriate level for tests and full reporting of outcomes
☐	☒	Estimates of effect sizes (e.g. Cohen's d, Pearson's r), indicating how they were calculated

Our web collection on statistics for biologists contains articles on many of the points above.

Software and code

Policy information about availability of computer code

Data collection	no software was used for data collection
Data analysis	we ran all analyses in R version 4.2.1

For manuscripts utilizing custom algorithms or software that are central to the research but not yet described in published literature, software must be made available to editors and reviewers. We strongly encourage code deposition in a community repository (e.g. GitHub). See the Nature Portfolio guidelines for submitting code & software for further information.

Data

Policy information about availability of data

All manuscripts must include a data availability statement. This statement should provide the following information, where applicable:

- Accession codes, unique identifiers, or web links for publicly available datasets
- A description of any restrictions on data availability
- For clinical datasets or third party data, please ensure that the statement adheres to our policy

All raw data and code can be found at: https://doi.org/10.5281/zenodo.11208287.

Research involving human participants, their data, or biological material

Policy information about studies with human participants or human data. See also policy information about sex, gender (identity/presentation), and sexual orientation and race, ethnicity and racism.

Reporting on sex and gender	N/A
Reporting on race, ethnicity, or other socially relevant groupings	N/A
Population characteristics	N/A
Recruitment	N/A
Ethics oversight	N/A

Note that full information on the approval of the study protocol must also be provided in the manuscript.

Field-specific reporting

Please select the one below that is the best fit for your research. If you are not sure, read the appropriate sections before making your selection.

☐ Life sciences ☐ Behavioural & social sciences ☒ Ecological, evolutionary & environmental sciences

For a reference copy of the document with all sections, see nature.com/documents/nr-reporting-summary-flat.pdf

Ecological, evolutionary & environmental sciences study design

All studies must disclose on these points even when the disclosure is negative.

Study description	We collected data during two, five-month long field seasons in 2018-19 and 2019-20 on Bird Island, South Georgia. Radio telemetry (VHF) was used to track the survival of a total of 100 focal pups from two breeding colonies from birth (day 0) until molting (approx. day 60). Biometric data was collected every 10 days, saliva was collected every 20 days, and blood samples were collected at birth and molt. To account for maternal effects, mothers (n=100) were also tracked using radio telemetry and sampled concurrently with their pups at birth and molt.
Research sample	A population of Antarctic fur seals (Arctocephalus gazella) from Bird Island, South Georgia. This population has been monitored by the British Antarctic Survey since the 1980s, providing detailed information on population dynamics. Animal handling protocols are well established and refined by the British Antarctic Survey ensuring sample collection was ethical and feasible.
Sampling strategy	A total of 100 focal mother-pup pairs were sampled for this study (i.e. a total of 200 individuals = 100 mothers + 100 pups). 50 pairs were sampled in 2018-19, and 50 pairs were sampled in 2019-20. Sample size was determined by the British Antarctic Survey Animal Welfare and Ethics Review Body based on feasibility and effect size. Mothers were all fully mature, sexually reproducing adult females. Pups were sampled from birth until molt, at around 60 days of age. Sampling was randomized with respect to pup sex.
Data collection	Adult females were captured with a noosing pole and immobilized on a restraint board. Pups were captured with a slip noose or by hand and were restrained by hand. Saliva was collected by rotating sterile cotton tip applicators fitted in polypropylene tubes (ROTH, Art. No. XC10.2) in the cheek pouch and under the tongue. 2.5 mL of blood was collected from the hind flipper using BD Discardit Eccentric Luer-Slip two-piece syringes and BD Microlance stainless steel needles (25 G, 0.5 x 25 mm). Data was recorded by one of four field researchers: Claire Stainfield or Rebecca Nagel (2019 and 2020), Camille Toscani (2019), or Cameron Fox-Clarke (2020).
Timing and spatial scale	Antarctic fur seals have a succinct pupping season. During both the 2018-19 and 2019-20 seasons, our sampling began shortly after the first pup was born on the breeding beach (early December). Monitoring and sampling of focal individuals continued until an individual died or the pup reached 60 days of age. Sampling duration (approx. 60 days) is the approximate time between birth and molting, when pups begin to gain nutritional independence. Mother-pup pairs at two breeding colonies - Freshwater Beach (FWB) and Special Study Beach (SSB) - were included in our sampling scheme. Initial sampling occurred on the breeding beaches. About 30 days after birth, mothers and pups begin to move off the beaches and further inland such that later monitoring (VHF) and sampling occurred across the island.
Data exclusions	No data was excluded from the analysis.
Reproducibility	For saliva, all samples were determined in duplicate and if the coefficient of variation was larger than 10%, determination of the sample was repeated. For blood smears, a random selection of 5% of slides were re-analyzed; ICC values were indicative of moderate to excellent repeatability. For BKA (E. coli and S. aureus), haptoglobin, neopterin, and IgG assays, all samples were assessed in duplicate. For lysozyme, the clearing zone of each sample was measured at three different angles and the average was taken for downstream analysis. Also, on each agar plate we ran standards. On any given day, plates standards were from the same tubes and they were freshly made each day. For hemagglutination and hemolysis, we used one pool (made out of 40 samples) as an internal control on 12 of 24 plates on the first day and 11 of 25 plates on the second day.

Randomization	Focal individuals were allocated into groups based on their location of breeding (mothers) / birth (pups). Focal individuals were also grouped by year of breeding / birth.
Blinding	Field data collection and laboratory work for saliva and blood samples were done by different groups of people. We randomized the samples before starting any assays. Data checking was blinded.

Did the study involve field work? ☒ Yes ☐ No

Field work, collection and transport

Field conditions	Field work was conducted during the austral summer. 2018-19 mean temperature = 3.7 °C ± s.d. 1.3; 2019-20 mean temperature = 3.9 °C ± s.d. 1.4. 2018-19 mean wind speed = 9.7 knots ± s.d. 5.1; 2019-20 mean wind speed = 9.3 knots ± s.d. 4.8. Work was carried out in all weather conditions.
Location	Bird Island, South Georgia (54°00′24.8″S, 38°03′04.1″W), a sub-Antarctic island located in the southern Atlantic Ocean
Access & import/export	Sampling was carried out by the British Antarctic Survey under permits from the Government of South Georgia and the South Sandwich Islands (Wildlife and Protected Areas Ordinance (2011), RAP permit numbers 2018/024 and 2019/032). The samples were imported into the UK under permits from the Department for Environment, Food and Rural Affairs (Animal Health Act, import license number ITIMP18.1397) and from the Convention on International Trade in Endangered Species of Wild Fauna and Flora (import numbers 578938/01-15 and 590196/01-18). The Nagoya protocol has not been extended to South Georgia & the South Sandwich Islands.
Disturbance	The sampling protocol caused no adverse effects on the Antarctic fur seal population. Only drawing blood could be considered an invasive procedure and this was limited to two, well-spaced time points (approximately 60 days) during the season. All protocols used in this study were approved by the British Antarctic Survey AWERB (Animal Welfare and Ethics Review Body) and are constantly under review, aimed at minimizing individual and population impacts.

Reporting for specific materials, systems and methods

We require information from authors about some types of materials, experimental systems and methods used in many studies. Here, indicate whether each material, system or method listed is relevant to your study. If you are not sure if a list item applies to your research, read the appropriate section before selecting a response.

Materials & experimental systems

n/a	Involved in the study
☒	☐ Antibodies
☒	☐ Eukaryotic cell lines
☒	☐ Palaeontology and archaeology
☐	☒ Animals and other organisms
☒	☐ Clinical data
☒	☐ Dual use research of concern
☒	☐ Plants

Methods

n/a	Involved in the study
☒	☐ ChIP-seq
☒	☐ Flow cytometry
☒	☐ MRI-based neuroimaging

Animals and other research organisms

Policy information about studies involving animals; ARRIVE guidelines recommended for reporting animal research, and Sex and Gender in Research

Laboratory animals	The study did not involve laboratory animals.
Wild animals	A wild population of Antarctic fur seals (Arctocephalus gazella). Mothers were of varying (unknown) age, but all sexually mature, reproducing females. Pups were sampled 2-3 days after birth and monitored until approximately 60 days of age. Adult females were captured with a noosing pole and immobilized on a restraint board. Pups were captured with a slip noose or by hand and were restrained by hand. Individuals were released as close to their capture site as possible and the pups were reunited with their mothers.
Reporting on sex	Sampling was randomized with respect to pup sex, resulting in a total of 51 male and 49 female focal pups. Sex was assigned by visual inspection of external genitalia. Sex was included as an explanatory factor in our statistical models.
Field-collected samples	Samples were stored at -20°C in the field and during transport. Blood samples were transferred to -80°C in the laboratory. Any samples remaining after quantification are stored at Bielefeld University., Germany

Ethics oversight	All procedures used were approved by the British Antarctic Survey Animal Welfare and Ethics Review Body (AWERB applications 2018/1050 and 2019/1058).

Note that full information on the approval of the study protocol must also be provided in the manuscript.
